# Supplementary material for: Genetically variant human pluripotent stem cells selectively eliminate wild-type counterparts through YAP-mediated cell competition
Source: Dev Cell. 2021 Sep 13;56(17):2455–2470.e10. doi: 10.1016/j.devcel.2021.07.019 (PMC8443275; doi:10.1016/j.devcel.2021.07.019)
Supplement: Document S1. Figures S1–S7 [file mmc1.pdf]

Developmental Cell, Volume *56*

Supplemental information

Genetically variant human pluripotent stem cells selectively eliminate wild-type counterparts through YAP-mediated cell competition

Christopher J. Price, Dylan Stavish, Paul J. Gokhale, Ben A. Stevenson, Samantha Sargeant, Joanne Lacey, Tristan A. Rodriguez, and Ivana Barbaric

Supplementary Figure 1

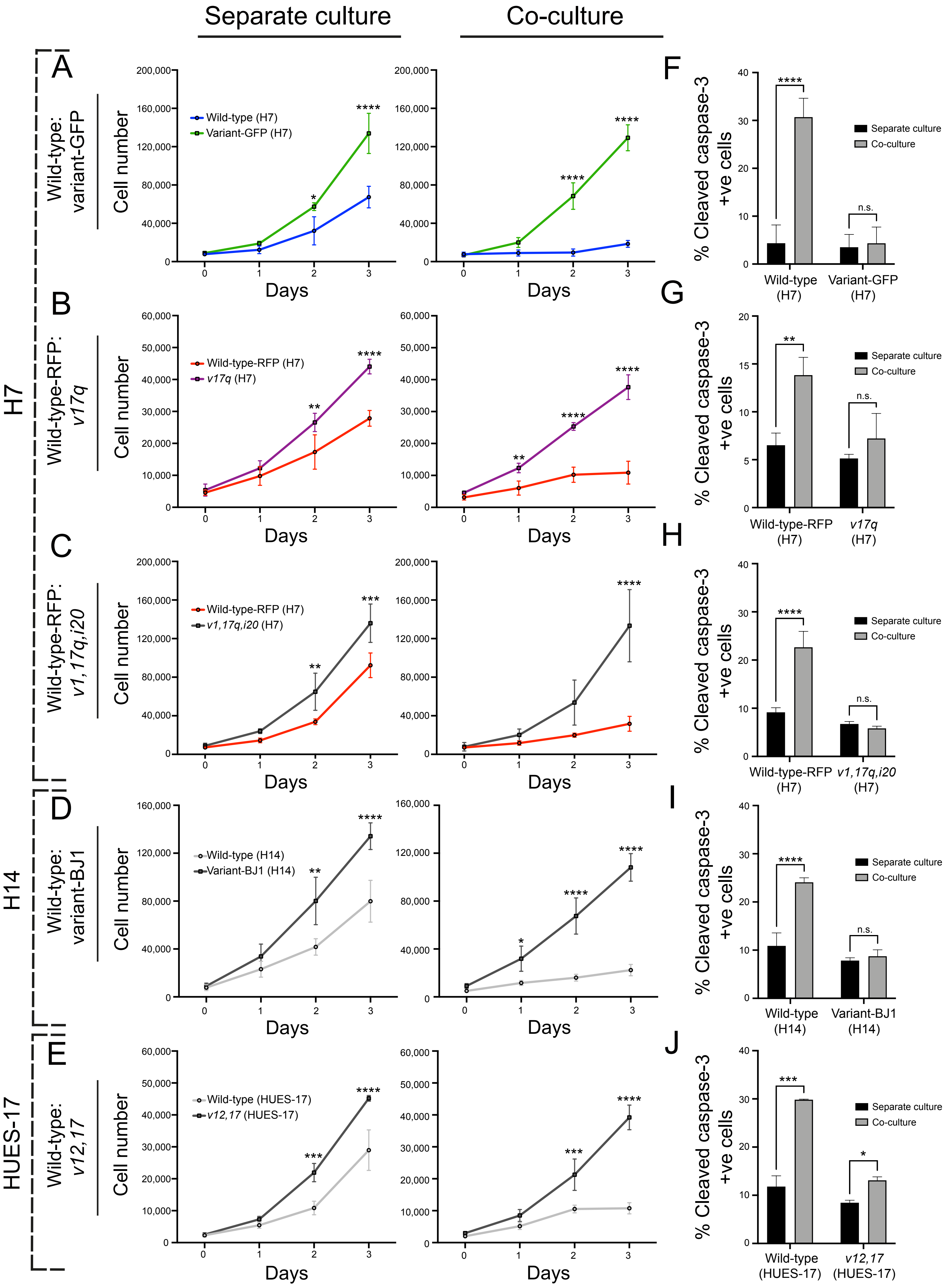

Figure S1. Wild-type cells are eliminated from co-cultures with variant hPSCs. Related to Figure 1.

A – E. Growth curves of wild-type and variant sublines of H7 (A-C), H14 (D), and HUES-17 (E) hPSC lines grown separately and in co-culture. Number of fields acquired in the well: entire well (A,C,D) or 16 random fields (B,E).

F – J. Percentage of cells positive for cleaved caspase-3 indicator of apoptosis in wild-type and variant sublines of H7 (F-H), H14 (I), and HUES-17 (J) hPSC lines grown separately or upon co-culture.

Data are the mean of three (A-I) or two (J) independent experiments ± SD. n.s. non-significant; *$p < 0.05$; **$p < 0.01$; ***$p < 0.001$; ****$p < 0.0001$, two-way ANOVA, followed by Holm-Sidak's multiple comparisons test.

Supplementary Figure 2

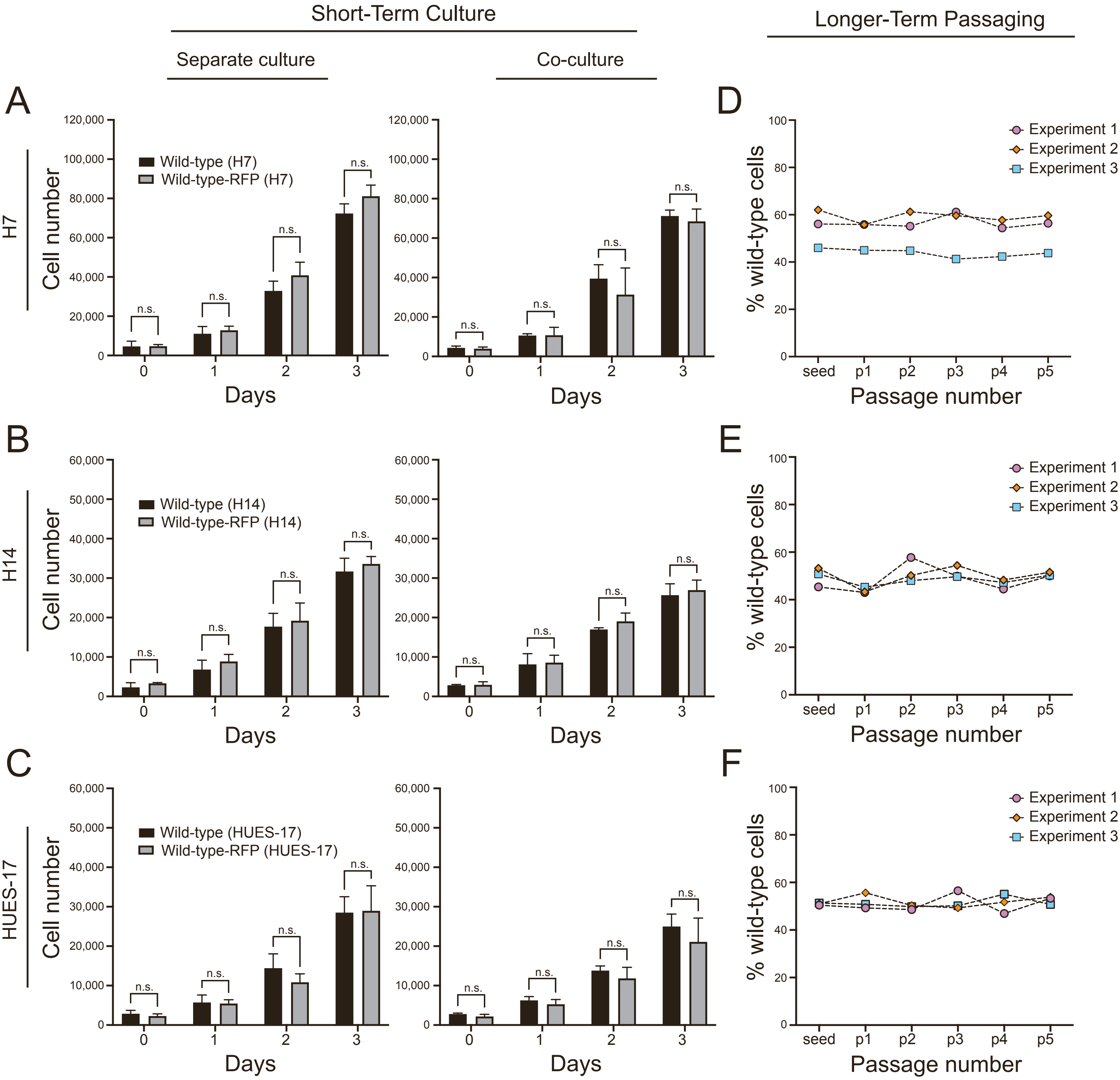

Figure S2. Wild-type hPSCs are not eliminated from co-cultures with wild-type-RFP counterparts. Related to Figure 1.

A - C. Growth curves of wild-type and wild-type-RFP sublines of H7 (A), H14 (B), and HUES-17 (C) hPSC lines grown separately and in co-culture over short-term three day culture period. Number of fields acquired in the well: entire well (A) or 16 random fields (B,C). Data represent the mean of three independent experiments ± SD. n.s. non-significant; two-way ANOVA followed by Holm-Sidak's multiple comparisons test.

D – F. Percentage of wild-type cells grown in co-cultures with wild-type-RFP cells of H7 (D), H14 (E), and HUES-17 (F) hPSC lines over five sequential passages. Data from three independent experiments are shown.

Supplementary Figure 3

A

Wild-type-RFP cells in co-culture with variant-GFP hPSCs

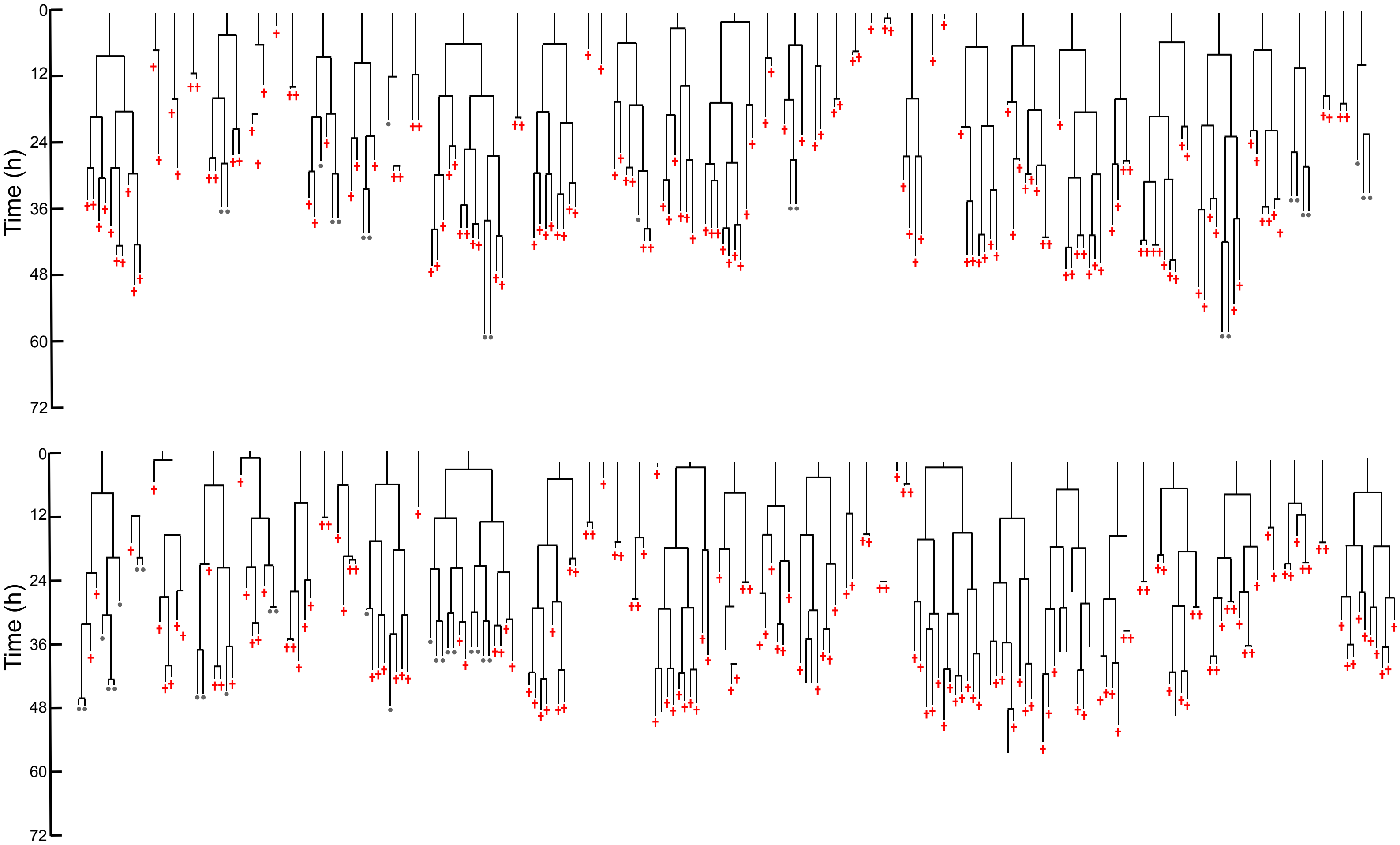

B

Wild-type-RFP cells in co-culture with wild-type hPSCs

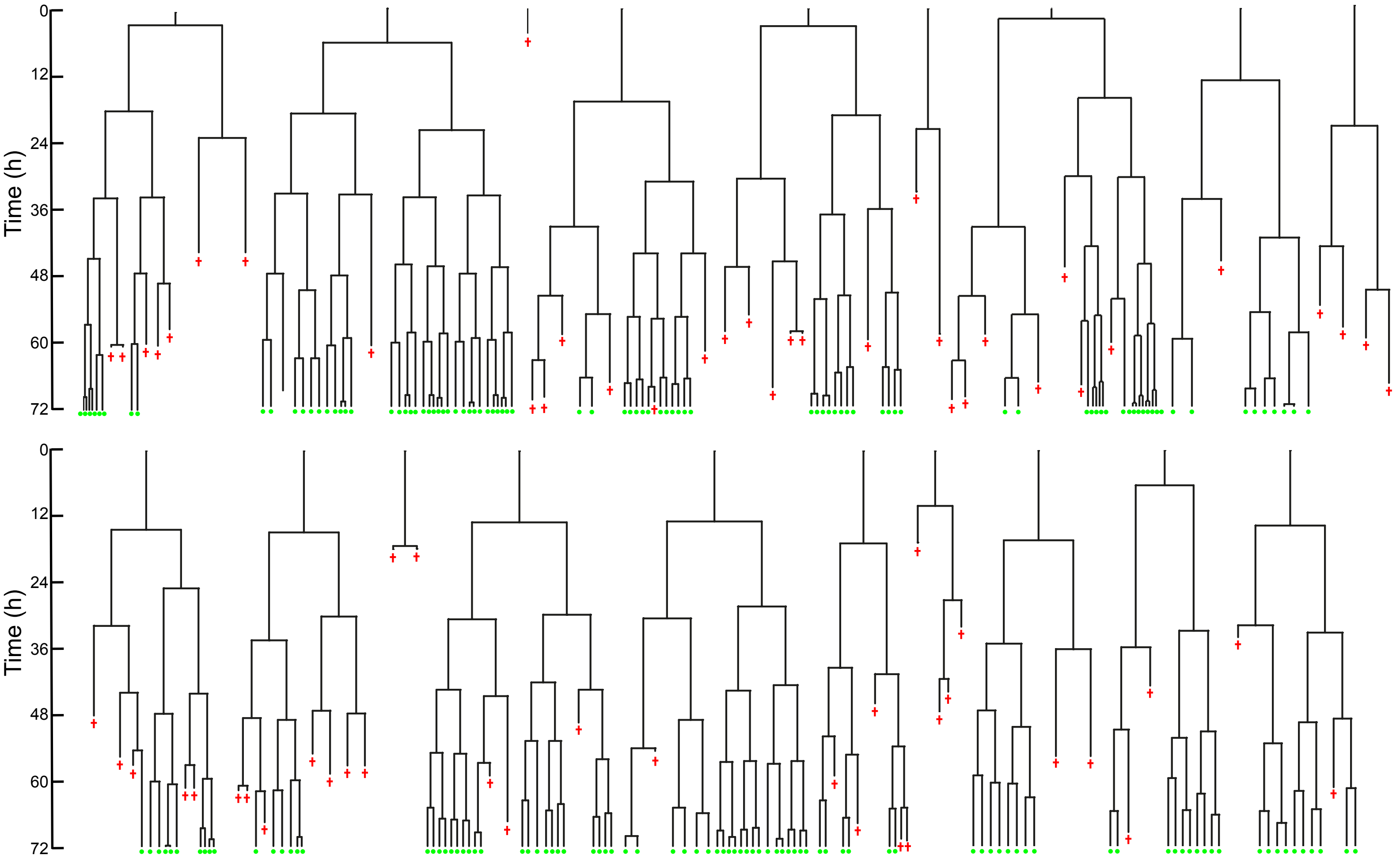

Figure S3. Lineage trees of wild-type-RFP cells co-cultured with either wild-type or variant-GFP cells. Related to Figure 1.

A-B. Lineages trees of individual wild-type-RFP cells tracked from day 0 to day 3 of co-culture with (A) variant-GFP cells or (B) wild-type-RFP. Scale indicates time in hours. Green circles indicate cells that survived and grey circles indicate cells that could no longer be accurately tracked. Red crosses denote cell death. A total of 78 (A) and 19 (B) lineage trees are shown.

Supplementary Figure 4

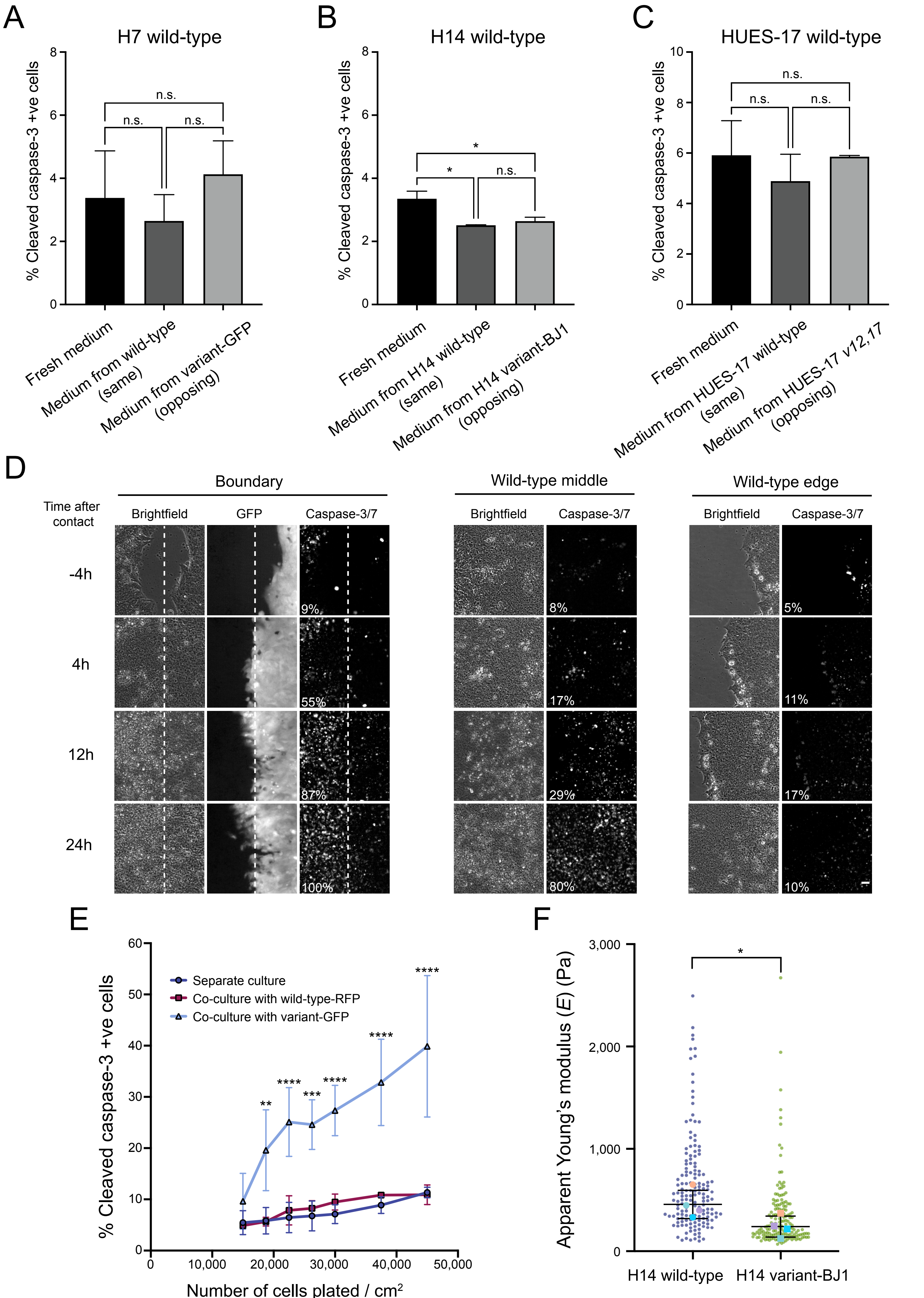

Figure S4. Winner phenotype is mediated through cell contact dependent mechanisms. Related to Figure 2 and Figure 3.

A-C. Percentage of wild-type cells positive for cleaved caspase-3 indicator of apoptosis in H7 (A), H14 (B) and HUES-17 (C) hPSC lines in conditioned media experiments. Cells were incubated either in new media, media conditioned on corresponding wild-type cells or in media conditioned by variant sublines that display a competitive phenotype. Data are the mean of three (A) or two (B,C) independent experiments ± SD. n.s. non-significant, $*p<0.05$; one-way ANOVA followed by Holm-Sidak's multiple comparisons test.

D. Frozen frames from the time-lapse imaging of cell confrontation assay of wild-type and variant-GFP cells with live caspase-3/7 dye. Left panel: region of the inserts where wild-type and variant-GFP cells come into contact. Dashed white line indicates the position on the insert where the two different populations meet. Middle panel: middle region of the wild-type cell population on the inserts. Right panel: edge of the wild-type cell population on the inserts. The percentage of wild-type cell area covered by live-caspase signal is indicated in the bottom-left of each image.

E. Percentage of wild-type cells positive for cleaved caspase-3 indicator of apoptosis grown either in separate culture or in co-culture with wild-type-RFP or variant-GFP cells at increasing plating densities. Data are the mean of three independent experiments ± SD. $**p<0.01$; $***p<0.001$; $****p<0.0001$; two-way ANOVA followed by Holm-Sidak's multiple comparisons test.

F. Apparent Young's Modulus (*E*) of H14 wild-type and H14 variant-BJ1 cells as measured by AFM. Small blue (wild-type) and green (variant-BJ1) points indicate individual cells, larger points indicate mean ± SD from 4 independent experiments. $*p< 0.05$; Student's *t* test.

Supplementary Figure 5

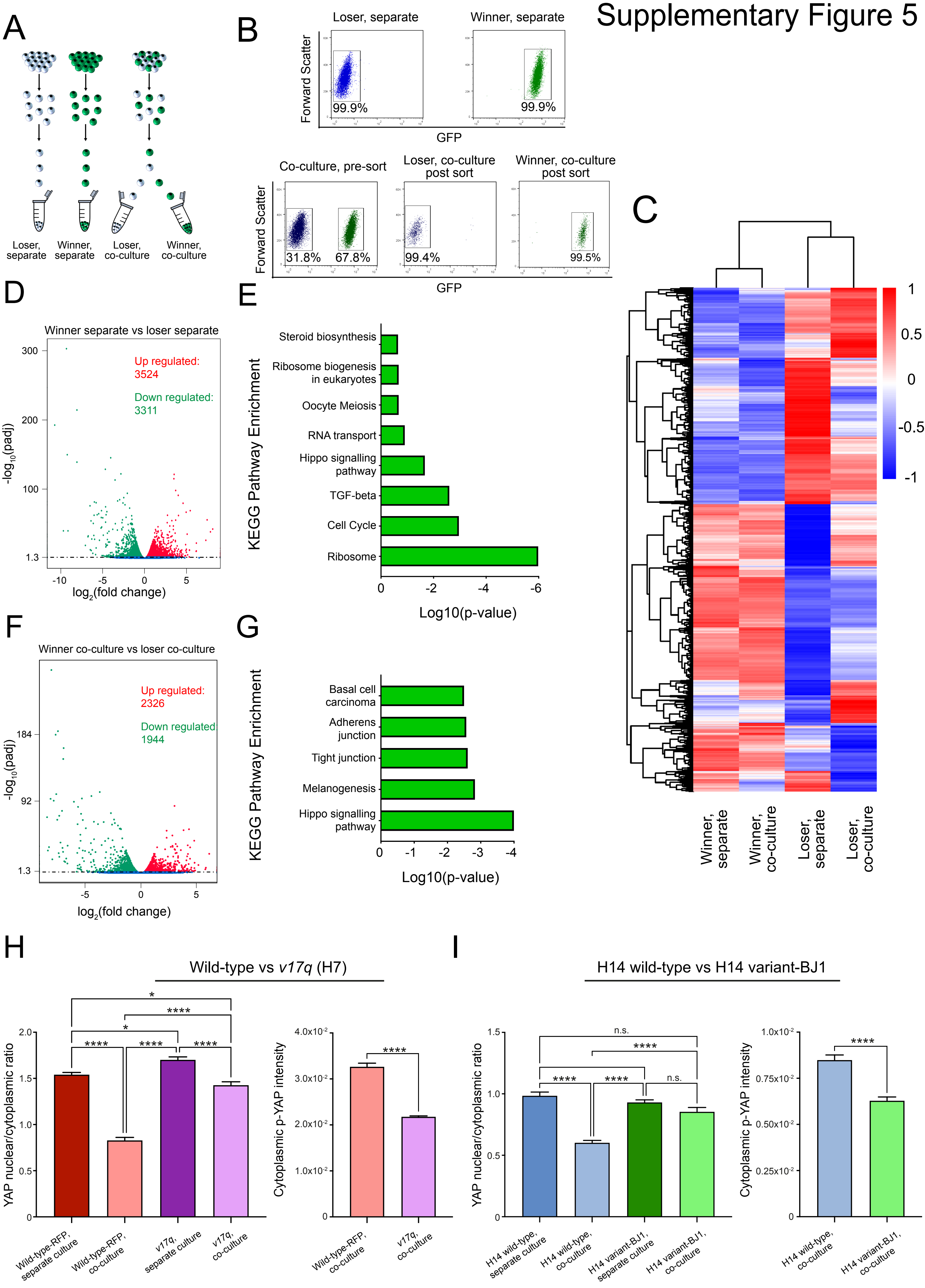

Figure S5. Gene expression analysis and immunohistochemistry of winner and loser cells indicates Hippo signalling and YAP as mediators of cell competition in hPSC cultures. Related to Figure 5.

A. Schematic depicting the sorting of loser (*v1q*) and winner (variant-GFP) cells from separate or co-culture conditions to obtain the following populations: 'loser separate', 'winner separate', 'loser co-culture' and 'winner co-culture'. Four biological replicates of each sample were obtained from independent experiments.

B. Representative flow cytometry plots of loser and winner cell populations isolated from separate or co-cultures for RNA sequencing.

C. Unsupervised hierarchical clustering of the winner and loser cells from separate and co-culture conditions based on the differentially expressed genes.

D. Volcano plot of the differentially expressed genes between winner and loser hPSCs in separate cultures. Downregulated genes (green) are positioned on the left of the plot and upregulated genes (red) are on the right of the plot.

E. KEGG pathway analysis of the downregulated genes in winner versus loser hPSCs in separate cultures.

F. Volcano plot of the differentially expressed genes between winner and loser hPSCs in co-culture. Downregulated genes (green) are positioned on the left of the plot and upregulated gene (red) are on the right of the plot.

G. KEGG pathway analysis of the downregulated genes in winner versus loser hPSCs in co-culture.

H-I. The nuclear to cytoplasmic ratio of YAP and cytoplasmic p-YAP intensity in H7 wild-type-RFP and *v17q* (H), and H14 wild-type and H14 variant-BJ1 (I) grown separately or upon co-culture. Data is represented as the mean ± SEM. *$p < 0.05$; ****$p < 0.0001$; one-way ANOVA followed by Kruskal-Wallis multiple comparisons test.

Supplementary Figure 6

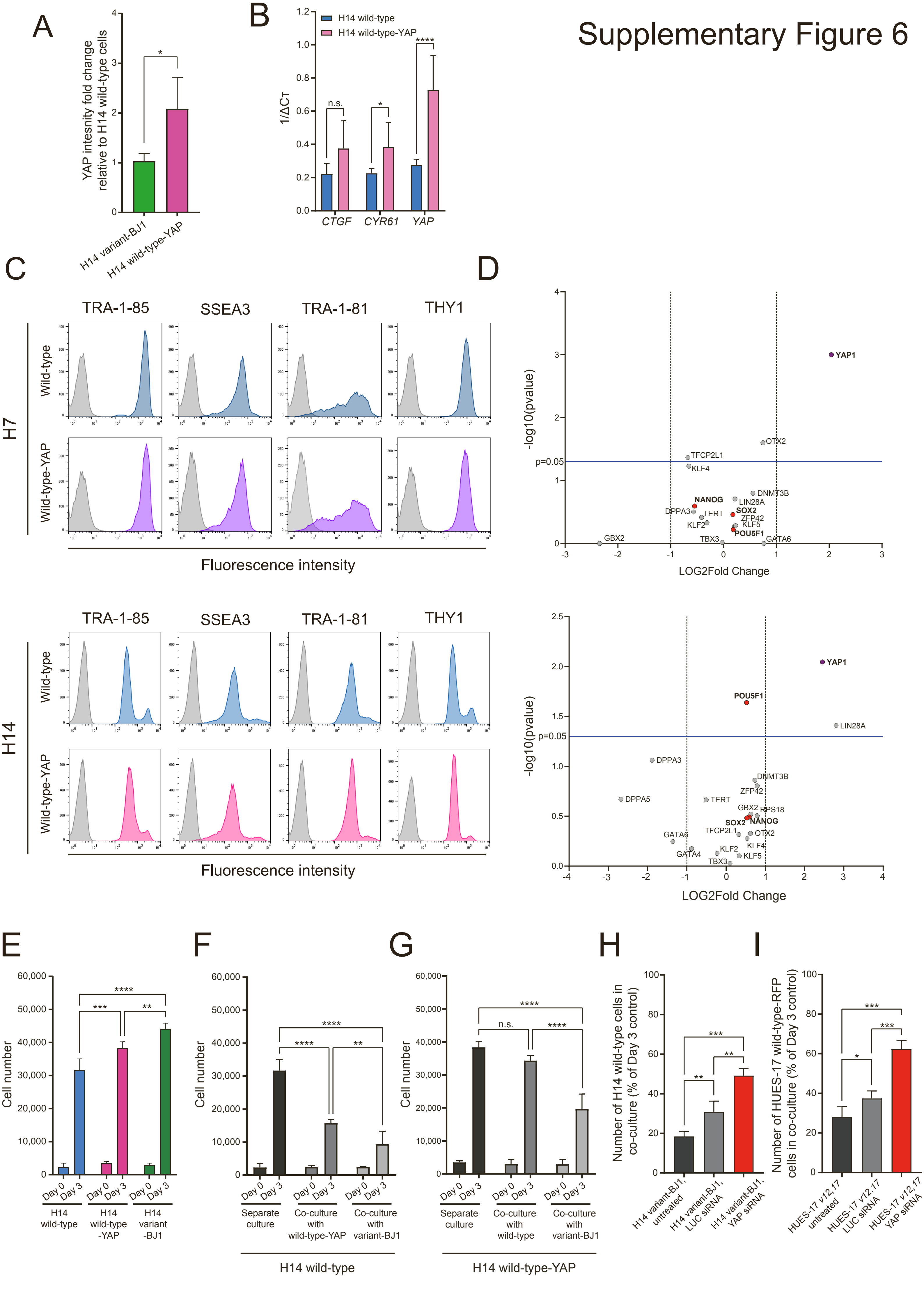

Figure S6. Manipulation of YAP affects the winner-loser phenotype, but not the pluripotency state of hPSCs. Related to Figure 5.

A. YAP overexpression in H14 wild-type hPSCs. YAP staining intensity in H14 variant-BJ1 and H14 wild-type-YAP overexpressing cells relative to the YAP staining intensity of H14 wild-type cells, as determined by flow cytometry. Data are the mean of three independent experiments ± SD. *p< 0.05; Student's *t* test.

B. Expression of YAP and its targets gene is upregulated in H14 wild-type-YAP overexpressing cells compared to H14 wild-type hPSCs. Data are the mean of three independent experiments ± SD. n.s. non-significant, *p<0.05; ****p<0.0001; one-way ANOVA followed by Fisher's LSD test.

C. YAP overexpressing cells display similar expression profiles of stem cell-associated antigens to wild-type counterparts. Flow cytometry histograms of wild-type and wild-type-YAP overexpressing cells of H7 and H14 hPSC lines for the stem cell associated antigens SSEA3, TRA-1-81 and THY1. TRA-1-85 was used as a positive control. The negative control P3X (grey) is shown in each histogram.

D. Volcano plots of the expression levels of pluripotency-associated and naïve-associated genes in wild-type-YAP overexpressing cells compared to wild-type cells of H7 and H14 hPSC lines. The core pluripotency transcription factors *SOX2*, *NANOG* and *POUF51* are denoted by red circles. Blue line indicates p=0.05 and black dashed lines indicate log2 fold change of -1 and 1.

E. YAP overexpression leads to improved growth of H14 wild-type cells. Data are the mean of three independent experiments ± SD. **p<0.01; ***p<0.001; ****p<0.0001; two-way ANOVA followed by Holm-Sidak's multiple comparisons test.

F-G. YAP overexpression confers the winner phenotype in co-cultures with H14 wild-type cells (F) and increased resistance to cell crowding in co-cultures with H14 variant-

BJ1 cells (G). Data was acquired by imaging 16 random fields within a well. Data are the mean of three independent experiments ± SD. n.s. non-significant; **$p<0.01$; ****$p<0.0001$; two-way ANOVA followed by Holm-Sidak's multiple comparisons test.

H-I. Knockdown of YAP in H14 variant-BJ1 cells (H) and HUES-17 *v12,17* cells (I) partially alleviates the winner cell phenotype of variant cells. Data are the mean of technical triplicates ± SD. n.s. non-significant; *$p<0.05$; **$p<0.01$; ***$p<0.001$; one-way ANOVA followed by Holm-Sidak's multiple comparisons test.

Supplementary Figure 7

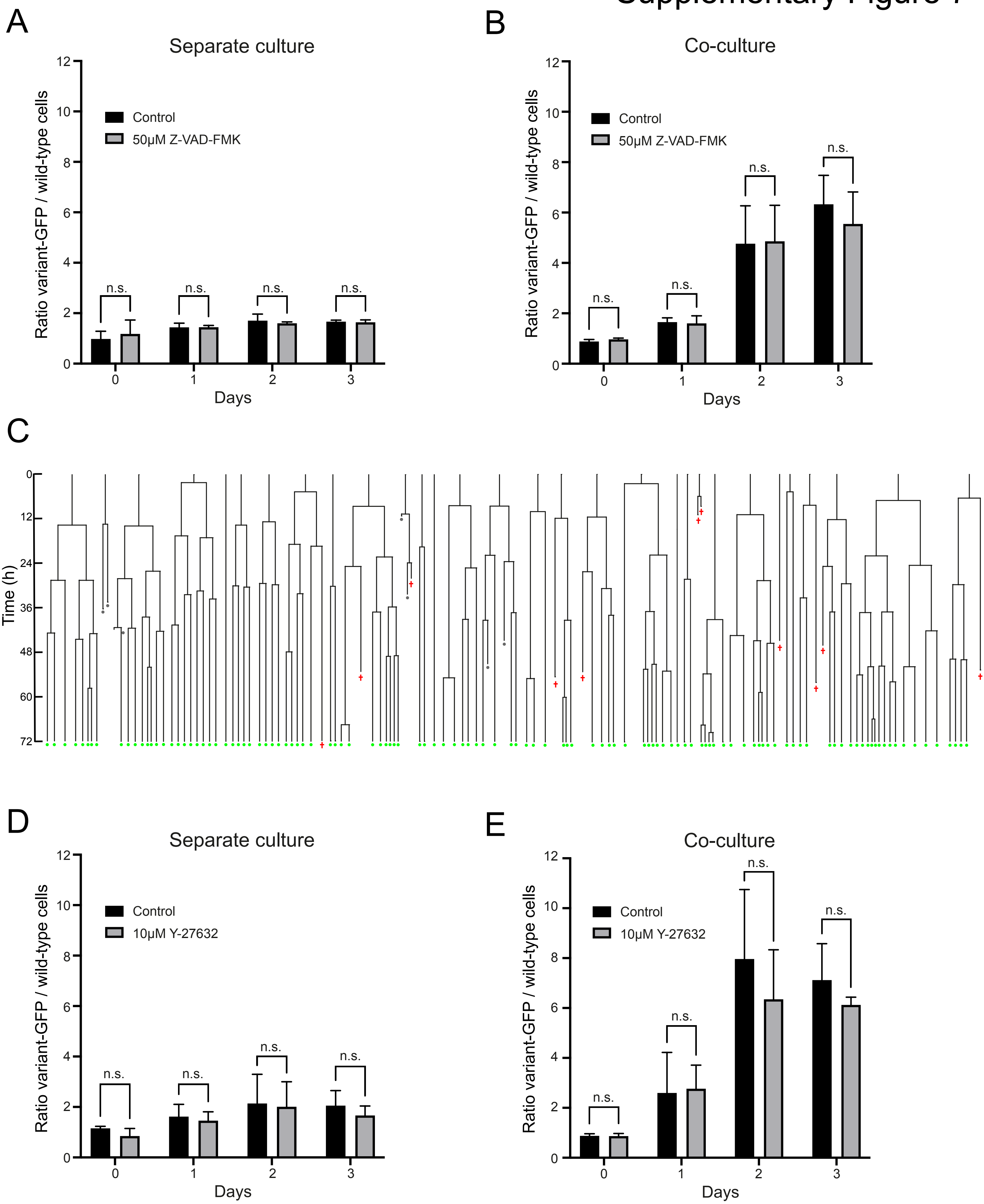

Figure S7. The common culture tools Z-VAD-FMK and Y-27632 do not restrict the competitive phenotype in hPSC cultures. Related to Figure 7.

A-B. The ratio of variant-GFP/wild-type cells grown in separate culture (A), and co-culture (B), in control or 50μM Z-VAD-FMK conditions.

C. Lineages trees of individual wild-type-RFP cells tracked from day 0 to day 3 of co-culture with variant-GFP treated with 50μM Z-VAD-FMK. Scale indicates time in hours. Green circles indicate cells that survived and grey circles indicate cells that could no longer be accurately tracked. Red crosses denote cell death. A total of 30 lineage trees is shown.

D-E. The ratio of variant-GFP/wild-type cells grown in separate culture (D), and co-culture (E), in control or 10μM Y-27632 conditions. Data are the mean of three independent experiments ± SD. n.s. non-significant; two-way ANOVA followed by Holm-Sidak's multiple comparisons test.
