# Supplementary material for: Genetically variant human pluripotent stem cells selectively eliminate wild-type counterparts through YAP-mediated cell competition
Source: Dev Cell. 2021 Sep 13;56(17):2455–2470.e10. doi: 10.1016/j.devcel.2021.07.019 (PMC8443275; doi:10.1016/j.devcel.2021.07.019)
Supplement: Document S2. Article plus supplemental information [file mmc9.pdf]

Developmental Cell

Article

Genetically variant human pluripotent stem cells selectively eliminate wild-type counterparts through YAP-mediated cell competition

Graphical abstract

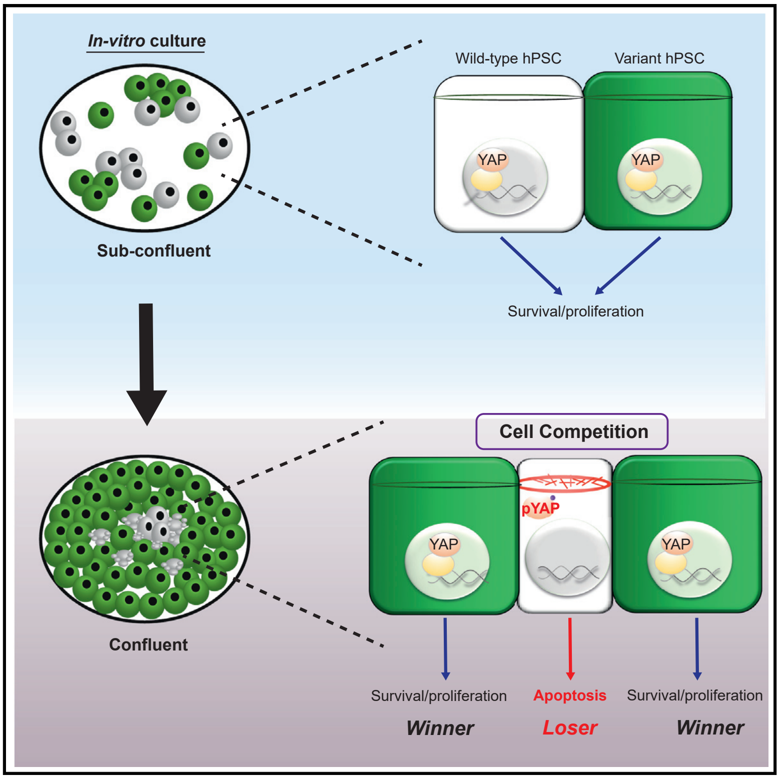

Authors

Christopher J. Price, Dylan Stavish, Paul J. Gokhale, ..., Joanne Lacey, Tristan A. Rodriguez, Ivana Barbaric

Correspondence

i.barbaric@sheffield.ac.uk

In brief

Price et al. identify competitive interactions that enhance selective advantage of genetically variant human pluripotent stem cells (hPSCs). In the presence of faster-growing variant hPSCs, wild-type cells sequester yes-associated protein (YAP) in the cytoplasm and undergo apoptosis. Neighboring variant cells retain nuclear YAP, remaining proliferative. Modifying culture conditions reduces competitive advantage of variant hPSCs.

Highlights

- Genetically variant human pluripotent stem cells (hPSCs) eliminate wild-type cells
- Mechanical cues mediate competitive interactions of wild-type and variant hPSCs
- Mechanotransducer YAP is a key mediator of cell competition in hPSC cultures
- Cell competition allows the fast overtake of cultures by variant hPSCs

Price et al., 2021, Developmental Cell *56*, 2455–2470
September 13, 2021 © 2021 The Author(s). Published by Elsevier Inc.
https://doi.org/10.1016/j.devcel.2021.07.019

CellPress

Developmental Cell

CellPress
OPEN ACCESS

Article

Genetically variant human pluripotent stem cells selectively eliminate wild-type counterparts through YAP-mediated cell competition

Christopher J. Price,[1,2] Dylan Stavish,[1,2] Paul J. Gokhale,[1] Ben A. Stevenson,[1] Samantha Sargeant,[1,3] Joanne Lacey,[1] Tristan A. Rodriguez,[4] and Ivana Barbaric[1,2,5,*]

[1]Department of Biomedical Science, University of Sheffield, Western Bank, Sheffield S10 2TN, UK
[2]Neuroscience Institute, University of Sheffield, Western Bank, Sheffield S10 2TN, UK
[3]Department of Automatic Control and Systems Engineering, University of Sheffield, Mappin Street, Sheffield S1 3JD, UK
[4]National Heart and Lung Institute, Imperial Centre for Translational and Experimental Medicine, Imperial College London, Hammersmith Hospital Campus, Du Cane Road, London W12 0NN, UK
[5]Lead contact
*Correspondence: i.barbaric@sheffield.ac.uk
https://doi.org/10.1016/j.devcel.2021.07.019

SUMMARY

The appearance of genetic changes in human pluripotent stem cells (hPSCs) presents a concern for their use in research and regenerative medicine. Variant hPSCs that harbor recurrent culture-acquired aneuploidies display growth advantages over wild-type diploid cells, but the mechanisms that yield a drift from predominantly wild-type to variant cell populations remain poorly understood. Here, we show that the dominance of variant clones in mosaic cultures is enhanced through competitive interactions that result in the elimination of wild-type cells. This elimination occurs through corralling and mechanical compression by faster-growing variants, causing a redistribution of F-actin and sequestration of yes-associated protein (YAP) in the cytoplasm that induces apoptosis in wild-type cells. YAP overexpression or promotion of YAP nuclear localization in wild-type cells alleviates their "loser" phenotype. Our results demonstrate that hPSC fate is coupled to mechanical cues imposed by neighboring cells and reveal that hijacking this mechanism allows variants to achieve clonal dominance in cultures.

INTRODUCTION

The ability of cells to influence their neighboring cells' fate choices has become apparent from studies in various *in vitro* and *in vivo* models. An example of this is cell competition, a type of cell-cell interaction wherein viable but less-fit "loser" cells are outcompeted for nutrients or space and eventually eliminated by the fitter "winner" cells (Bowling et al., 2019). Initially described and studied in *Drosophila* as a tissue-homeostatic mechanism (Morata and Ripoll, 1975), over recent years it has become evident that a form of cell competition, known as "super competition," is implicated in the expansion of cancerous cells (Eichenlaub et al., 2016; Suijkerbuijk et al., 2016). In super competition, the acquisition of a mutation which enhances the relative fitness of a cell results in the removal of neighboring wild-type cells (Johnston, 2014).

In the field of regenerative medicine, the fundamental question of how mutant cells may influence behavior of their wild-type counterparts has been brought into focus by observation that human pluripotent stem cells (hPSCs) acquire genetic changes upon prolonged passaging (Draper et al., 2004; International Stem Cell et al., 2011). Studies of the genetic integrity of hPSCs over the last two decades have revealed a bias in the genetic changes acquired in hPSCs, with the most common karyotypic abnormalities involving gains of chromosomes 1, 12, 17, 20, and X (Baker et al., 2016; Draper et al., 2004; International Stem Cell et al., 2011). The recurrent nature of genetic abnormalities in hPSCs is indicative of such changes conferring a selective growth advantage to the variant cells (Baker et al., 2007; Draper et al., 2004). The implications of the variant presence could be significant for therapeutic and research uses of hPSCs, as altered behavior of variant cells could impact on the efficiency of differentiation protocols, functionality of differentiated cells, or the safety of cell replacement therapies (Andrews et al., 2017; Halliwell et al., 2020).

The emergence of variant cells in hPSC cultures has been likened to the process of evolution, whereby the interplay of mutation and selection leads to the expansion of clones that possess the greatest growth advantage under particular culture conditions (Andrews et al., 2005). Indeed, selective advantage of commonly occurring genetic changes in hPSCs is evident from their increasing ratios in culture from the first point of detection over subsequent passages (Catalina et al., 2008; Draper et al., 2004; Imreh et al., 2006) and has also been demonstrated

This is an open access article under the CC BY license (http://creativecommons.org/licenses/by/4.0/).

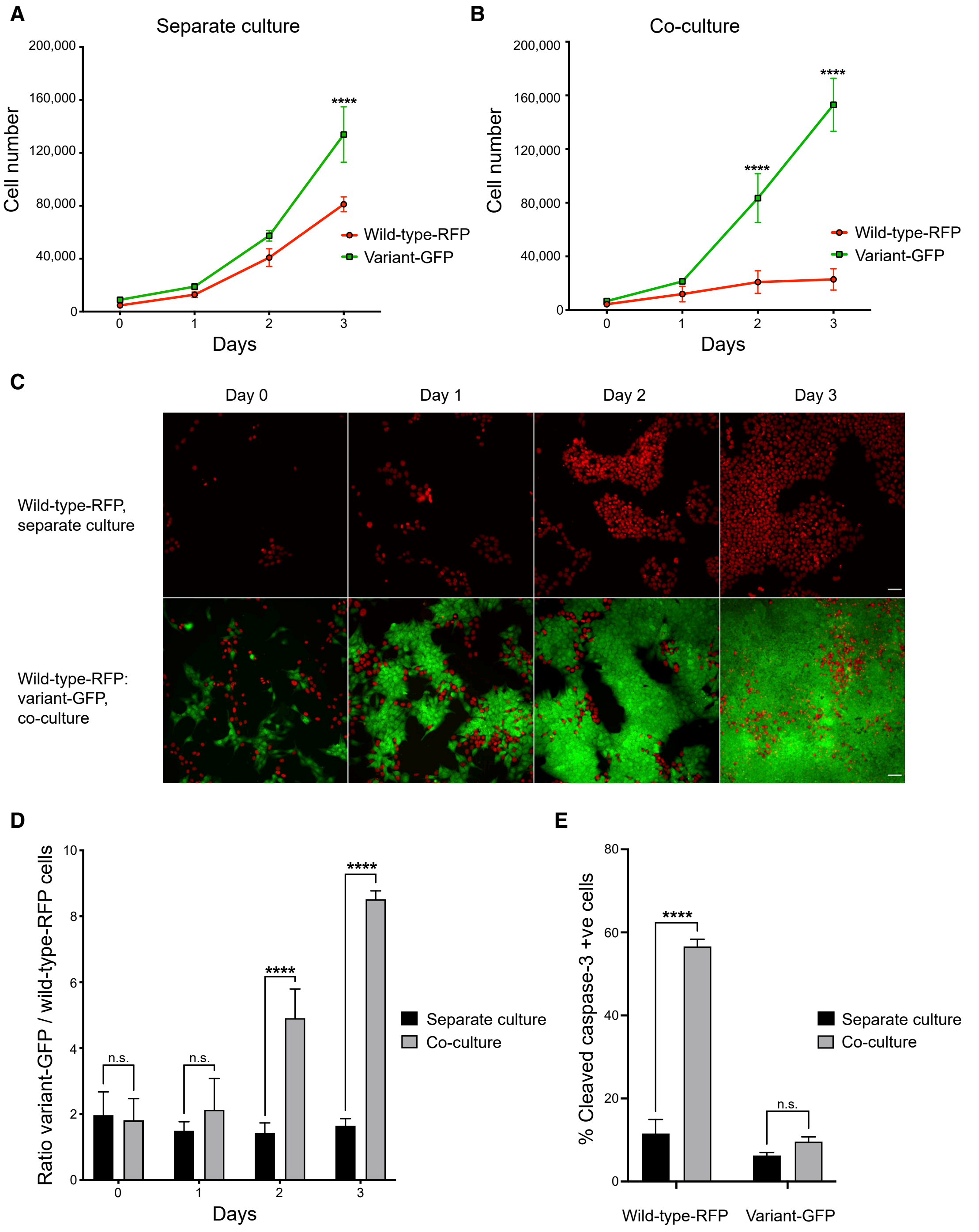

Figure 1. Wild-type cells are eliminated by apoptosis from co-cultures with variant hPSCs

(A–B) Growth curves of wild-type-RFP and variant-GFP cells grown separately (A), or in co-culture (B). Fields acquired: entire well.

(C) Representative images of wild-type-RFP hPSCs (red) grown separately (upper panels) or in co-culture with variant-GFP hPSCs (green) (lower panels). Scale bar: 50 μm.

(D) Ratio of variant-GFP/wild-type-RFP cells in separate versus co-culture conditions.

(E) Percentage of cells positive for cleaved caspase-3.

Data are the mean of three independent experiments ± SD. n.s. nonsignificant; ****p < 0.0001, two-way ANOVA, followed by Holm-Sidak's multiple comparisons test.

See also Figures S1, S2, and S3; Video S1.

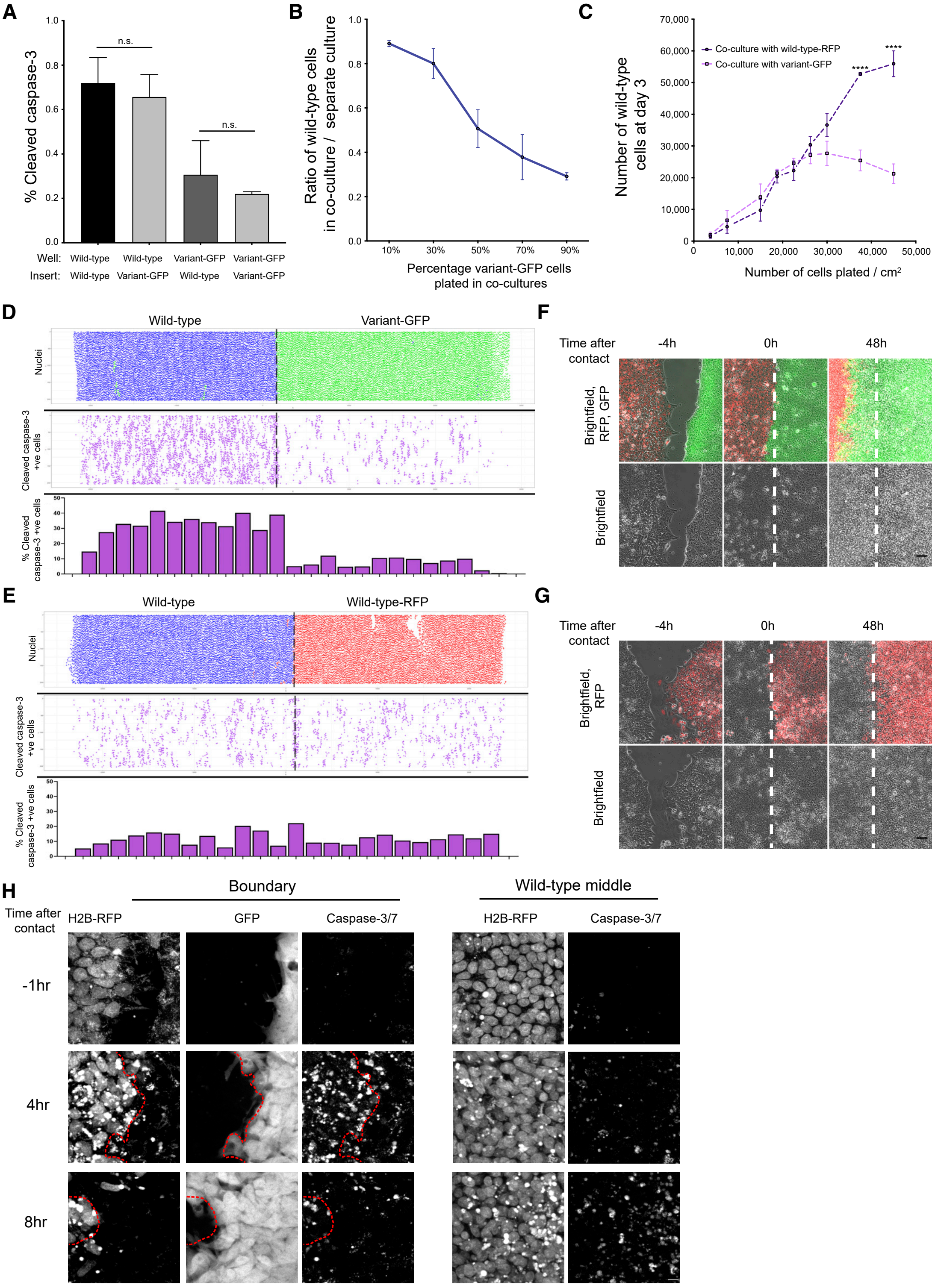

Figure 2. Cell competition in hPSC cultures is cell-contact mediated

(A) Percentage of caspase-3-positive cells in Transwell cultures of different sublines.

(B) Effect of increasing the ratio of variant-GFP cells in co-cultures with wild-type cells on the ratio of wild-type cells at day 3.

(C) Effect of increasing plating cell density of co-cultures on the number of wild-type cells.

(D and E) Cell-confrontation assay of wild-type-RFP and variant-GFP cells (D) and wild-type and wild-type-RFP (E) cells at 48 h post contact. (D) Top panel: nuclei of wild-type-RFP and variant-GFP cells represented as blue and green dots, respectively. Top panel (E): nuclei of wild-type and wild-type-RFP cells represented as blue and red dots, respectively. Middle panel (D and E), cleaved caspase-3-positive cells represented as purple dots. Bottom panels: Percentage of cleaved

(legend continued on next page)

through mixing experiments, wherein spiking a small proportion of variant cells into wild-type cultures resulted in a rapid overtake of cultures by the variants (Avery et al., 2013; Olariu et al., 2010). To explain the reasons behind the variants' overtake of cultures, studies of variant hPSCs have mostly focused on the intrinsic properties that could lead to their growth advantage, such as enhanced proliferation and reduced levels of apoptosis (Avery et al., 2013; Barbaric et al., 2014; Ben-David et al., 2014; Draper et al., 2004; Enver et al., 2005; Nguyen et al., 2014). Yet, when variant cells first emerge, they coexist within the same culture as the wild-type cells, and hence share their culture environment as well as a proportion of their cell-cell contacts. However, little is known about the nature of wild-type and variant hPSC cell-cell interactions and whether the presence of variants affects the growth and survival of wild-type hPSCs.

Here, we show that an important aspect of the selective advantage displayed by some commonly occurring variant hPSCs is the ability to induce apoptosis of wild-type cells in mosaic cultures, akin to the super-competition-like behavior described in other cell types (de la Cova et al., 2004; Moreno and Basler, 2004). The elimination of loser cells in hPSC cultures is exerted through mechanical cues and is mediated by yes-associated protein (YAP), downstream of the actomyosin cytoskeleton. Our findings illuminate the reliance of hPSC fates on their mechanical environment and highlight the need for consideration of mechanical cues in the scale up of hPSCs for research or clinical use.

RESULTS

Variant hPSCs selectively eliminate diploid wild-type counterparts from co-cultures

To uncover the reasons behind the rapid overtake of cultures by genetically variant hPSCs (Olariu et al., 2010), we sought to examine how wild-type and genetically variant hPSCs interact and whether they affect each other's growth. To this end, we initially used two diploid H7 sublines (either non-modified or genetically engineered to constitutively express red fluorescent protein [RFP], termed wild-type and wild-type-RFP, respectively), and their aneuploid variant harboring a gain of chromosomes 1, 12, 17q, and 20q copy number variant (CNV), and stably expressing green fluorescent protein (GFP) (termed variant-GFP). Time-lapse microscopy of co-cultures containing wild-type-RFP and variant-GFP cells showed a selective elimination of wild-type-RFP cells during a 3-day culture period (Video S1). To establish that the observed elimination is due to the presence of variant cells in mixed cultures we compared the growth rates of wild-type-RFP or unlabeled wild-type cells in separate cultures with how they grew in mixed cultures with variant-GFP cells. Wild-type sublines were viable and created well-established, large colonies in separate cultures but, consistent with previous findings, did grow more slowly than variant cells (Figures 1A and S1A) (Barbaric et al., 2014; Enver et al., 2005). In striking contrast to this, upon mixing with equal numbers of variant-GFP cells, the wild-type-RFP or unlabeled wild-type cells showed severely compromised growth (Figures 1B, 1D, and S1A). The co-culture had no effect on the number of variant-GFP cells (Figures 1A, 1B, and S1A). We observed a similar decrease of wild-type-RFP cell numbers upon mixing them with further aneuploid H7 sublines harboring recurrent genetic changes, such as a variant subline with a gain of chromosome 17q (termed *v17q*) (Figure S1B) and a subline with gains of chromosomes 1 and 17q and isochromosome 20q (termed *v1,17q,i20*) (Figure S1C). Moreover, we detected competitive behavior in additional pairs of diploid and aneuploid cells from different hPSC lines (H14 and HUES-17), with aneuploid cells (termed H14 variant-BJ1 and HUES-17*v12,17*) also outcompeting diploid cells in co-cultures (Figures S1D and S1E). Conversely, we detected no competitive behavior when mixing wild-type cells and fluorescently labeled sublines from the same line (H7, H14, and HUES-17), with the initial mixing ratio of wild-type sublines retained both in short-term culture (over 3 days) (Figures S2A–S2C) and upon longer-term passaging (over 5 passages) (Figures S2D–S2F). Overall, these experiments demonstrated that the presence of variant cells negatively affects the numbers of wild-type hPSCs in co-cultures with variants.

The elimination of wild-type hPSCs which occurred in co-culture with variants is reminiscent of cell competition described in many different systems, whereby "weaker," *loser* cells are eliminated in the presence of "fitter" *winner* cells (Bowling et al., 2019). Cell competition typically involves inducing either senescence (Bondar and Medzhitov, 2010) or apoptosis (Brumby and Richardson, 2003; Moreno et al., 2002; Sancho et al., 2013) in loser cells. From our time-lapse analysis of wild-type-RFP cells co-cultured with either variant-GFP cells or unlabeled wild-type cells as a control, it was evident that the loser cells were not arresting in co-cultures (Figure S3). On the other hand, using cleaved caspase-3 staining as a readout of apoptosis, we observed that while wild-type and variant cells showed similar levels of cell death in separate cultures, the proportion of apoptotic cells was significantly increased in wild-type cells upon co-culture with variants (Figures 1E and S1F–S1J). There was no change in the cleaved caspase-3 levels of variant cells upon co-culture with wild-type hPSCs (Figures 1E and S1F–S1J). Based on these results, we concluded that the presence

caspase-3-positive cells calculated as the number of cleaved caspase-3-positive cells in the total cell number within a defined area of a cell insert. The width of the bar corresponds to the analyzed area of the insert shown in the middle panel above.

(F and G) Time-lapse images of cell-confrontation assay of wild-type-RFP (red) and variant-GFP (green) cells (F) or wild-type and wild-type-RFP (red) cells (G). Left panel: inserts at 4 h before contact. Middle panel: inserts at the time when cells first come into contact (denoted as 0 h). Right panel: inserts at 48 h post contact. Dashed white line indicates the position on the insert where the two different populations first met at 0 h.

(H) Time-lapse images of cell-confrontation assay of wild-type and variant-GFP cells with live caspase-3/7 dye. Left panel: region of the inserts where wild-type and variant-GFP cells come into contact. Dashed red line indicates the boundary between the two different populations. Right panel: middle region of the wild-type cell population on the inserts. Scale bar: 10 μm.

Data represent the mean of three independent experiments ± SD (A–C). Statistical analysis was performed by Student's t test (A) or two-way ANOVA, followed by Holm-Sidak's multiple comparisons test (B); n.s. nonsignificant; ****$p < 0.0001$.

See also Figure S4; Videos S2 and S3.

CellPress
OPEN ACCESS

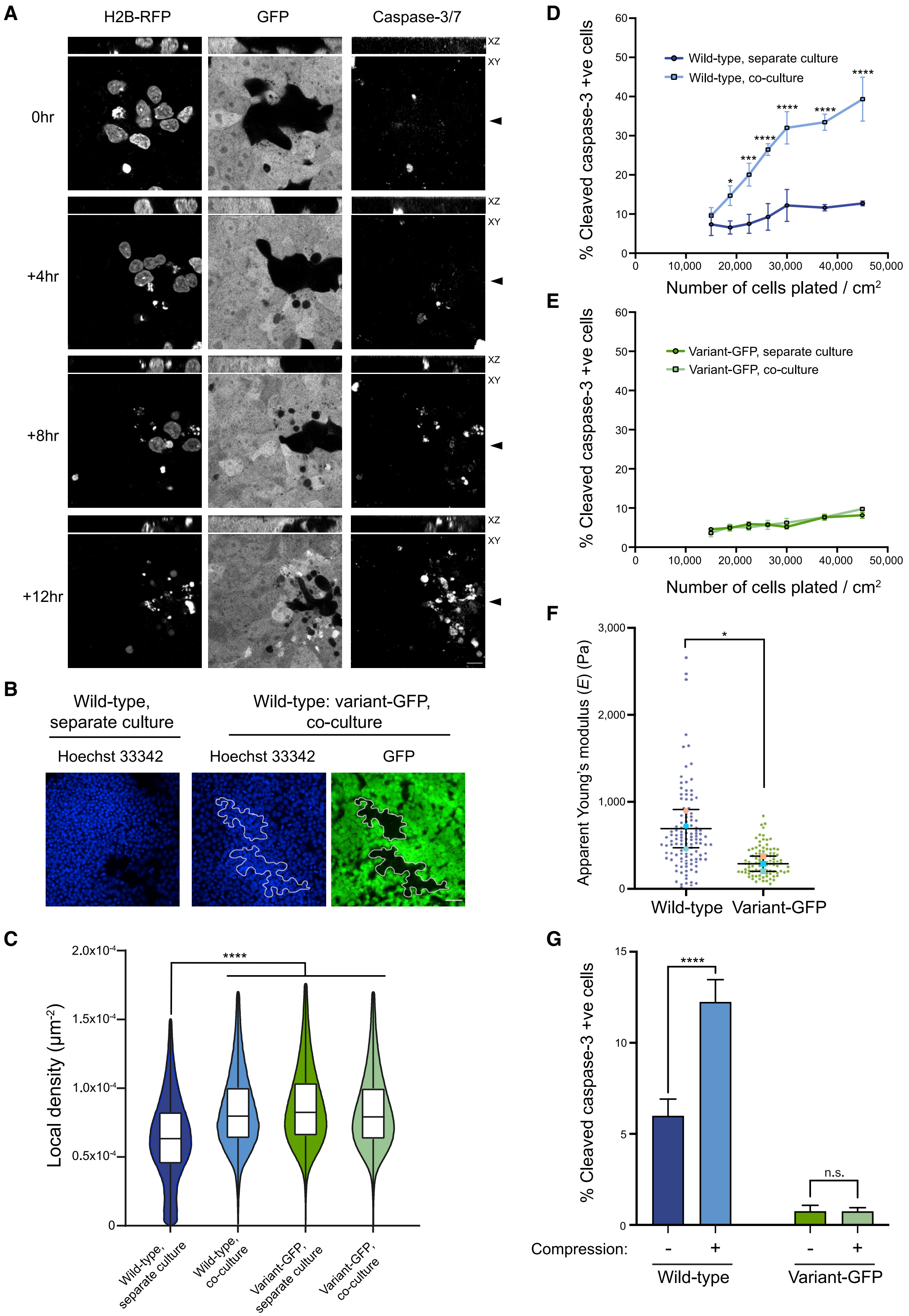

(legend on next page)

of variant cells is inducing apoptosis and thereby the elimination of wild-type cells from mosaic cultures.

Crowding of loser cells within mosaic cultures induces loser-cell apoptosis

Given the apparent selective elimination of wild-type cells when co-cultured with variant hPSCs, we wanted to establish whether the increased death rate of wild-type cells was mediated through cell-cell contacts or by cell-secreted diffusible factors, or a combination of both. To this end, we performed conditioned-medium experiments, whereby wild-type and variant hPSCs were grown in a medium conditioned by the same or competing cell type. We found no increase in the activated caspase-3 levels in wild-type cells upon incubation with medium conditioned by their variant counterparts (Figures S4A–S4C). We also made use of a Transwell assay (Boyden, 1962) to spatially separate wild-type and variant populations while allowing the free exchange of secreted factors in the culture media. In these conditions, the presence of variant-GFP cells did not increase the levels of activated caspase-3 staining in wild-type cells compared with wild-type cells when co-cultured with wild-type cells in Transwell cultures (Figure 2A), indicating that the effect of variants on wild-type cells is not mediated by soluble factors. In contrast to this, plating the increasing ratios of variant-GFP cells in co-cultures (from 10% to 90%) in a monolayer caused an increasing suppression of wild-type cell numbers (Figure 2B) in a cell-density-dependent manner (Figure 2C). Together, these results demonstrate that cell competition in hPSC cultures is mediated by cell contact rather than by soluble factors.

The increased loss of wild-type cells upon increasing the ratio of variant cells in co-cultures, or upon plating the co-cultures at increasing cell densities, could be explained by one of two possibilities: either the higher numbers of wild-type-variant heterotypic cell contacts result in receptor-mediated cell competition (Burke and Basler, 1996); or alternatively, the winner cells are mechanically compressing the losers, causing their eradication from cultures in a process termed mechanical cell competition (Levayer et al., 2016; Wagstaff et al., 2016). To distinguish between these possibilities, we first performed a cell-confrontation assay, which allows two cell populations to be brought into contact at a clearly defined border (Moitrier et al., 2019; Porazinski et al., 2016). We reasoned that the receptor-mediated competition would result in cell apoptosis localized at the border of heterotypic cell contacts, whereas mechanical cell competition would result in the apoptotic signal spread throughout the areas of cell crowding (Brás-Pereira and Moreno, 2018). We plated wild-type-RFP and variant-GFP cells within separate chambers of a commercially available culture insert and allowed them to populate the area within their respective chambers overnight. Upon removal of the insert, the cells from different chambers were allowed to come into contact with each other and were then cultured for a further 48 h, prior to fixing and staining for the apoptotic marker cleaved caspase-3. Supporting the notion that cell competition in hPSC cultures is mediated through mechanical means, we found that the cleaved caspase-3 was distributed within the wild-type-RFP cells beyond the heterotypic border with variant-GFP cells (Figure 2D). This effect was specifically caused by the presence of variants in the cell-confrontation assay, as inserts containing wild-type-RFP cells and unlabeled wild-type counterparts resulted in less caspase-3 staining compared with wild-type-RFP: variant-GFP confrontation cultures (Figure 2E). Moreover, time-lapse imaging of the cell fronts from the time of contact over the subsequent 52 h also revealed that the wild-type-RFP hPSCs were pushed back by the advancing variant-GFP population (Figure 2F; Video S2), whereas the wild-type: wild-type-RFP cells boundary remained in a similar position over 52 h of tracking (Figure 2G; Video S3). The advancement of the variant-GFP population was accompanied by an increased activation of caspase in the wild-type-RFP population. Apoptotic signaling was initially evident in the area close to the boundary between the two populations and subsequently in regions of wild-type-RFP population more distant from the boundary, which were becoming crowded as a result of the variants advancing and invading their space (Figures 2H and S4D). Together, these results suggest that the variant-GFP cells outcompete the wild-type cells in the competition for space.

The competition for space that we detected in the cell-confrontation assays was also evident in mosaic co-cultures grown in a monolayer. We tracked cells by time-lapse microscopy in the presence of live caspase staining, and we noted an apparent corralling of wild-type-RFP by variant-GFP cells, followed by caspase activation in wild-types and their subsequent extrusion from the monolayer (Figure 3A; Video S4). We confirmed the corralling effect by analyzing the relative cell density of wild-type and variant-GFP cells in separate cultures and upon co-culture. The nuclei of wild-type cells in co-cultures

Figure 3. Wild-type cells are corralled by variants into areas of high cell density

(A) Time-lapse images of wild-type-RFP and variant-GFP cells in co-culture from day 2 in the presence of live caspase-3/7. Closed arrowheads denote the position of the z axis within the x-y plane. Scale bar: 10 μm.

(B) Corralling of wild-type cells by variant-GFP counterparts. The outlined areas in the middle and right panels indicate regions of co-culture harboring wild-type cells. Scale bar: 50 μm.

(C) Cell density of wild-type and variant-GFP cells grown either separately or in co-cultures.

(D and E) Percentage of wild-type (D) and variant-GFP (E) cells positive for cleaved caspase-3 grown either in separate culture or upon co-culture at increasing plating densities.

(F) Apparent Young's modulus (E) of wild-type and variant-GFP cells. Small points indicate individual cells and larger circles indicate a mean from each of the three independent experiments.

(G) Cell-compaction assay; percentage of wild-type and variant-GFP cells positive for cleaved caspase-3 indicator of apoptosis on uncompressed or compressed membranes (denoted as - and + compression, respectively). Data are the mean of three independent experiments ± SD. ****$p < 0.0001$, one-way ANOVA. Data represent the values of individual cells (C) or the mean (D–G) from three independent experiments (C–G) ± SD. Statistical analysis was performed by one-way ANOVA (C and G); two-way ANOVA, followed by Holm-Sidak's multiple comparisons test (D and E) or Student's t test (F); n.s., nonsignificant; *$p < 0.05$, ***$p < 0.001$, ****$p < 0.0001$.

See also Figure S4; Video S4.

CellPress
OPEN ACCESS

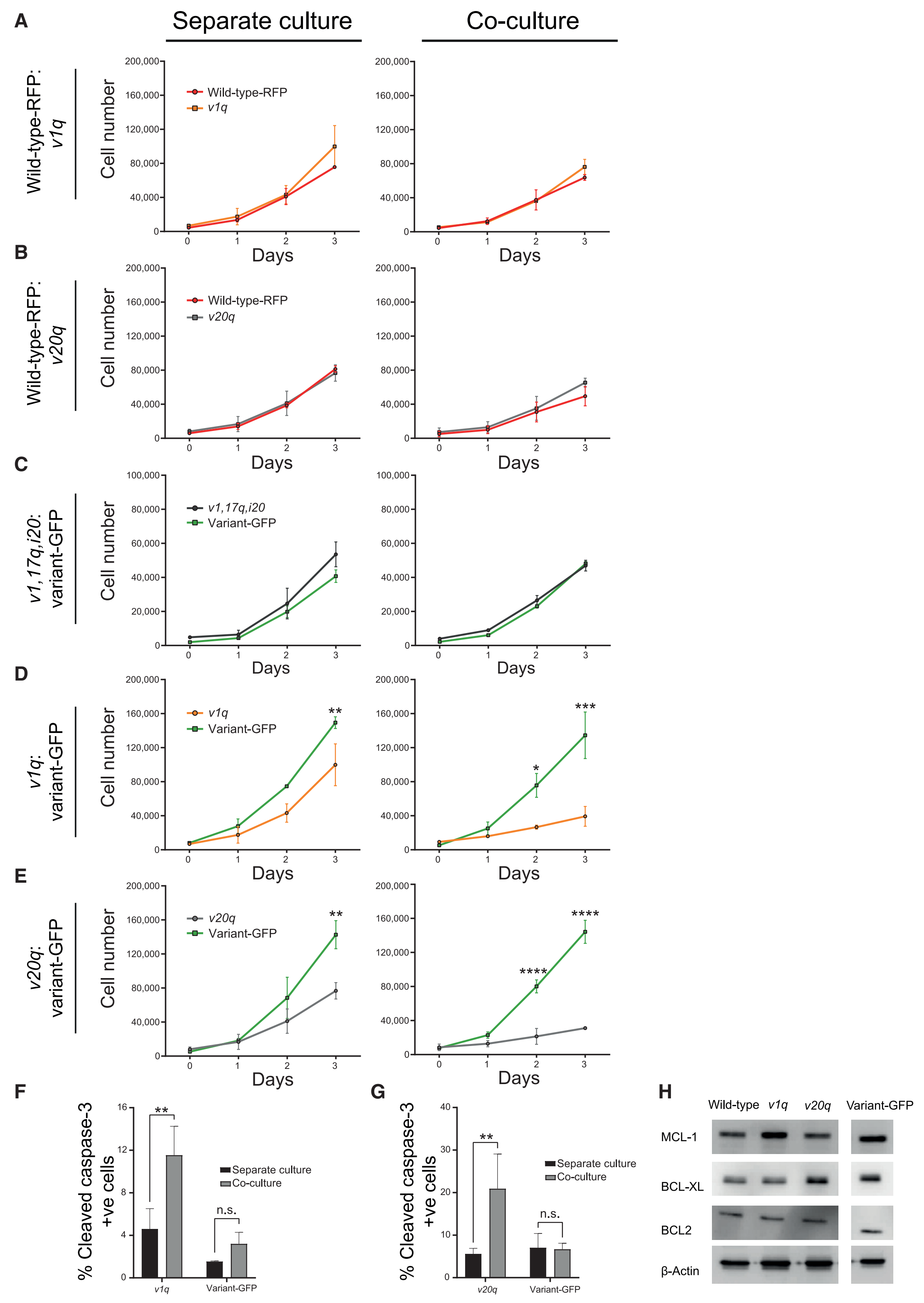

Figure 4. The winner phenotype is dependent on higher proliferative rates of variant cells

(A–E) Growth curves of wild-type-RFP and *v1q* cells (A), wild-type-RFP and *v20q* cells (B), V1,*17q,i20* and variant-GFP cells (C), *v1q* and variant-GFP cells (D), and *v20q* and variant-GFP cells (E) grown separately or in co-culture. Cells in (A, B, D, and E) were plated at 45,000 cells/cm^2. Cells in (C) were plated at the lower

(legend continued on next page)

clustered within areas of increased local density compared with the density within the separate wild-type culture (Figures 3B and 3C). The differential response of wild-type and variant cells to crowding conditions was evident from increased levels of cleaved caspase-3-positive cells which occurred at increasing cell densities of wild-type cells but not the variant ones (Figures 3D, 3E, and S4E). Moreover, compared with their wild-type counterparts, variant cells in separate cultures exhibited reduced cellular stiffness (the apparent Young's modulus) (Figures 3F and S4F), suggesting that differences in their mechanical properties could be underpinning a differential response to cell crowding.

To directly assess the sensitivity of wild-type and variant-GFP cells to compaction-mediated cell death induced by cell-crowding conditions, we used a compaction assay (Wagstaff et al., 2016). This entailed plating wild-type and variant-GFP cells on an elastic substrate stretched by 35% and then releasing the stretch to compress the substrate, thereby inducing an increase in cell density. We noted a 2-fold increase in activated caspase-3 levels in wild-type cells on the compressed substrate over their uncompressed counterparts (Figure 3G). Conversely, we detected no increase in cleaved caspase-3 staining upon compression of variant-GFP cells. Collectively, these results show that the corralling of wild-type hPSCs by variants within mosaic cultures causes them to crowd into areas of high local-cell density and thereafter commit to apoptosis.

Winner status is conferred onto cells by having a relatively higher proliferative ability

Given that a key feature of mechanical cell competition is the crowding of loser cells caused by the faster-growing winners (Levayer et al., 2016; Wagstaff et al., 2016), we next asked whether the winner status in hPSC cultures is conferred onto cells by the ability to expand faster and thus fill the available space. To this end, we performed mixing experiments of H7 wild-type-RFP cells with a range of H7 variant sublines, which harbored distinct genetic changes and displayed diverse growth rates. For example, variant H7 sublines with a gain of 1q (hereafter, *v1q*) or a gain of 20q copy-number variant (*v20q*) had similar growth rates to wild-type-RFP hPSCs in separate cultures (Figures 4A and 4B). As predicted, upon mixing with wild-type cells, the numbers of wild-type or variant cells (either *v1q* or *v20q*) remained unaffected (Figures 4A and 4B). In addition, we tested the behavior of *v1,17q,i20* variant lines in co-culture with variant-GFP cells, as both lines grew at equivalent rates when cultured separately (Figure 4C). Again, the growth rate profiles of each of these variants were unaffected by their co-culture (Figure 4C), demonstrating that no competition takes place in cultures of cells with equivalent growth rates. Conversely, culturing variant lines *v1q* and *v20q* separately or in co-culture with the faster-growing variant-GFP cells showed a significantly decreased number of *v1q* and *v20q* cells within co-cultures compared with separate cultures (Figures 4D and 4E). We confirmed that the decrease of *v1q* and *v20q* cell numbers in co-cultures with variant-GFP cells was due to apoptosis, as both sublines showed higher levels of cleaved caspase-3 staining in the co-culture condition compared to separate culture (Figures 4F and 4G). Nonetheless, neither the decrease in cell numbers nor the level of activated caspase-3 in *v1q* and *v20q* upon competition with variant-GFP was as extensive as seen in wild-type cells upon mixing with variant-GFP. The *v20q* and *v1q* variant lines harbor an additional copy of *BCL2L1* and *MCL-1*, respectively. The higher levels of expression of antiapoptotic proteins BCL-XL and MCL-1 (Figure 4H) are thought to confer *v20q* and *v1q* cells with increased resistance to apoptosis (Avery et al., 2013; Nguyen et al., 2014). Given that *v1q* and *v20q* variants did not assume a winner status upon mixing with wild-type cells, our data revealed that increased resistance to apoptosis is not sufficient to confer a winner-cell phenotype. Together, these results confirmed that cell competition behavior is context dependent and that the faster proliferation rate and higher homeostatic density are features of variant hPSCs exhibiting a winner-cell phenotype. Moreover, we show that increased resistance to apoptosis is not sufficient to confer cells with a winner-cell phenotype, but it reduces the rate of loser-cell elimination.

YAP mediates the winner versus loser-cell phenotype in hPSCs

To determine how cell competition in hPSC cultures is mediated at the molecular level, we initially performed transcription analysis of loser (*v1q*) and winner (variant-GFP) cells in both separate and co-cultures (Figures S5A and S5B). The use of v*1q* rather than wild-type cells facilitated the capture of sufficient numbers of cells in sorting experiments due to their increased resistance to apoptosis. We first focused on identifying expression signatures associated with prospective winner and loser populations, by analyzing the differential gene expression between these cells in separate cultures (Figures S5C and S5D). In line with the complex aneuploidy of variant-GFP cells, the number of differentially expressed genes in winner versus loser cells was large, with 3,524 genes significantly upregulated and 3,311 genes significantly downregulated in winner cells compared with loser cells (Figure S5D). The Kyoto Encyclopedia of Genes and Genomes (KEGG) enrichment analysis (Kanehisa et al., 2010) showed that the most significantly enriched molecular network from the downregulated genes was the ribosomal pathway, followed by the cell cycle and TGF-β signaling (Figure S5E; Table S1). The Hippo signaling was the only significantly enriched pathway in the KEGG analysis of differentially expressed genes between winner and loser cells upon co-culture (Figures S5F and S5G; Table S2). Thus, we next focused on yes-associated protein 1

density of 22,500 cells/cm^2 due to the faster growth rate of both sublines with a complex karyotype. Fields acquired: entire well. Data represent the mean of two independent experiments (A, B, D, and E) or the mean of 6 technical replicates from the same experiment (C) ± SD.

(F–G) Percentage of cells positive for cleaved caspase-3 in *v1q* and variant-GFP cells (F) and *v20q* and variant-GFP cells (G) in separate cultures or upon co-culture. Data are the mean of three independent experiments ± SD.

(H) Western blot of antiapoptotic proteins in wild-type, *v1q*, *v20q*, and variant-GFP cells. β-actin was used as a loading control.

Statistical analysis was performed by two-way ANOVA, followed by Holm-Sidak's multiple comparisons test (A, B, D, E, F, and G) or Student's t test (C); n.s. nonsignificant; *p < 0.05, **p < 0.01, ***p < 0.001, ****p < 0.0001.

CellPress
OPEN ACCESS

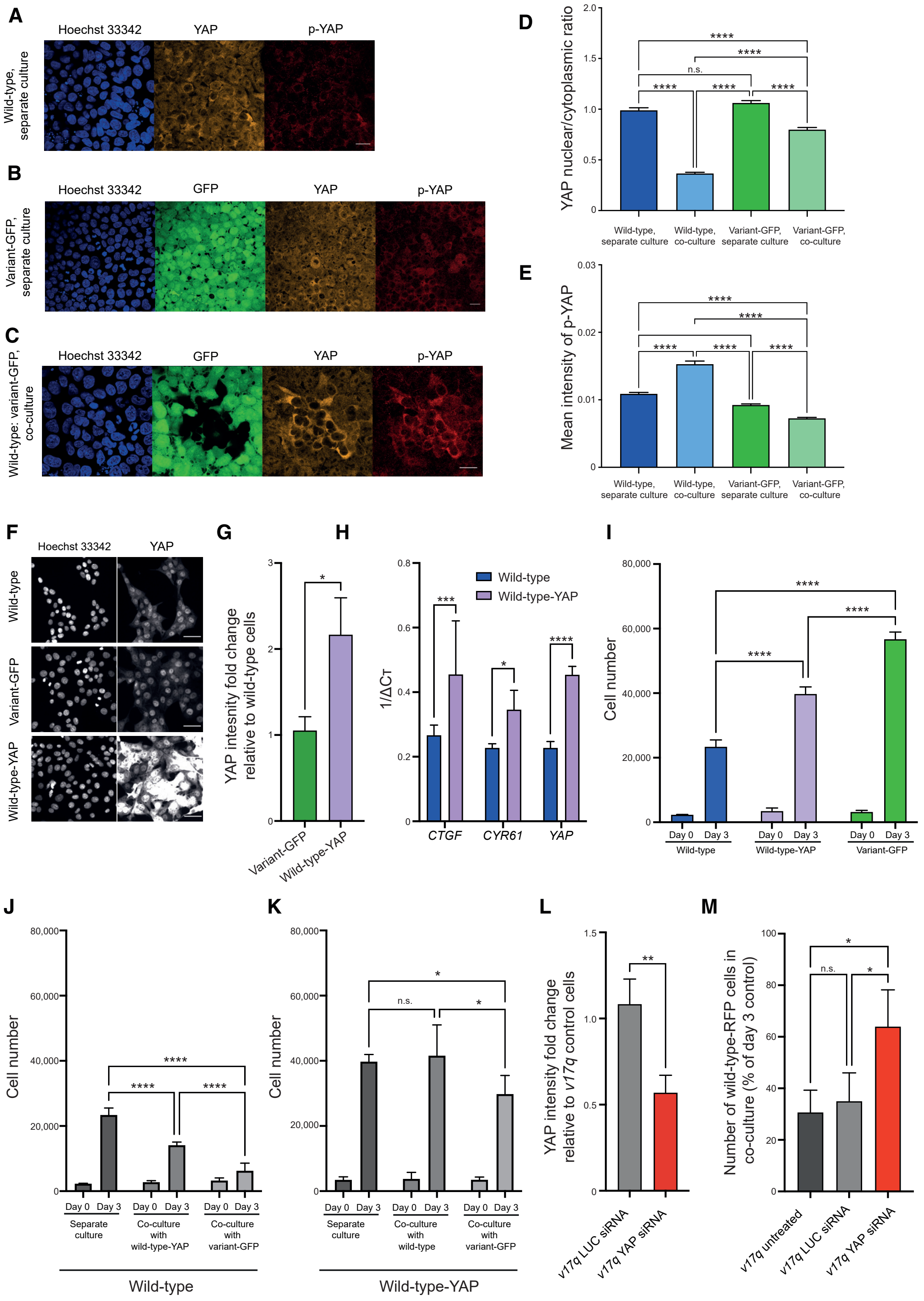

Figure 5. YAP mediates the winner versus loser hPSC phenotype

(A–C) YAP displays cytoplasmic localization in crowded wild-type cells co-cultured with variants. YAP (orange) and phosphorylated YAP (p-YAP) (red) staining in wild-type cells in separate culture (A), variant-GFP cells in separate culture (B) and upon co-culture (C). Scale bar: 25 μm.

(D–E) Nuclear to cytoplasmic ratio of YAP (D), and the mean intensity of phosphorylated YAP in the cytoplasm (E). Data are represented as the mean ± SEM.

(F) Representative images of YAP staining in separate cultures. Scale bar: 50 μm.

(G) YAP intensity in variant-GFP and wild-type YAP-overexpressing cells relative to the wild-type cells.

(legend continued on next page)

(YAP1, also known as YAP), as a major effector of the Hippo signaling. YAP was previously shown to be also modulated by mechanical signaling, including mechanical stresses imposed by neighboring cells (reviewed in (Panciera et al., 2017)) and was implicated in cell competition in different model systems (Hashimoto and Sasaki, 2019; Liu et al., 2019; Moya et al., 2019; Neto-Silva et al., 2010; Ziosi et al., 2010).

We first checked YAP localization in separate and mosaic cultures of wild-type and variant-GFP cells by immunofluorescence. YAP localized predominantly to the nucleus of both wild-type and variant-GFP cells when they were grown in separate cultures (Figures 5A, 5B, and 5D). Strikingly, while the variant-GFP cells retained the nuclear YAP in co-cultures with wild-type cells, the wild-type cells within the same culture exhibited a shift in YAP localization from nuclear to cytoplasmic (Figures 5C, 5D, S5H, and S5I). The cytoplasmic YAP in wild-type cells was phosphorylated at Ser127 residue (Figures 5C and 5E), indicating that co-culture with variant cells leads to sequestration and inactivation of YAP in the cytoplasm of wild-type hPSCs.

To directly address the hypothesis that YAP is mediating the super competition behavior of hPSCs, we first overexpressed YAP in wild-type cells (Figures 5F–5H, S6A, and S6B) and analyzed the effect of overexpression on the cell phenotype. YAP overexpression was previously reported to promote the naive state of hPSCs transferred to naive culture conditions (Qin et al., 2016). Under the “primed” conditions used in our study, YAP overexpression did not affect the expression of markers associated with undifferentiated, primed, or naive states of pluripotency (Figures S6C and S6D). However, YAP overexpression resulted in the improved growth rates and increased homeostatic density of wild-type cells (Figures 5I and S6E). Further, YAP-overexpressing cells exhibited a winner phenotype in co-cultures with wild-type cells (Figures 5J and S6F). Finally, in comparison with wild-type cells, YAP-overexpressing cells were more resistant to crowding caused by co-culture with variant-GFP cells, as evidenced by higher numbers of YAP-overexpressing cells persisting in co-cultures with variant-GFP cells (Figures 5K and S6G). Conversely, the knockdown of YAP in variant cells suppressed their winner phenotype upon co-culture with wild-type cells (Figures 5L, 5M, S6H, and S6I). Based on these results, we concluded that YAP is a major contributor to cell competition in hPSC cultures.

Apical actin constriction regulates YAP localization in hPSCs

To gain further mechanistic insight into YAP-mediated hPSC competition, we set out to investigate the upstream regulators of YAP in this context. Our observation that wild-type hPSCs are corralled into smaller spaces upon co-culture with variants, coupled with the findings from other cell models that YAP localization can be mechanically influenced by cell shape and actin fibers (Aragona et al., 2013; Wada et al., 2011), prompted us to examine the cytoskeleton as a potential regulator of YAP in hPSCs. Phalloidin staining of F-actin showed a similar basal-to-apical profile of actin fibers in wild-type and variant-GFP cells in separate cultures, with both populations exhibiting a faint staining of actin filaments encircling the cell within the adhesion belt (Figure 6A). However, while the variant cells retained a similar actin distribution upon co-culture with wild-type cells, the crowded wild-type cells showed a dramatic change in their actin fiber network (Figure 6B). Specifically, we detected a redistribution of actin stress fibers within the adhesion belt, evident as intense staining of F-actin within the circumferential actin ring (Figure 6B). Expression of myosin IIB, a major nonmuscle myosin, was also upregulated in the adhesion belt of the crowded wild-type cells (Figures 6C and 6D), reflecting the increased constriction of the adhesion belt in these cells upon co-culture with variants.

To determine whether the observed cytoskeletal differences in winner and loser cells upon co-culture underpin the differences in their subcellular YAP localization, we utilized a set of chemicals that perturb actomyosin cytoskeleton. First, we used nocodazole to disrupt microtubules. Microtubule disruption, evident by diminished α-tubulin staining (Figures 6E and 6F), reduced the adhesion belt contraction in crowded wild-type cells (Figure 6G). Concomitantly, we detected a shift from a predominantly cytoplasmic YAP in co-cultured wild-type cells to a diffuse (i.e., both cytoplasmic and nuclear) localization in their nocodazole-treated counterparts (Figure 6H). Disruption of actin fibers using latrunculin A or cytochalasin B also resulted in reduced actin ring within the adhesion belt of crowded wild-type cells and a diffuse localization of YAP in those cells (Figures 6I–6M). On the other hand, inhibition of myosin activity by treating cells with the rho-associated coiled coil kinase (ROCK) inhibitor changed the actin stress fibers at the cell-extracellular matrix level but did not reduce the intense actin staining within the adhesion belt of the crowded wild-type cells (Figures 6N and 6O), and ROCK inhibitor had no overt effect on the subcellular localization of YAP (Figure 6P). Taking these findings together, we conclude that in hPSC cultures super-competitive variant cells corral wild-type counterparts into areas of significantly higher density compared with the density of wild-type separate cultures. Consequent restructuring of actin fibers within the adhesion belt of crowded wild-type hPSCs causes sequestering

(H) YAP and its target genes are upregulated in wild-type YAP-overexpressing cells compared with wild-type hPSCs.
(I) YAP overexpression leads to improved growth of wild-type cells.
(J and K) YAP overexpression confers the winner phenotype in co-cultures with wild-type cells (J) and increased resistance to cell crowding in co-cultures with variant-GFP cells (K).
(L) YAP intensity in *v17q* cells transfected with either LUC or YAP siRNA relative to *v17q* control cells.
(M) Knockdown of YAP in *v17q* cells co-cultured with wild-type-RFP cells partially rescues the loser phenotype in wild-type-RFP hPSCs.
(G–M) Data are the mean of three independent experiments ± SD. (I, J, K, M), 16 fields were acquired per well.
Statistical analysis was performed by one-way ANOVA followed by Kruskal-Wallis multiple comparisons test (E), Student’s t test (G and L), one-way ANOVA followed by Fisher’s LSD test (H), or two-way ANOVA followed by Holm-Sidak’s multiple comparisons test (I, J, K, M); n.s. nonsignificant; *$p < 0.05$, **$p < 0.01$, ***$p < 0.001$, ****$p < 0.0001$.
See also Figures S5 and S6.

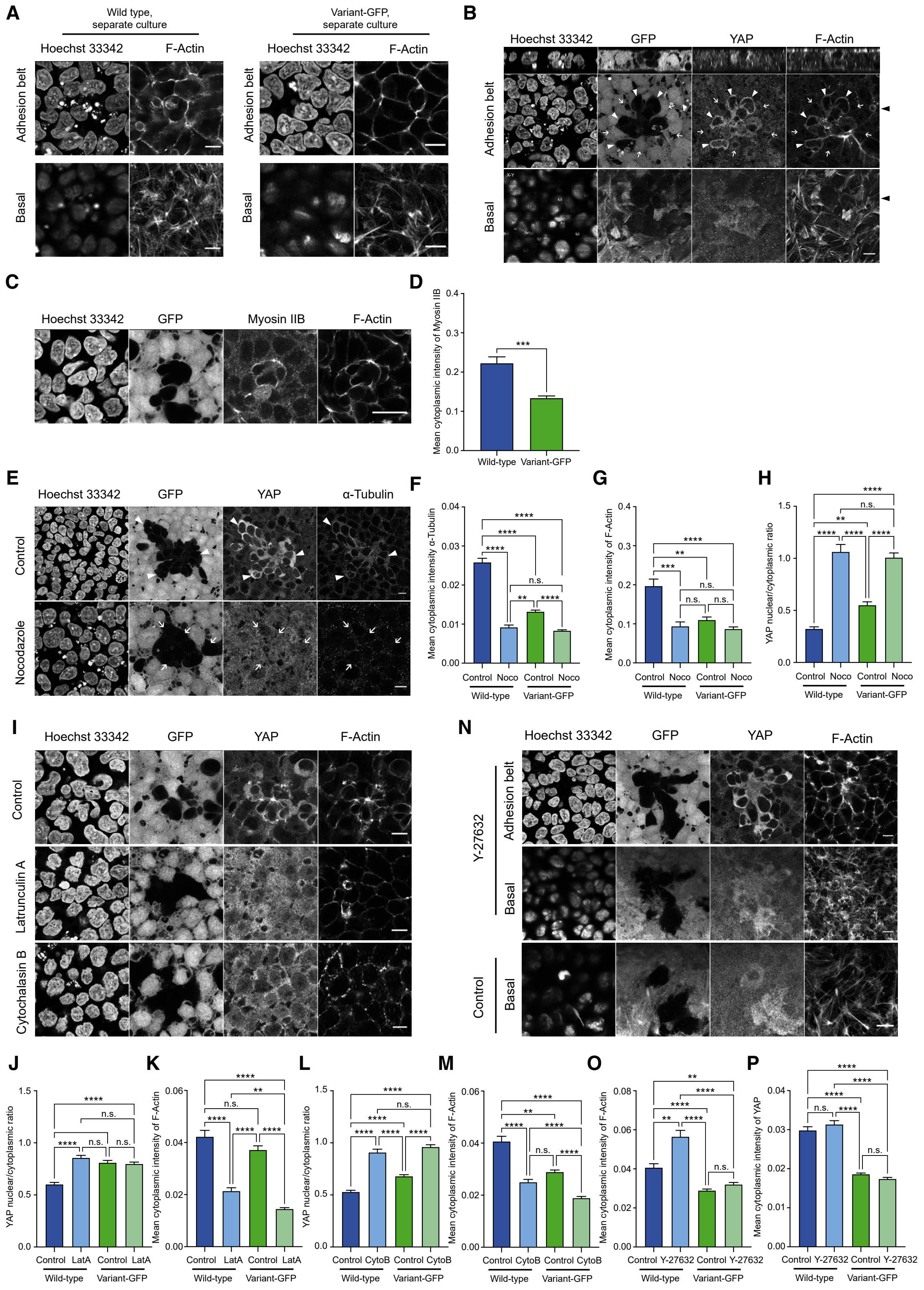

(legend on next page)

of YAP in their cytoplasm and triggers them to commit to apoptosis.

Modulations of cell density and YAP localization in wild-type cells reduce competitive the advantage of variant hPSCs

To identify culture conditions that could reduce the competitive advantage of variant hPSCs, we first tested a pan-caspase inhibitor, z-VAD-FMK, and a ROCK inhibitor, Y-27632, as they have been were previously shown to alleviate mechanical cell competition or cell extrusion in crowded-cell conditions in other models (Saw et al., 2017; Wagstaff et al., 2016). However, neither of the inhibitors rescued hPSC cell competition (Figures S7A–S7E). The pan-caspase inhibitor z-VAD-FMK reduced the elimination of wild-type cells from co-cultures, but the wild-type cells stopped proliferating in crowded cultures (Video S5; Figure S7C). These findings are in line with our data showing that inhibition of apoptosis is not sufficient to confer winner-cell phenotype and with our observation that Y-27632 does not impact on YAP cellular localization of hPSCs at high cell density.

Considering our findings that cell competition in hPSC cultures is cell-density dependent and underpinned by differential localization of YAP in wild-type and variant hPSCs, we reasoned that promotion of YAP nuclear localization in wild-type cells at a high cell density might alleviate the competitive advantage of variant hPSCs. We plated separate and mixed cultures of wild-type and variant cells at increasing cell densities and in the absence or presence of a selective inhibitor of SETD7 methyltransferase inhibitor (*R*)-PFI-2, which has been previously shown to promote YAP nuclear localization (Barsyte-Lovejoy et al., 2014). In wild-type hPSC, (*R*)-PFI-2 also promoted YAP nuclear localization, which is evident from the quantification of the YAP nuclear/cytoplasmic ratio and the intensity of staining of phosphorylated (inactive) YAP in the cytoplasm of wild-type hPSCs in co-culture (Figures 7A and 7B). As stated previously, we observed no cell competition in co-cultures at low cell density, whereas an increase in cell density resulted in a significant shift in ratios of wild-type and variant cells (Figures 7C–7E). Strikingly, the inclusion of (*R*)-PFI-2 suppressed cell competition at high cell densities (Figures 7C–7E). This effect was due to the increased numbers of wild-type cells in co-cultures treated with (*R*)-PFI-2 (Figures 7F–7H). Thus, the effect of (*R*)-PFI-2 is consistent with a key role of YAP in hPSC competition. Moreover, these data demonstrate that cell competition can be suppressed by growing cells at low density or by promoting YAP nuclear localization in conditions of high cell density.

DISCUSSION

Suppressing the commonly arising variant hPSCs from overtaking these cultures requires a thorough understanding of the attributes that facilitate variant cells in achieving the clonal dominance. Here we report that the supremacy of particular variant clones in hPSC cultures is enhanced through competitive interactions with their wild-type counterparts, leading to the elimination of wild-type cells from mosaic cultures. The manner of wild-type cell elimination resembles previously described cell competition (Mamada et al., 2015; Morata and Ripoll, 1975; Sancho et al., 2013) in that the wild-type hPSCs, albeit viable in homotypic cultures, failed to thrive and underwent increased levels of apoptosis when co-cultured with variants. We showed that the winner-cell phenotype was assumed by variant clones, which possessed relatively faster growth rates and achieved higher homeostatic density compared with the loser cells. Thus, cell competition in hPSC cultures is akin to mechanical cell competition (Levayer et al., 2016; Wagstaff et al., 2016).

Our data identified YAP as a key mediator of cell competition in hPSC cultures. First, we detected differences in YAP localization in wild-type cells upon co-culture with their faster-growing variant counterparts. Second, YAP knockdown in variant cells alleviated cell competition in hPSCs. Finally, overexpression of YAP in wild-type cells or promotion of YAP nuclear localization using a chemical inhibitor also partially rescued wild-type cells from elimination by variants. YAP overexpression was previously shown to promote naive cell state of hPSCs (Qin et al., 2016). However, in our study, overexpression of YAP under the primed conditions did not affect the pluripotency state of hPSCs. Thus, in our model system, YAP does not appear to act as a stemness factor, but rather, increased YAP expression or promotion of YAP nuclear retention allows wild-type cells to withstand cell competition in co-cultures with variants. Further work is needed to address specific YAP targets that modulate hPSC fates during cell competition and what contribution an increased resistance to apoptosis of variant cells may play in the winner-cell phenotypes.

It also remains unknown which precise signals upstream of YAP winner hPSCs utilize to communicate their fitness

Figure 6. YAP localization is regulated by adhesion belt actin in hPSCs

(A) F-actin staining at the adhesion belt and basal planes in wild-type (left panels) and variant-GFP (right panels) grown in separate cultures.

(B) F-actin staining at the adhesion belt and basal planes of wild-type and variant-GFP co-cultured cells. Closed arrowheads point to wild-type cells displaying YAP localized within the cytoplasm and having a prominent staining of adhesion belt F-actin. Open arrows point to neighboring variant-GFP cells displaying nuclear localization of YAP and no prominent adhesion belt.

(C) Myosin IIB and F-actin staining within the adhesion belt of co-cultured wild-type and variant-GFP cells.

(D) The mean intensity of myosin IIB in wild-type and variant-GFP cells upon co-culture. ***$p < 0.001$, Kolmogorov-Smirnov test.

(E) Localization of YAP in co-cultured wild-type and variant-GFP cells treated with nocodazole. Closed arrowheads point to wild-type cells displaying YAP localized within the cytoplasm and having prominent staining of α-tubulin. Nocodazole treatment perturbed the microtubule structure and caused diffuse localization of YAP in wild-type cells (open arrows).

(F–H) The mean intensity of α-tubulin (F), F-actin (G), and the nuclear to cytoplasmic ratio of YAP (H), in wild-type and variant-GFP cells ± nocodazole.

(I–M) Disruption of F-actin in the adhesion belt of co-cultured wild-type and variant-GFP cells treated with latrunculin A or cytochalasin B.

(N–P) Treatment of co-cultures with Y-27632.

Data are represented as the mean ± SEM (F–H, J–P); n.s., nonsignificant; **$p < 0.01$, ***$p < 0.001$, ****$p < 0.0001$; one-way ANOVA followed by Kruskal-Wallis multiple comparisons test.

Scale bars: 10 μm.

CellPress
OPEN ACCESS

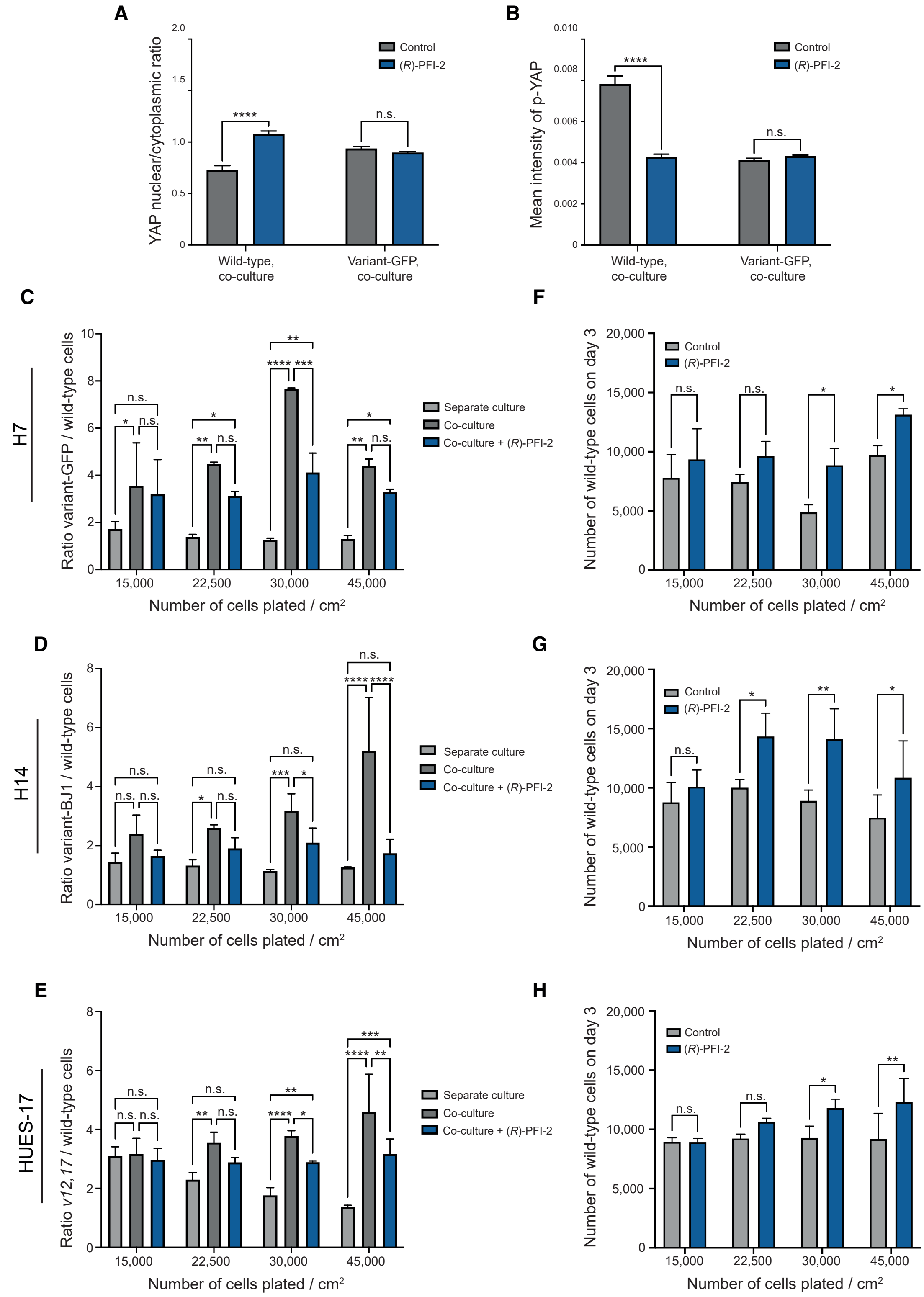

Figure 7. Optimized culture conditions restrict the competitive behavior of winner hPSCs

(A and B) Nuclear to cytoplasmic ratio of YAP (A), and the mean intensity of p-YAP in the cytoplasm (B) of wild-type and variant-GFP hPSCs in co-culture ± 1μM (R)-PFI-2.

(C–E) Ratio of H7 variant-GFP/wild-type (C), H14 variant-BJ1/H14 wild-type (D), and HUES-17 V12,*17*/HUES-17 wild-type cells (E) in separate culture, co-culture and co-culture treated with 1μM (R)-PFI-2 at increasing plating cell density.

(legend continued on next page)

advantage over loser hPSCs. Our study implicated differences in mechanical properties of cells as potential readouts of hPSC cellular fitness. For example, we noted that winner cells exhibit reduced cellular stiffness compared with wild-type loser cells. Reduced stiffness of cancerous cells compared with their noncancerous tissue counterparts was previously shown to result in the altered intercellular force transmission and was linked to the changes in the cytoskeletal organization of cancer cells (Schwager et al., 2019). Gene-expression analysis of winner and loser cells also highlighted differences in genes encoding components of actomyosin cytoskeleton, cell-cell adhesion, and tight-junction proteins. Conceivably, changes in the cytoskeleton and/or adhesion properties of variant cells could be driving a differential response to mechanical cues encountered in the cell-crowding conditions. However, given that commonly amplified regions in the hPSC genome typically span several megabases (Baker et al., 2016) and commonly acquired genetic changes induce a global transcriptional response (Ben-David et al., 2014), it remains difficult to pinpoint potential drivers from mere passenger mutations implicated in variant cell phenotypes (Halliwell et al., 2020).

In conclusion, our work revealed cell competition as an important aspect of cellular interaction of wild-type and variant hPSCs, contributing to a complete overtake of cultures by super-competitive variant clones. Undertaking further detailed analyses of genetic variants that exhibit super-competitive behavior should be informative for impact on regenerative medicine applications. The culture conditions that alleviate cell competition revealed through our work provide a grounding for expansion protocols of hPSCs for research and clinical applications.

Limitations of the study

Here, we have focused on investigating how variants with recurrently acquired aneuploidies overtake hPSC cultures. This question should be extended to other types of genetic changes, including recurrent point mutations that have also been reported in hPSCs (Avior et al., 2021; Merkle et al., 2017). Moreover, it is conceivable that a presence of epigenetic variants could also lead to competitive behavior in hPSC cultures. Future studies are warranted to investigate these questions.

STAR★METHODS

Detailed methods are provided in the online version of this paper and include the following:

- KEY RESOURCES TABLE
- RESOURCE AVAILABILITY
  - Lead contact
  - Materials availability
  - Data and code availability
- EXPERIMENTAL MODEL AND SUBJECT DETAILS
  - Human pluripotent stem cell (hPSC) lines
- METHOD DETAILS
  - Human pluripotent stem cell (hPSC) culture
  - Quantitative PCR (qPCR) for determining copy number changes of target genes
  - Quantitative PCR (qPCR) for determining gene expression
  - Cell competition assay
  - Time-lapse imaging and analysis
  - Conditioned medium experiments
  - Transwell assay
  - Cell confrontation assay
  - Cell compression assay
  - Local density analysis
  - Atomic force microscopy
  - Immunocytochemistry and image quantification
  - Flow cytometry
  - Cell sorting of individual sublines from co-cultures
  - Generation of wild-type-RFP cell lines
  - YAP overexpression
  - YAP knock-down using siRNA
  - Treatment of hPSCs with cytoskeletal inhibitors
  - RNA extraction, sequencing and bioinformatic analysis
  - Western blotting
- QUANTIFICATION AND STATISTICAL ANALYSIS

SUPPLEMENTAL INFORMATION

Supplemental information can be found online at https://doi.org/10.1016/j.devcel.2021.07.019.

ACKNOWLEDGMENTS

We thank Sheffield Genetics Diagnostics Service for cytogenetic analyses. Confocal imaging work was performed at the Wolfson Light Microscopy Facility using the ZEISS LSM880 AiryScan microscope. This work was supported by the Medical Research Council MR/N009371/1 and the UK Regenerative Medicine Platform, MRC reference MR/R015724/1.

AUTHOR CONTRIBUTIONS

I.B., T.A.R., C.J.P., and D.S. conceived and designed the experiments. C.J.P., I.B., D.S., B.A.S., and J.L. performed the experiments. C.J.P., D.S., P.J.G., I.B., S.S., and B.A.S. analyzed the data. I.B., C.J.P., P.J.G., D.S., and T.A.R. wrote the paper.

DECLARATION OF INTERESTS

The authors declare no competing interests.

Received: November 21, 2019
Revised: May 9, 2021
Accepted: July 26, 2021
Published: July 28, 2021

REFERENCES

Andrews, P.W., Banting, G., Damjanov, I., Arnaud, D., and Avner, P. (1984). Three monoclonal antibodies defining distinct differentiation antigens associated with different high molecular weight polypeptides on the surface of human embryonal carcinoma cells. Hybridoma 3, 347–361.

(F–H) Number of wild-type H7 (F), H14 (G), and HUES-17 (H) cells on day 3 of co-culture in control or 1μM (R)-PFI-2 conditions at increasing plating cell density. Fields acquired:16. Data are the mean of two (C and F) or three (D, E, G, and H) independent experiments ± SD. n.s. nonsignificant; *p < 0.05, **p < 0.01, ***p < 0.001, ****p < 0.0001, two-way ANOVA followed by Fisher's LSD test.
See also Figure S7; Video S5.

CellPress
OPEN ACCESS

Andrews, P.W., Ben-David, U., Benvenisty, N., Coffey, P., Eggan, K., Knowles, B.B., Nagy, A., Pera, M., Reubinoff, B., Rugg-Gunn, P.J., et al. (2017). Assessing the safety of human pluripotent stem cells and their derivatives for clinical applications. Stem Cell Rep *9*, 1–4.

Andrews, P.W., Matin, M.M., Bahrami, A.R., Damjanov, I., Gokhale, P., and Draper, J.S. (2005). Embryonic stem (ES) cells and embryonal carcinoma (EC) cells: opposite sides of the same coin. Biochem. Soc. Trans. *33*, 1526–1530.

Aragona, M., Panciera, T., Manfrin, A., Giulitti, S., Michielin, F., Elvassore, N., Dupont, S., and Piccolo, S. (2013). A mechanical checkpoint controls multicellular growth through YAP/TAZ regulation by actin-processing factors. Cell *154*, 1047–1059.

Avery, S., Hirst, A.J., Baker, D., Lim, C.Y., Alagaratnam, S., Skotheim, R.I., Lothe, R.A., Pera, M.F., Colman, A., Robson, P., et al. (2013). BCL-XL mediates the strong selective advantage of a 20q11.21 amplification commonly found in human embryonic stem cell cultures. Stem Cell Rep *1*, 379–386.

Avior, Y., Lezmi, E., Eggan, K., and Benvenisty, N. (2021). Cancer-related mutations identified in primed human pluripotent stem cells. Cell Stem Cell *28*, 10–11.

Baker, D., Hirst, A.J., Gokhale, P.J., Juarez, M.A., Williams, S., Wheeler, M., Bean, K., Allison, T.F., Moore, H.D., Andrews, P.W., et al. (2016). Detecting genetic mosaicism in cultures of human pluripotent stem cells. Stem Cell Rep *7*, 998–1012.

Baker, D.E., Harrison, N.J., Maltby, E., Smith, K., Moore, H.D., Shaw, P.J., Heath, P.R., Holden, H., and Andrews, P.W. (2007). Adaptation to culture of human embryonic stem cells and oncogenesis in vivo. Nat. Biotechnol. *25*, 207–215.

Barbaric, I., Biga, V., Gokhale, P.J., Jones, M., Stavish, D., Glen, A., Coca, D., and Andrews, P.W. (2014). Time-lapse analysis of human embryonic stem cells reveals multiple bottlenecks restricting colony formation and their relief upon culture adaptation. Stem Cell Rep *3*, 142–155.

Barsyte-Lovejoy, D., Li, F., Oudhoff, M.J., Tatlock, J.H., Dong, A., Zeng, H., Wu, H., Freeman, S.A., Schapira, M., Senisterra, G.A., et al. (2014). (R)-PFI-2 is a potent and selective inhibitor of SETD7 methyltransferase activity in cells. Proc. Natl. Acad. Sci. U. S. A. *111*, 12853–12858.

Ben-David, U., Arad, G., Weissbein, U., Mandefro, B., Maimon, A., Golan-Lev, T., Narwani, K., Clark, A.T., Andrews, P.W., Benvenisty, N., et al. (2014). Aneuploidy induces profound changes in gene expression, proliferation and tumorigenicity of human pluripotent stem cells. Nat. Commun. *5*, 4825.

Bondar, T., and Medzhitov, R. (2010). p53-mediated hematopoietic stem and progenitor cell competition. Cell Stem Cell *6*, 309–322.

Bowling, S., Lawlor, K., and Rodríguez, T.A. (2019). Cell competition: the winners and losers of fitness selection. Development *146*, dev167486.

Boyden, S. (1962). The chemotactic effect of mixtures of antibody and antigen on polymorphonuclear leucocytes. J. Exp. Med. *115*, 453–466.

Brás-Pereira, C., and Moreno, E. (2018). Mechanical cell competition. Curr. Opin. Cell Biol. *51*, 15–21.

Brumby, A.M., and Richardson, H.E. (2003). scribble mutants cooperate with oncogenic Ras or Notch to cause neoplastic overgrowth in Drosophila. EMBO J *22*, 5769–5779.

Burke, R., and Basler, K. (1996). Dpp receptors are autonomously required for cell proliferation in the entire developing Drosophila wing. Development *122*, 2261–2269.

Carpenter, A.E., Jones, T.R., Lamprecht, M.R., Clarke, C., Kang, I.H., Friman, O., Guertin, D.A., Chang, J.H., Lindquist, R.A., Moffat, J., et al. (2006). CellProfiler: image analysis software for identifying and quantifying cell phenotypes. Genome Biol *7*, R100.

Catalina, P., Montes, R., Ligero, G., Sanchez, L., de la Cueva, T., Bueno, C., Leone, P.E., and Menendez, P. (2008). Human ESCs predisposition to karyotypic instability: is a matter of culture adaptation or differential vulnerability among hESC lines due to inherent properties? Mol. Cancer *7*, 76.

Chen, G., Gulbranson, D.R., Hou, Z., Bolin, J.M., Ruotti, V., Probasco, M.D., Smuga-Otto, K., Howden, S.E., Diol, N.R., Propson, N.E., et al. (2011). Chemically defined conditions for human iPSC derivation and culture. Nat. Methods *8*, 424–429.

Cowan, C.A., Klimanskaya, I., McMahon, J., Atienza, J., Witmyer, J., Zucker, J.P., Wang, S., Morton, C.C., McMahon, A.P., Powers, D., et al. (2004). Derivation of embryonic stem-cell lines from human blastocysts. N. Engl. J. Med. *350*, 1353–1356.

de la Cova, C., Abril, M., Bellosta, P., Gallant, P., and Johnston, L.A. (2004). Drosophila myc regulates organ size by inducing cell competition. Cell *117*, 107–116.

Draper, J.S., Pigott, C., Thomson, J.A., and Andrews, P.W. (2002). Surface antigens of human embryonic stem cells: changes upon differentiation in culture. J. Anat. *200*, 249–258.

Draper, J.S., Smith, K., Gokhale, P., Moore, H.D., Maltby, E., Johnson, J., Meisner, L., Zwaka, T.P., Thomson, J.A., and Andrews, P.W. (2004). Recurrent gain of chromosomes 17q and 12 in cultured human embryonic stem cells. Nat. Biotechnol. *22*, 53–54.

Eichenlaub, T., Cohen, S.M., and Herranz, H. (2016). Cell competition drives the formation of metastatic tumors in a Drosophila Model of epithelial tumor formation. Curr. Biol. *26*, 419–427.

Enver, T., Soneji, S., Joshi, C., Brown, J., Iborra, F., Orntoft, T., Thykjaer, T., Maltby, E., Smith, K., Abu Dawud, R., et al. (2005). Cellular differentiation hierarchies in normal and culture-adapted human embryonic stem cells. Hum. Mol. Genet. *14*, 3129–3140.

Halliwell, J., Barbaric, I., and Andrews, P.W. (2020). Acquired genetic changes in human pluripotent stem cells: origins and consequences. Nat. Rev. Mol. Cell Biol. *21*, 715–728.

Hashimoto, M., and Sasaki, H. (2019). Epiblast formation by TEAD-YAP-dependent expression of pluripotency factors and competitive elimination of unspecified cells. Dev. Cell *50*, 139–154.e5.

Imreh, M.P., Gertow, K., Cedervall, J., Unger, C., Holmberg, K., Szöke, K., Csöregh, L., Fried, G., Dilber, S., Blennow, E., et al. (2006). In vitro culture conditions favoring selection of chromosomal abnormalities in human ES cells. J. Cell. Biochem. *99*, 508–516.

International Stem Cell Initiative, Amps, K., Andrews, P.W., Anyfantis, G., Armstrong, L., Avery, S., Baharvand, H., Baker, J., Baker, D., Munoz, M.B., et al. (2011). Screening ethnically diverse human embryonic stem cells identifies a chromosome 20 minimal amplicon conferring growth advantage. Nat. Biotechnol. *29*, 1132–1144.

Johnston, L.A. (2014). Socializing with MYC: cell competition in development and as a model for premalignant cancer. Cold Spring Harb. Perspect. Med. *4*, a014274.

Kanehisa, M., Goto, S., Furumichi, M., Tanabe, M., and Hirakawa, M. (2010). KEGG for representation and analysis of molecular networks involving diseases and drugs. Nucleic Acids Res *38*, D355–D360.

Köhler, G., and Milstein, C. (1975). Continuous cultures of fused cells secreting antibody of predefined specificity. Nature *256*, 495–497.

Laing, O., Halliwell, J., and Barbaric, I. (2019). Rapid PCR assay for detecting common genetic variants arising in human pluripotent stem cell cultures. Curr. Protoc. Stem Cell Biol. *49*, e83.

Letunic, I., and Bork, P. (2007). Interactive Tree Of Life (iTOL): an online tool for phylogenetic tree display and annotation. Bioinformatics *23*, 127–128.

Levayer, R., Dupont, C., and Moreno, E. (2016). Tissue crowding induces Caspase-dependent competition for space. Curr. Biol. *26*, 670–677.

Liew, C.G., Draper, J.S., Walsh, J., Moore, H., and Andrews, P.W. (2007). Transient and stable transgene expression in human embryonic stem cells. Stem Cells *25*, 1521–1528.

Liew, C.G., Draper, J.S., Walsh, J., Moore, H., and Andrews, P.W. (2007). Transient and stable transgene expression in human embryonic stem cells. Stem Cells *25*, 1521–1528.

Liu, Z., Yee, P.P., Wei, Y., Liu, Z., Kawasawa, Y.I., and Li, W. (2019). Differential YAP expression in glioma cells induces cell competition and promotes tumorigenesis. J. Cell Sci. *132*, jcs225714.

Love, M.I., Huber, W., and Anders, S. (2014). Moderated estimation of fold change and dispersion for RNA-seq data with DESeq2. Genome Biol *15*, 550.

Mamada, H., Sato, T., Ota, M., and Sasaki, H. (2015). Cell competition in mouse NIH3T3 embryonic fibroblasts is controlled by the activity of Tead family proteins and Myc. J. Cell Sci. *128*, 790–803.

Merkle, F.T., Ghosh, S., Kamitaki, N., Mitchell, J., Avior, Y., Mello, C., Kashin, S., Mekhoubad, S., Ilic, D., Charlton, M., et al. (2017). Human pluripotent stem cells recurrently acquire and expand dominant negative P53 mutations. Nature *545*, 229–233.

Mi, H., Muruganujan, A., Ebert, D., Huang, X., and Thomas, P.D. (2019). PANTHER version 14: more genomes, a new PANTHER GO-slim and improvements in enrichment analysis tools. Nucleic Acids Res *47*, D419–D426.

Moitrier, S., Blanch-Mercader, C., Garcia, S., Sliogeryte, K., Martin, T., Camonis, J., Marcq, P., Silberzan, P., and Bonnet, I. (2019). Collective stresses drive competition between monolayers of normal and Ras-transformed cells. Soft Matter *15*, 537–545.

Morata, G., and Ripoll, P. (1975). Minutes: mutants of Drosophila autonomously affecting cell division rate. Dev. Biol. *42*, 211–221.

Moreno, E., and Basler, K. (2004). dMyc transforms cells into super-competitors. Cell *117*, 117–129.

Moreno, E., Basler, K., and Morata, G. (2002). Cells compete for decapentaplegic survival factor to prevent apoptosis in Drosophila wing development. Nature *416*, 755–759.

Moya, I.M., Castaldo, S.A., Van den Mooter, L., Soheily, S., Sansores-Garcia, L., Jacobs, J., Mannaerts, I., Xie, J., Verboven, E., Hillen, H., et al. (2019). Peritumoral activation of the Hippo pathway effectors YAP and TAZ suppresses liver cancer in mice. Science *366*, 1029–1034.

Neto-Silva, R.M., de Beco, S., and Johnston, L.A. (2010). Evidence for a growth-stabilizing regulatory feedback mechanism between Myc and Yorkie, the Drosophila homolog of Yap. Dev. Cell *19*, 507–520.

Nguyen, H.T., Geens, M., Mertzanidou, A., Jacobs, K., Heirman, C., Breckpot, K., and Spits, C. (2014). Gain of 20q11.21 in human embryonic stem cells improves cell survival by increased expression of Bcl-xL. Mol. Hum. Reprod. *20*, 168–177.

Olariu, V., Harrison, N.J., Coca, D., Gokhale, P.J., Baker, D., Billings, S., Kadirkamanathan, V., and Andrews, P.W. (2010). Modeling the evolution of culture-adapted human embryonic stem cells. Stem Cell Res *4*, 50–56.

Panciera, T., Azzolin, L., Cordenonsi, M., and Piccolo, S. (2017). Mechanobiology of YAP and TAZ in physiology and disease. Nat. Rev. Mol. Cell Biol. *18*, 758–770.

Porazinski, S., de Navascués, J., Yako, Y., Hill, W., Jones, M.R., Maddison, R., Fujita, Y., and Hogan, C. (2016). EphA2 drives the segregation of Ras-transformed epithelial cells from normal neighbors. Curr. Biol. *26*, 3220–3229.

Qin, H., Hejna, M., Liu, Y., Percharde, M., Wossidlo, M., Blouin, L., Durruthy-Durruthy, J., Wong, P., Qi, Z., Yu, J., et al. (2016). YAP induces human naive pluripotency. Cell Rep *14*, 2301–2312.

Sancho, M., Di-Gregorio, A., George, N., Pozzi, S., Sánchez, J.M., Pernaute, B., and Rodríguez, T.A. (2013). Competitive interactions eliminate unfit embryonic stem cells at the onset of differentiation. Dev. Cell *26*, 19–30.

Saw, T.B., Doostmohammadi, A., Nier, V., Kocgozlu, L., Thampi, S., Toyama, Y., Marcq, P., Lim, C.T., Yeomans, J.M., and Ladoux, B. (2017). Topological defects in epithelia govern cell death and extrusion. Nature *544*, 212–216.

Schindelin, J., Arganda-Carreras, I., Frise, E., Kaynig, V., Longair, M., Pietzsch, T., Preibisch, S., Rueden, C., Saalfeld, S., Schmid, B., et al. (2012). Fiji: an open-source platform for biological-image analysis. Nat. Methods *9*, 676–682.

Schwager, S.C., Taufalele, P.V., and Reinhart-King, C.A. (2019). Cell-cell mechanical communication in cancer. Cell. Mol. Bioeng. *12*, 1–14.

Shevinsky, L.H., Knowles, B.B., Damjanov, I., and Solter, D. (1982). Monoclonal antibody to murine embryos defines a stage-specific embryonic antigen expressed on mouse embryos and human teratocarcinoma cells. Cell *30*, 697–705.

Stöver, B.C., and Müller, K.F. (2010). TreeGraph 2: combining and visualizing evidence from different phylogenetic analyses. BMC Bioinformatics *11*, 7.

Suijkerbuijk, S.J., Kolahgar, G., Kucinski, I., and Piddini, E. (2016). Cell competition drives the growth of intestinal adenomas in Drosophila. Curr. Biol. *26*, 428–438.

Supek, F., Bošnjak, M., Škunca, N., and Šmuc, T. (2011). REVIGO summarizes and visualizes long lists of gene ontology terms. PLoS One *6*, e21800.

Thomson, J.A., Itskovitz-Eldor, J., Shapiro, S.S., Waknitz, M.A., Swiergiel, J.J., Marshall, V.S., and Jones, J.M. (1998). Embryonic stem cell lines derived from human blastocysts. Science *282*, 1145–1147.

Wada, K., Itoga, K., Okano, T., Yonemura, S., and Sasaki, H. (2011). Hippo pathway regulation by cell morphology and stress fibers. Development *138*, 3907–3914.

Wagstaff, L., Goschorska, M., Kozyrska, K., Duclos, G., Kucinski, I., Chessel, A., Hampton-O'Neil, L., Bradshaw, C.R., Allen, G.E., Rawlins, E.L., et al. (2016). Mechanical cell competition kills cells via induction of lethal p53 levels. Nat. Commun. *7*, 11373.

Williams, B.P., Daniels, G.L., Pym, B., Sheer, D., Povey, S., Okubo, Y., Andrews, P.W., and Goodfellow, P.N. (1988). Biochemical and genetic analysis of the OKa blood group antigen. Immunogenetics *27*, 322–329.

Ziosi, M., Baena-López, L.A., Grifoni, D., Froldi, F., Pession, A., Garoia, F., Trotta, V., Bellosta, P., Cavicchi, S., and Pession, A. (2010). dMyc functions downstream of Yorkie to promote the supercompetitive behavior of hippo pathway mutant cells. PLoS Genet *6*, e1001140.

STAR★METHODS

KEY RESOURCES TABLE

REAGENT or RESOURCE	SOURCE	IDENTIFIER
**Antibodies**		
Rabbit anti-Cleaved caspase-3	Cell Signalling Technology	9661; RRID:AB_2341188
Rabbit anit-MCL-1	Cell Signalling Technology	5453; RRID:AB_10694494
Rabbit anti-BCL-XL	Cell Signalling Technology	2764; RRID:AB_2228008
Rabbit anti-BCL2	Cell Signalling Technology	2870; RRID:AB_2290370
Mouse anti-β-ACTIN	Proteintech	66009-1-Ig; RRID:AB_2687938
Mouse anti-YAP	Santa Cruz Biotechnology	sc-101199; RRID:AB_1131430
Rabbit anti-Phospho-YAP (Ser127)	Cell Signalling Technology	4911; RRID:AB_2218913
Mouse anti-non-muscle Myosin IIB/MYH10	Abcam	ab684; RRID:AB_305661
Rabbit anti-α-tubulin	Cell Signalling Technology	2144; RRID:AB_2210548
Mouse anti-TRA-1-81 monoclonal	In-house	N/A
Mouse anti-TRA-1-85 monoclonal	In-house	N/A
Rat anti-SSEA3 monoclonal	In-house	N/A
Mouse anti-THY1(CD90)	In-house	N/A
Mouse anti-P3X	In-house	N/A
Goat anti-Mouse AffiniPure IgG+IgM H+L (Alexa Fluor® 647)	Stratech	115-605-044-JIR; RRID:AB_2338906
Goat anti-Rabbit AffiniPure IgG+IgM H+L (Alexa Fluor® 647)	Stratech	111-605-003-JIR; RRID:AB_2338072
Goat anti-Mouse AffiniPure IgG, Fc_{γ} Fragment Specific (Alexa Fluor® 594)	Stratech	115-585-008-JIR;RRID:AB_2338873
Goat anti-Rabbit AffiniPure IgG H+L (Alexa Fluor® 594)	Stratech	111-585-003-JIR;RRID:AB_2338871
Anti-Mouse IgG H+L (HRP)	Promega	W4021; RRID:AB_430834
Anti-Rabbit IgG H+L (HRP)	Promega	W4011; RRID:AB_430833
**Chemicals, peptides, and recombinant proteins**		
Y-27632	Generon	A11001-10
Latrunculin A	Cambridge Bioscience	10010630-25ug-CAY
Cytochalasin B	Sigma-Aldrich	C2743-200UL
Nocodazole	VWR	487928
Z-VAD-FMK	Stratech	A1902-APE-10mg
(*R*)-PFI 2 hydrochloride	Bio-Techne	4892/10
Hoechst 33342, trihydrochide	Fisher Scientific	11534886
Incucyte® Caspase-3/7 Red Dye for Apoptosis	Sartorius	4704
Vitronectin (VTN-N)	Life Technologies	A14700
Sodium selenium	Sigma-Aldrich	S5261
Insulin	Thermo Fisher Scientific	A11382IJ
$NaHCO_3$	Sigma-Aldrich	S5761
Transferrin	Sigma-Aldrich	T0665
Glutamax	Thermo Fisher Scientific	35050038
FGF2	Peprotech	100-18B
Heat Stable Recombinant bFGF	Thermo Fisher Scientific	PHG0367
TGFβ1	Peprotech	100-21
Vectashield Mounting Medium	Vector Laboratories	H-1000
Phalloidin (Alexa Fluor® 647)	Cell Signalling Technology	8940

(*Continued on next page*)

Continued		
REAGENT or RESOURCE	SOURCE	IDENTIFIER
TrypLE Express enzyme	Thermo Fisher Scientific	11528856
ReLeSR	STEMCELL Technologies	05873
DMEM/F12	Sigma-Aldrich	D6421
DMEM/F12 without phenol red	Sigma-Aldrich	D6434
Dulbecco's phosphate buffered saline (PBS)	Sigma-Aldrich	D1408
FastDigest EcoRI	Thermo Fisher Scientific	FD0275
Critical commercial assays		
DNeasy Blood & Tissue Kit	QIAGEN	69504
RNAeasy Plus Mini Kit	QIAGEN	74134
Qubit RNA HS Assay Kit	Thermo Fisher Scientific	Q32855
Silicone culture-inserts 2 well	Ibidi	80209
Transwell 8.0μm PET membrane inserts	Millipore	PIEP12R48
TaqMan Fast Universal Master Mix	Thermo Fisher Scientific	4352042
Deposited data		
RNA-Seq	This paper	ArrayExpress: EMBLE-MTAB-10193
Experimental models: Cell lines		
H7 (WA07) hPSC line	Thomson et al., 1998	RRID:CVCL_9772
H7-H2B-RFP; H7 hPSCs constitutively expressing H2B-RFP (in this study referred to as wild-type-RFP cells)	This paper	RRID:CVCL_A5NJ
H7-YAP; H7 hPSCs overexpressing YAP (in this study referred to as wild-type-YAP)	This paper	RRID:CVCL_A5LL
H7*v1q*; H7 hPSCs with a gain of chromosome 1q (in this study referred to as *v1q*)	This paper	RRID:CVCL_A5KR
H7*v17q*; H7 hPSCs with a gain of chromosome 17q (in this study referred to as *v17q*)	This paper	RRID:CVCL_A5KS
H7*v20q*; H7 hPSCs with a gain of chromosome 20q (in this study referred to as *v20q*)	This paper	RRID:CVCL_A5KT
H7v1,17q,i20; H7 hPSCs with a gain of chromosomes 1, 17q and isochromosome 20 (in this study referred to as *v1,17q,i20*)	This paper	RRID:CVCL_A5LK
H7*v1,12,17q,20q-GFP*; H7.s6-GFP (in this study referred to as variant-GFP)	Baker et al., 2016	RRID:CVCL_A5KW
H14 (WA14) hPSC line	Thomson et al., 1998	RRID:CVCL_9775
H14-H2B-RFP; H14 hPSCs constitutively expressing H2B-RFP (in this study referred to as H14 wild-type-RFP)	This paper	RRID:CVCL_A5NK
H14-YAP; H14 hPSCs overexpressing YAP (in this study referred to as H14 wild-type-YAP)	This paper	RRID:CVCL_A5LM
H14.BJ1-GFP; H14 hPSCs with a gain of chromosomes 12 and 17 and stably expressing GFP (in this study referred to as H14 variant-BJ1)	Baker et al., 2016	RRID:CVCL_A5KX
HUES 17 hPSC line	Cowan et al., 2004	RRID:CVCL_B147
HUES 17-H2B-RFP; HUES 17 hPSCs constitutively expressing H2B-RFP (in this study referred to as HUES-17 wild-type-RFP)	This paper	RRID:CVCL_A5NL

(*Continued on next page*)

CellPress
OPEN ACCESS

Continued		
REAGENT or RESOURCE	SOURCE	IDENTIFIER
HUES 17v12,17; HUES 17 hPSCs with a gain of chromosomes 12 and 17 (in this study referred to as HUES-17*v12,17*)	This paper	RRID:CVCL_A5KC
Oligonucleotides		
PrimeTime qPCR Assay: TERT	Integrated DNA Technologies	Hs.PT.58.27489922
PrimeTime qPCR Assay: KLF5	Integrated DNA Technologies	Hs.PT.56a.40282397
PrimeTime qPCR Assay: KLF4	Integrated DNA Technologies	Hs.PT.58.45542593
PrimeTime qPCR Assay: KLF2	Integrated DNA Technologies	Hs.PT.58.39339409
PrimeTime qPCR Assay: GBX2	Integrated DNA Technologies	Hs.PT.58.803756
PrimeTime qPCR Assay: POU5F1	Integrated DNA Technologies	Hs.PT.58.14648152.g
PrimeTime qPCR Assay: SOX2	Integrated DNA Technologies	Hs.PT.58.237897.g
PrimeTime qPCR Assay: ACTB	Integrated DNA Technologies	Hs.PT.39a.22214847
PrimeTime qPCR Assay: YAP1	Integrated DNA Technologies	Hs.PT.58.14881945
PrimeTime qPCR Assay: TFCP2L1	Integrated DNA Technologies	Hs.PT.58.26495531
PrimeTime qPCR Assay: NANOG	Integrated DNA Technologies	Hs.PT.58.21480849
PrimeTime qPCR Assay: OTX2	Integrated DNA Technologies	Hs.PT.58.46695245
PrimeTime qPCR Assay: DPPA3	Integrated DNA Technologies	Hs.PT.58.2165190
PrimeTime qPCR Assay: TBX3	Integrated DNA Technologies	Hs.PT.58.3646164
PrimeTime qPCR Assay: DNMT3B	Integrated DNA Technologies	Hs.PT.58.5075361
PrimeTime qPCR Assay: GATA6	Integrated DNA Technologies	Hs.PT.58.38396504
PrimeTime qPCR Assay: Lin28A	Integrated DNA Technologies	Hs.PT.58.24268123
PrimeTime qPCR Assay: ZFP42	Integrated DNA Technologies	Hs.PT.58.23001209
TaqMan® Gene Expression Assay: ACTB	ThermoFisher Scientific	Hs99999903_m1
TaqMan® Gene Expression Assay: YAP1	ThermoFisher Scientific	Hs00902712_g1
TaqMan® Gene Expression Assay: CTGF	ThermoFisher Scientific	Hs00170014_m1
TaqMan® Gene Expression Assay: CYR61	ThermoFisher Scientific	Hs00155479_m1
ESIRNA HUMAN YAP1	Sigma-Aldrich	EHU113021-20UG
ESIRNA RLUC	Sigma-Aldrich	EHURLUC-20UG
Recombinant DNA		
pGAMA-YAP	Qin et al., 2016	RRID:Addgene_74942
pCAGeGFP	Liew et al., 2007	N/A
pCAG-YAP	This paper	N/A
pCAG-H2B-RFP	A kind gift from Dr Jie Na, Tsinghua University, Beijing	N/A
Software and algorithms		
Cell Profiler	Carpenter et al., 2006	RRID:SCR_007358
R	R Core Team	RRID:SCR_001905
FlowJo Software v10	FlowJo	RRID:SCR_008520
FIJI	Schindelin et al., 2012	RRID:SCR_002285
DESeq2	Love et al., 2014	RRID:SCR_015687
PANTHER v14	Mi et al., 2019	RRID:SCR_004869
ReViGO	Supek et al., 2011	RRID: SCR_005825
GraphPad Prism 9.0.2	Prism	RRID:SCR_002798
Developer Toolbox 1.7	GE Healthcare	RRID:SCR_015790
QuantStudio 12K Flex Software	Applied Biosystems	N/A
Sortware	BD	N/A
TreeGraph 2	Stöver and Müller, 2010	N/A
Interactive Tree of Life (iTOL)	Letunic and Bork, 2007	N/A
arivis Vision4D	arivis AG	RRID:SCR_018000

(*Continued on next page*)

Continued		
REAGENT or RESOURCE	SOURCE	IDENTIFIER
ZEN Digital Imaging for Light Microscopy	ZEISS	RRID:SCR_013672
Other		
Manual Cell Stretching System	STREX Inc.	STB-100-10
4-well PDMS Stretch Chamber	STREX Inc.	STB-CH-4W

RESOURCE AVAILABILITY

Lead contact

Requests for further information or reagents can be directed to and will be fulfilled by the Lead Contact, Ivana Barbaric (i.barbaric@sheffield.ac.uk), Centre for Stem Cell Biology, Department of Biomedical Science, The University of Sheffield.

Materials availability

Plasmids generated in this study will be made available upon request. All cell lines generated in this study will be made available upon request but we may require a completed materials transfer agreement and reasonable compensation by requestor for its processing and shipping.

Data and code availability

RNA sequencing data related to Figure S5; Tables S1 and S2 has been submitted to ArrayExpress: EMBLE-MTAB-10193.

EXPERIMENTAL MODEL AND SUBJECT DETAILS

Human pluripotent stem cell (hPSC) lines

Wild-type hPSCs used in this study were early passage sublines of H7 (WA07), H14 (WA14) and HUES-17 originally established in the laboratories of James Thomson (Thomson et al., 1998) and Douglas Melton (Cowan et al., 2004), respectively, which were karyotypically normal (based on at least 20 metaphases analysed by G-banding of cell banks prior to experiments and at various time points upon subsequent passaging) and did not possess a commonly gained 20q11.21 copy number variant (as determined by quantitative PCR for copy number changes and/or Fluorescent In Situ Hybridisation (Baker et al., 2016; Laing et al., 2019)). Spontaneous variants with karyotypic abnormalities were detected during the subsequent culture of H7, H14 and HUES-17 cells at the Centre for Stem Cell Biology in Sheffield (Baker et al., 2007; Draper et al., 2004). Genetically variant sublines of H7 line used in this study and their karyotypes were: ‘variant-GFP’ cells [48,XX,+del(1)(p22p22),der(6)t(6;17)(q27;q1),+12,ish dup(20q11.21q11.21)] (30 metaphases analysed), also harbouring chromosome 20q CNV as determined by quantitative PCR analysis and FISH (Baker et al., 2016; Laing et al., 2019); ‘*v1,17q,i20*’ [47,XX, +del(1)(p22p22), der(6)t(6;17)(q27;q1), t(12;20)(q13;q11.2), i(20)(q10) dup(20)(q11.21q11.21)] (30 metaphases analysed); ‘*v17q*’ cells [46,XX,der(6)t(6;17)(q27;q1) (30 metaphases analysed) and ‘*v1q*’ cells [46,XX,dup(1)(q21q42)] (30 metaphases analysed). The variant ‘*v20q*’ appeared to have a diploid karyotype when analysed by G-banding (30 metaphases analysed), but a gain of a copy number variant 20q11.21 was detected by Fluoresecent In Situ Hybridisation and quantitative PCR analysis. The karyotype of the H14 variant subline H14.BJ1-GFP was [48,XY,+12,+der(17)hsr(17)(p11.2) del(17)(p13.3)] (20 metaphases analysed). The karyotype of the HUES-17 variant subline ‘*HUES-17 v12,17*’ was [48,XY,+12,+17] (20 metaphases analysed). Variants *v1q*, *v20q* and *v17q* were established in this study by cloning out spontaneously arising variants from mosaic cultures using single cell deposition by fluorescent activated cell sorting. Single cells from mosaic cultures were sorted directly into individual wells of a 96 well plate using a BD FACS Jazz and cultured to form colonies over 2-3 weeks. The resulting colonies were expanded in culture and subsequently frozen to establish cell banks. At the time of freezing, sister flasks were sent for karyotyping by G-banding and assessment of the relative copy number of commonly identified genetic changes by qPCR as described below.

METHOD DETAILS

Human pluripotent stem cell (hPSC) culture

Flasks used for hPSC maintenance were coated with vitronectin (VTN-N) (Cat. # A14700, Life Technologies) diluted to 5 μg/ml in Dulbecco’s phosphate buffered saline (PBS) and incubated at 37°C for 1h prior to aspirating the vitronectin solution and plating hPSCs. HPSCs were maintained in E8 medium prepared in house, consisting of DMEM/F12 (Cat. # D6421; Sigma-Aldrich) supplemented with 14 μg/l sodium selenium (Cat. # S5261; Sigma-Aldrich), 19.4 mg/l insulin (Cat. # A11382IJ; Thermo Fisher Scientific), 543 mg/l $NaHCO_3$ (Cat. # S5761; Sigma-Aldrich), 10.7 mg/l transferrin (Cat. # T0665; Sigma-Aldrich), 10 ml/l Glutamax (Cat. # 35050038; Thermo Fisher Scientific), 100μg/l FGF2 (Cat. # 100-18B; Peprotech) and 2 μg/l TGFβ1 (Cat. # 100-21; Peprotech) (Chen et al., 2011). For time lapse experiments, E8 was prepared using DMEM/F12 without phenol red (Cat. # D6434; Sigma-Aldrich). Cells were fed daily and maintained at 37°C under a humidified atmosphere of 5% CO_2 in air. Routine passaging every 4-5 days was

performed using ReLeSR (Cat. # 05873; STEMCELL Technologies) according to manufacturer's instructions. Cells were resuspended in E8 and split at 1:3 or 1:4 ratio (wild type cells) or 1:8 to 1:30 ratio (variant sublines). Cells were genotyped after thawing and every 5-8 passages by G-banding, Fluorescent In Situ Hybridization and/or using quantitative PCR for common genetic changes. Karyotyping by G-banding and FISH for chromosome 20q copy number variant were performed by the Sheffield Diagnostic Genetics Service (https://www.sheffieldchildrens.nhs.uk/sdgs/), as previously described (Baker et al., 2016; Laing et al., 2019).

Quantitative PCR (qPCR) for determining copy number changes of target genes

Relative copy number of commonly identified genetic changes was assessed using the qPCR-based approach described in (Baker et al., 2016; Laing et al., 2019). Genomic DNA was extracted from hPSCs using the DNeasy Blood & Tissue Kit (Cat. # 69504; QIAGEN) and digested with FastDigest EcoRI (Cat. # FD0275; Thermo Fisher Scientific) for 2 h at 37°C, followed by inactivation at 65°C for 20 min. PCR reactions were set up in triplicate, with each 10μl PCR reaction containing 1X TaqMan Fast Universal Master Mix (Cat. # 4352042; Thermo Fisher Scientific), 100nM of forward and reverse primers, 100nm of probe from the Universal Probe Library and 10ng of genomic DNA. PCR reactions were run on a QuantStudio 12K Flex Thermocycler (Cat. # 4471087; Life Technologies). Following the first two steps of heating the samples to 50°C for 2 min and denaturing them at 95°C for 10 min, reactions were subjected to 40 cycles of 95°C for 15 s and 60°C for 1 min. The Cq values were obtained from the QuantStudio 12K Flex Software with auto baseline settings and were then exported to Excel for copy number analysis using the relative quantification method (2^{-ddcq}). The calibrator samples for the qPCR assay were hPSC gDNA samples previously established as diploid using karyotyping and Fluorescent In Situ Hybridisation analyses (Baker et al., 2016).

Quantitative PCR (qPCR) for determining gene expression

Expression of YAP target genes and genes associated with the naïve and primed state of pluripotency was assessed using qPCR. RNA was isolated using a Qiagen RNAeasy Plus Mini Kit (Cat. # 74134; Qiagen), and the RNA concentration and purity determined using a NanoPhotometer (Implen, Munich, Germany). cDNA was synthesised using a high-capacity reverse transcription kit (Cat. # 4368814; Thermo Fisher Scientific). qPCR reactions were set up in triplicate, with each 10μl PCR reaction containing 1X TaqMan Fast Universal Master Mix (Cat. # 4352042; Thermo Fisher Scientific), 1X PrimeTime® qPCR Assay (Integrated DNA Technologies) or TaqMan Gene Expression Assay (Thermo Fisher Scientific) and 10ng of cDNA. PCR reactions were run on a QuantStudio 12K Flex Thermocycler (Cat. # 4471087; Life Technologies). Following the first two steps of heating the samples to 50°C for 2 min and denaturing them at 95°C for 10 min, reactions were subjected to 40 cycles of 95°C for 15 s and 60°C for 1 min. The Ct values were obtained from the QuantStudio 12K Flex Software with auto baseline settings and were then exported to the ExpressionSuite Software (Thermo Fisher Scientific) for analysis.

Cell competition assay

Cells were dissociated to single cells using TrypLE (Cat. # 11528856; Thermo Fisher Scientific) for 4 min at 37°C, washed once in DMEM/F12, counted and resuspended in E8 media supplemented with 10μM Y-27632 (Cat. # A11001-10; Generon). Cells were plated as separate cultures of each subline or mixed cultures of different sublines, as described in the individual experiments. After 24h, the medium was removed and the wells were washed once with basal medium DMEM/F12 (Cat. # D6421; Sigma-Aldrich) to remove the Y-27632. The medium was replaced with E8 and that point was considered as 'day 0' of competition experiments. Cells were cultured for further 72 hours and fed daily with E8 medium. Cells were fixed at different time points post-plating in 4% paraformaldehyde (PFA) for 15 min at room temperature, and nuclei stained with 10μg/ml Hoechst 33342 (Thermo Fisher Scientific). In every mixing experiment, one of the sublines used was fluorescently labelled (e.g. either variant-GFP mixed with other non-labelled sublines or wild type-RFP mixed with other wild type or variant sublines), thus allowing identification of cell numbers of each of the sublines in mixed cultures. Imaging of either the entire well or 16 random fields within the well was performed using the InCell Analyzer (GE Healthcare) high-content microscopy platform. Quantification of total and individual subline cell numbers was performed either using custom protocols in Developer Toolbox 1.7 software (GE Healthcare) or CellProfiler (Carpenter et al., 2006). 'Separate culture' ratio was calculated by dividing the number of variant cells in separate culture with a number of wild-type cells in separate culture. 'Co-culture' ratio was obtained by directly counting the number of either wild-type or variant cells in co-culture using high-content microscopy and dividing by the total cell count.

For growth curve analysis, cells were plated at $4{,}4\text{x}10^4$ cells/cm^2 in separate cultures or co-cultures, with the co-cultures containing 50:50 ratio of different sublines (i.e. $2{,}2\ \text{x}10^4$ cells/cm^2 of each subline). As an additional control, separate cultures were also plated containing equivalent numbers of cells from co-cultures (i.e. $2{,}2\ \text{x}10^4$ cells/cm^2 for each subline). For cell competition assays of *v1,17q,i20* cells with variant-GFP cells, cells were plated at the lower density of 22,500 cells/cm^2 due to the faster growth rate of both sublines with a complex karyotype. Cells were fixed with 4% PFA at different time points post-plating and the cell numbers analysed as described above.

For assessing the effect of increasing ratios of variant cells on wild-type cell growth, wild type and variant-GFP cells were plated in E8 supplemented with 10μM Y-27632 (Cat. # A11001-10; Generon) at the total number of $4{,}4\text{x}10^4$ cells/cm^2, with the ratio of variant cells varying from 10% to 90% of the total cell number. After the initial 24h post-plating, cells were washed with DMEM/F12 (Cat. # D6421; Sigma-Aldrich) to remove the Y-27632 and then grown in E8 for further 3 days. Cells were then fixed with 4% PFA and the cell numbers analysed as described above.

For assessing the effect of increasing cell density on wild-type cell growth, wild type and variant-GFP cells were plated at a 50:50 ratio, at cell densities increasing from 3,750 to 45,000 cells/cm^2. After the initial 24h post-plating, cells were washed with DMEM/F12

(Cat. # D6421; Sigma-Aldrich) to remove the Y-27632 and then grown in E8 for further 3 days. Four days post-plating, cells were fixed with 4% PFA and the cell numbers analysed as described above.

Time-lapse imaging and analysis

Time-lapse microscopy was performed at 37°C and 5% CO_2 using either a Nikon Biostation CT or a ZEISS LSM 880 (Carl Zeiss AG, Oberkochen, Germany) fitted with an Airyscan detection unit. For lineage analysis, cells were imaged on a Nikon Biostation CT every 10 min for 72 h using 10x or 20x air objectives. Image stacks were compiled in CL Quant (Nikon) and exported to FIJI (Image J) (Schindelin et al., 2012) for analysis. Lineage trees were constructed manually from FIJI movies. Individual cells were identified in the first frame and then tracked in each subsequent frame until their death, division or the end of the movie. The timing of cell death or division for each cell was noted and then used to reconstruct lineage trees of founder cells using either TreeGraph 2 (Stöver and Müller, 2010) or Interactive Tree Of Life (iTOL) (Letunic and Bork, 2007) software.

For the cell confrontation assay, cells were imaged on either a Nikon Biostation CT every 4 hours for 48 hours using 10x or 20x air objectives, or a ZEISS LSM 880 (Carl Zeiss AG, Oberkochen, Germany) fitted with an Airyscan detection unit every 1 hour for 48 hours. For images acquired using the Biostation CT, image stacks were compiled and analysed to determine the cell area covered by live caspase-3/7 signal in CL Quant (Nikon). For images acquired on the ZEISS LSM 880, a z-stack of 10μm starting below the central position of the nucleus and finishing beyond the apical surface was captured at each timepoint. The images were processed in ZEN (ZEISS) software and exported to FIJI (Image J) (Schindelin et al., 2012) for analysis.

Imaging of the cell competition assay with live caspase-3/7 dye was performed on the ZEISS LSM 880 (Carl Zeiss AG, Oberkochen, Germany) fitted with an Airyscan detection unit every 10 min for 15 hours. A z-stack of 10μm starting below the central position of the nucleus and finishing beyond the apical surface was captured at each timepoint and processed in ZEN (ZEISS) software. The processed image data was rendered into a 4D movie using arivis Vision4D (arivis AG) software.

Conditioned medium experiments

Wild-type and variant sublines of H7, H14 and HUES-17 were plated at $4x10^4$ cells/cm^2 and pre-cultured independently for 24h in the medium supplemented with 10μM Y-27632 (Cat. # A11001-10; Generon). To negate problems with FGF degradation and pH acidosis of conditioned medium, the recipe for E8 was altered to contain 40ng/ml of Heat stable FGF (Cat# PHG0367; ThermoFisher) and an additional 10mM of sodium bicarbonate. Cells were changed with fresh medium and cultured for another 24 hours to condition the medium. Medium was aspirated from the flasks and passed through a 0.22μm filter to remove any cells that had been lifted. The cells in the flasks were washed with DMEM/F12 (Cat. # D6421; Sigma-Aldrich) and medium was changed to either fresh medium, medium conditioned on corresponding wild-type sublines or medium conditioned on variant sublines. Cells were cultured for a further 24 hours before harvesting and staining for flow cytometric analysis of cleaved caspase-3 as described below.

Transwell assay

For indirect co-culture, Millipore Transwell 8.0μm PET membrane inserts (Cat. # PIEP12R48; Millipore) were used in combination with 24 well plates. Both the insert and well were coated with vitronectin (VTN-N) (Cat. # A14700, Life Technologies) diluted to 5 μg/ml in PBS. Cells were harvested using TrypLE (Cat. # 11528856; Thermo Fisher Scientific) and $1.5x10^4$ cells were seeded in the well and insert. Cells were pre-cultured independently for 24h in E8 medium supplemented with 10μM Y-27632 (Cat. # A11001-10; Generon) to facilitate cell attachment. 24h post-plating, cells were washed with DMEM/F12 (Cat. # D6421; Sigma-Aldrich) to remove the Y-27632 and inserts were subsequently placed into appropriate wells with fresh E8 medium. Medium was changed daily until the end of the experiment when the cells were fixed with 4% PFA.

Cell confrontation assay

Cells were harvested using TrypLE (Cat. # 11528856; Thermo Fisher Scientific) and washed once in DMEM/F12 (Cat. # D6421; Sigma-Aldrich). After counting, $5x10^4$ cells were seeded in E8 medium supplemented with 10μM Y-27632 (Cat. # A11001-10; Generon) into the inner compartment of two-well silicone inserts (Ibidi 80209). One day post-plating the silicone inserts were removed, leaving a defined 500μm gap between the two cell populations. The cells were then washed with DMEM/F12 (Cat. # D6421; Sigma-Aldrich) to remove Y-27632 and the medium was replaced with a fresh E8 medium. Cells were fed daily and left to grow for four days until the two opposing cell fronts had been in contact for approximately 48h. Cells were then fixed with 4% PFA for 15 min at room temperature, followed by washing in PBS. Cells were subsequently stained for the apoptotic marker cleaved caspase-3 (Cat. # 9661; Cell Signaling Technology) and nuclei were counterstained with Hoechst 33342 (Cat. # H3570; Thermo Fisher Scientific). Images were processed in CellProfiler (Carpenter et al., 2006) to identify wild-type, wild-type-RFP and variant-GFP cells. Using the nuclei stain, each cell was assigned a positional identity relative to the border and further analyzed for positive cleaved caspase-3 signal. Using the positional information of each cell, figures displaying the location of each cell, as well as cleaved caspase-3 positive cells were constructed in R (R Project for Statistical Computing; RRID:SCR_001905).

Cell compression assay

PDMS chambers with 4 wells (Cat. # STB-CH-4W, STREX Inc.) were stretched by 35% in a uniaxial direction over the resting length on a stretching device (Cat. # STB- 100-10, STREX Inc.). Each well was coated with vitronectin (VTN-N) (Cat. # A14700, Life Technologies) diluted to 5 μg/ml in PBS. Cells were harvested using TrypLE (Cat. # 11528856; Thermo Fisher Scientific) and washed once

in DMEM/F12 (Cat. # D6421; Sigma-Aldrich). After counting, cells were seeded at 500,000 cells/cm^2 and 400,000 cells/cm^2 per well in E8 medium supplemented with 10µM Y-27632 (Cat. # A11001-10; Generon), forming a confluent monolayer of wild-type and variant-GFP hPSCs respectively, and allowing for both conditions to be assessed on the same stretched chamber. The cells were left to attach for 16h and then medium was removed and the wells were washed twice with basal medium DMEM/F12 (Cat. # D6421; Sigma-Aldrich) to remove the Y-27632. The medium was replaced with E8 and incubated for a further 4h, following which the stretch was released to induce compaction. 5h after release the cells were then fixed with 4% PFA for 15 min at room temperature. In parallel, cells were seeded onto unstretched membranes and treated as uncompressed controls. Cells were subsequently stained for the apoptotic marker cleaved caspase-3 (Cat. # 9661; Cell Signaling Technology) and nuclei were counterstained with Hoechst 33342 (Cat. # H3570; Thermo Fisher Scientific). The membranes from individual wells were dissected from the chamber and mounted onto slides in 10 µl Vectashield Mounting Medium (Cat. # H-1700; Vector Laboratories). Imaging of 64 random fields from each well was performed using the InCell Analyzer (GE Healthcare) high-content microscopy platform. Images were processed in CellProfiler (Carpenter et al., 2006) to identify nuclei and further analysed for positive cleaved caspase-3 signal.

Local density analysis

To compute the local density of each wild-type and variant-GFP cell from either separate or co-culture conditions, images of the entire corresponding wells at day 3 were analyzed. Nuclei within each field were identified using custom protocols in CellProfiler (Carpenter et al., 2006) and assigned corresponding XY coordinates. The resulting data was processed in the programming language R. Delaunay triangulation was performed on each image by using the cell nuclei position as points for the triangulation. For each cell, the sum of areas of Delaunay triangles sharing a vertex with the cell of interest was calculated. As this sum is inversely proportional to the compactness of the cells, local density is taken as the inverse of this sum. Mathematically, the local density ρ for each cell is defined as:

$$\rho = \sum 1/A(i) \text{ for } i = 1, \ldots, n$$

where n is the number of Delaunay triangles that share a vertex with the cell of interest, and A(i) is the area of Delaunay triangle i.

Atomic force microscopy

Cells were harvested using TrypLE (Cat. # 11528856; Thermo Fisher Scientific) and washed once in DMEM/F12 (Cat. # D6421; Sigma-Aldrich). After counting, cells were seeded onto 9.5cm^2 dishes (Cat. # 93040; TPP) in E8 medium supplemented with 10µM Y-27632 (Cat. # A11001-10; Generon) at either 1.5x10^4 cells/cm^2 for wild-type (H7 or H14), or 5.0x10^3 cells/cm^2 for variant-GFP and variant-BJ1 cells. After 24h, the medium was removed, and the cells were washed once with basal medium DMEM/F12 (Cat. # D6421; Sigma-Aldrich) to remove the Y-27632. The medium was replaced with E8 and cells cultured for a further 24hrs. Atomic Force measurements were carried out using a Nanowizard III system (JPK Instruments) with a heated sample stage (37°C). The tip-less probe (Cat. # MLCT-O10; Bruker) with a nominal spring constant of 0.02N/m, was prepared with the addition of a 5µm polystyrene sphere. Prior to measurements, the spring constant was determined using the thermal vibration method and the cantilever deflection sensitivity was calibrated from the hard contact regime of force-distance curves measured in E8 medium at 37°C. Small colonies with between 3-8 cells were identified and measurements from at least two cells per colony were taken from approximately the centre of the cell. Between 5-10 measurements were taken for each cell. For each measurement an approach speed of 2µm/s was used to reduce viscus drag. Following each experiment, final measurements were taken on a section of the cell culture dish devoid of cells to ensure the cantilever was not contaminated. The resultant force-indentation curves were analysed using the JPK SPM Data Processing software. The Young's modulus of each cell was determined using the classical Hertz model as this is appropriate for small indentations, a Poisson's ratio of 0.5 was assumed.

Immunocytochemistry and image quantification

Cells were fixed with 4% PFA for 15 min at room temperature, and permeabilised with either 0.5% Triton-X in Dulbecco's phosphate buffered saline (PBS) for 10 min or 0.2% Triton-X in PBS for 1h. Cells were then incubated with 1% bovine serum albumin (BSA) and 0.3% Triton X-100 in PBS. Primary and secondary antibodies, their suppliers and the dilutions used are listed in the Key Resources Table. Cells were incubated with primary antibodies either for 1h at room temperature or overnight at 4°C with gentle agitation on an orbital shaker. Following three washes with PBS, cells were incubated with an appropriate secondary antibody in PBS supplemented with 1% BSA, 0.3% Triton X-100 and 10µg/ml Hoechst 33342 for 1h at 4°C. Cells were then washed three times with PBS before imaging. Cells that were prepared for confocal imaging were grown on glass coverslips and mounted onto slides in 10 µl Vectashield Mounting Medium (Cat. # H-1000; Vector Laboratories). Images were captured using the InCell Analyzer (GE Healthcare) or ZEISS LSM 880 (Carl Zeiss AG, Oberkochen, Germany) fitted with an Airyscan detection unit.

For quantification of YAP, p-YAP, actin, tubulin and myosin IIB images taken on the Airyscan at 40x magnification as an average intensity projection of 10 slices. Briefly, Hoechst33342 staining was used first to segment the nuclei from images; difficult to separate nuclei were manually segmented using Adobe Photoshop to draw a nuclear border between cells. CellProfiler (Carpenter et al., 2006) was then used to analyse the images. After nuclei identification from the edited Hoechst images, the nuclei were dilated and eroded by 15 pixels to give cytoplasm and inner nuclei masks, respectively. The mean intensity for YAP staining was quantified for the cyto-

plasm and inner nuclei and the nuclear to cytoplasmic ratio was calculated using these values. For phospho-YAP, actin, tubulin and myosin IIB the mean intensity of the cytoplasmic signal (using the same mask described above) of each cell was quantified.

Flow cytometry

Flow cytometry for cleaved caspase-3 was performed to assess levels of apoptotic cells in cultures. To collect apoptotic cells which had detached from the flask, the old media was added to a 5ml FACS tube and centrifuged at 270 x *g* for 5 min. Remaining cells in the flask were harvested with TrypLE (Cat. # 11528856; Thermo Fisher Scientific) and added to the FACS tube containing the collected cells from the supernatants of the same flasks. The collated sample was pelleted and the cell pellet fixed in 4% PFA for 15 min at room temperature. Cells were permeabilised with 0.5% Triton X-100 in PBS for 5 min at room temperature and then incubated with anti-cleaved caspase-3 primary antibody (Cat. # 9661; Cell Signalling Technology) in the blocking buffer (1% BSA and 0.3% Triton X-100 in PBS). Samples were gently agitated for 1h at room temperature, prior to washing three times in blocking buffer and staining with secondary antibody (Goat anti-Rabbit AffiniPure IgG+IgM (H+L), Cat. # 111-605-003-JIR; Stratech) for 1h at room temperature in the dark. Cells were then washed twice with blocking buffer and analysed on BD FACS Jazz. Baseline fluorescence was set using secondary antibody-only stained samples.

For intracellular analysis of YAP, cells were harvested with TrypLE, permeabilised and blocked as described above. Cells were incubated with anti-YAP antibody (Cat. # sc-101199; Santa Cruz Biotechnology) and gently agitated for 1h at room temperature, prior to washing three times in blocking buffer and staining with secondary antibody (Goat anti-Mouse AffiniPure IgG+IgM (H+L), Cat. # 115-605-044-JIR; Stratech) for 1h at room temperature in the dark. Cells were then washed twice with a blocking buffer and analysed on BD FACS Jazz. Baseline fluorescence was set using secondary antibody-only stained samples.

For analysis of pluripotency-associated surface antigens, cells were harvested with TrypLE as described above and resuspended in PBS supplemented with 10% Foetal Calf Serum (FCS) at $1x10^7$ cells/mL. Cells were incubated with primary antibodies at the appropriate dilution for 30 mins at 4°C, prior to washing with PBS supplemented with 10% FCS and incubating with secondary antibody (Goat anti-Mouse AffiniPure IgG+IgM (H+L), Cat. # 115-605-044-JIR; Stratech) at 1:200 for 30mins at 4°C in the dark. Cells were then washed twice with PBS supplemented with 10% FCS and analysed on BD FACS Jazz. Baseline fluorescence was set using the primary antibody control P3X, an IgG1 antibody secreted from the parental myeloma cell line P3X6Ag8, which does not bind to any epitopes on human cells (Köhler and Milstein, 1975). The primary monoclonal antibodies TRA-1-85 (Williams et al., 1988), SSEA3 (Shevinsky et al., 1982), TRA-1-81 (Andrews et al., 1984) and THY1(CD90) (International Stem Cell et al., 2011) were prepared in-house as described previously (Draper et al., 2002; International Stem Cell et al., 2011).

Cell sorting of individual sublines from co-cultures

After establishing that variant *v1q* cells are losers in co-cultures with variant-GFP cells, we performed mixing experiments of *v1q* and variant-GFP cells in T75 flasks, following the same protocol as in 96 well plates. Briefly, cells were plated at $4{,}4x10^4$ cells/cm^2 in E8 supplemented with Y-27632 (Cat. # A11001-10; Generon). After 24 h, Y-27632 was removed and cells were cultured in separate or mixed cultures for another day. Cells were then harvested using TrypLE (Cat. # 11528856; Thermo Fisher Scientific) for 4 min at 37°C, washed with DMEM/F12 (Cat. # D6421; Sigma-Aldrich), counted and resuspended at $2x10^6$ cells/ml in E8 media. Sorting was performed using BD FACSJazz cell sorter (BD Biosciences). Sort gates were set using the separate culture unlabelled *v1q* cells and variant-GFP separate cultures as baseline and positive gates, respectively. GFP-negative *v1q* and GFP-positive variant-GFP cells were sorted into collection vessels at $5x10^5$ cells per sample. Samples were re-analysed post sorting to establish the purities. In all cases a minimum purity of 98% was achieved. Separate cultures were also put through the same sorting procedure as co-cultures. Samples were centrifuged at 270 x *g* for 3 min, supernatant removed and cell pellets stored at -80°C prior to RNA or protein extraction. Samples from four independent experiments were obtained for further analyses.

Generation of wild-type-RFP cell lines

To generate the wild-type-RFP line, karyotypically diploid H7, H14 and HUES-17 sublines were transfected with pCAG-H2B-RFP plasmid (a kind gift from Dr Jie Na, Tsinghua University, Beijing) using either the 4D nucleofector (Lonza) in the "P3 Primary Cell solution" as per the manufacturer's instructions or Neon Transfection System (Cat. # MPK10025; Thermo Fisher Scientific). H7 wild-type in the 4D nucleofector were pulsed using the CB-150 pulse code, optimised for hPSCs. H14 and HUES-17 wild-type sublines were dissociated to single cells using TrypLE as described above and resuspended at $2{,}0\ x10^4$ cells/ml in "R buffer". Transfection was performed with 5μg of plasmid DNA using 1 pulse of 1600V, 20msec width. Following transfection, H7, H14 and HUES-17 cells were then plated into flasks coated with Geltrex (Cat. # A1413202; ThermoFisher Scientific) in mTESR1 medium (Cat. # 85850; STEMCELL Technologies) supplemented with 10μM Y-27632 (Cat. # A11001-10; Generon). After two days, the stably transfected cells were selected by growing in medium supplemented with puromycin (Cat. # A11138; Thermo Fisher Scientific). Resistant colonies were manually picked and expanded. Clonal lines were then screened for their RFP expression levels by fluorescent imaging. The chosen clone was karyotyped by G-banding and screened for common genetic changes by quantitative PCR prior to freezing and at regular intervals (~5 passages) upon subsequent culture.

YAP overexpression

The pCAG-YAP expression vector was established by inserting a YAP-T2A-mCherry sequence into the pCAGeGFP vector (Liew et al., 2007). In brief, pGAMA-YAP, a gift from Miguel Ramalho-Santos (Cat. # 74942; Addgene) (Qin et al., 2016), was obtained

from Addgene. Single digests were performed on the pGAMA-YAP and pCAGeGFP vectors using EcoRI (Cat. # 0101, New England Biolabs) and NotI (Cat. # 0189, New England Biolabs) restriction sites, respectively, to linearize plasmids. The cohesive ends were blunted using T4 DNA polymerase (Cat. # M0203, New England Biolabs) and vectors subsequently digested at the NheI restriction site to produce single cohesive ends. The YAP-T2A-mCherry sequence was obtained by gel extraction (Cat. # 740609, Machery-Nagel) and inserted into the pCAGeGFP using ligation reaction (Cat. # M0202, New England Biolabs) to produce the pCAG-YAP expression vector. To generate the wild-type YAP overexpressing line, cells were transfected using the Neon Transfection System (Cat. # MPK10025; Thermo Fisher Scientific). Wild-type H7 or H14 cells were dissociated to single cells using TrypLE as described above and resuspended at 2,0 x10^4 cells/ml in “R buffer”. Transfection was performed with 5μg of plasmid DNA using 1 pulse of 1600V, 20msec width. After electroporation, the cells were immediately transferred to a vitronectin coated 60mm diameter culture dish (Cat. # 150288; Thermo Fisher Scientific) containing E8 media supplemented with 10μM Y-27632 (Cat. # A11001-10; Generon). To select for stably transfected cells, 48h post transfection cells were subjected to puromycin (Cat. # A11138; Thermo Fisher Scientific) drug selection. Individual colonies of resistant cells appeared after 1-2 weeks and were handpicked by micropipette, and transferred into a 12-well culture plate. The cells were then expanded in the presence of puromycin selection and subsequently frozen to establish cell banks. At the time of freezing, cells from sister flasks were karyotyped by G-banding and assessed for the relative copy numbers of commonly identified genetic changes by qPCR, as described above. Upon defrosting and subsequent culture, cells were also regularly genotyped by karyotyping and screened for common genetic changes by quantitative PCR, as described above.

YAP knock-down using siRNA

To knockdown the expression level of YAP in variant cells, we used MISSION esiRNA for YAP (ESIRNA HUMAN YAP1, Cat. # EHU113021-20UG; Sigma-Aldrich) and MISSION esiRNA for Renilla Luciferase as a control (ESIRNA RLUC Cat. # EHURLUC-20UG; Sigma-Aldrich). A 500μl transfection reaction included 50 nM siRNA and 5.6 μl DharmaFECT 1 Transfection Reagent (Cat. # T-2001-03; Horizon Discovery Ltd) in Opti-MEM I Reduced Serum Medium (Cat. # 10149832; Thermo Fisher Scientific). The reactions were incubated for 30 min at room temperature before mixing with 375,000 variant cells in mTESR supplemented with 10μM Y-27632. Variant cells were plated in a 96-well plate at 15,000 cells per well or in a 12-well plate at 150,000 cells per well. After 18 hours, the siRNA was removed from cells in both the 96-well and 12-well plates. In 96-well plates, variant cells in control wells were fed with fresh mTESR supplemented with 10μM Y-27632 (separate cultures of variant cells), whereas in the remaining wells, wild-type cells were plated at 10,000 cells/well in mTESR supplemented with 10μM Y-27632 (co-cultures of variant and wild-type cells). Cells in the 12-well plates were fed with fresh mTESR only. At 24 hours after plating the wild-type cells in 96-well plates, the medium was replaced with fresh mTESR (without Y-27632), denoting ‘day 0’ of the experiment. Medium was changed daily in both the 96-well and 12-well plates. Cells in 12-well plates were dissociated on Day 2 of the experiment and analysed for YAP expression by immunostaining followed by flow cytometry. Cells in 96-well plates were fixed with 4% PFA on Day 3 of the experiment, stained with Hoechst 33342 and imaged using the InCell Analyser 2000.

Treatment of hPSCs with cytoskeletal inhibitors

HPSCs were treated with either 10 μM nocodazole (Cat. #487928; VWR International), or 10 μM Y-27632 (Cat. # A11001-10; Generon) for 3h or 0.5 μM latrunculin A (Cat. # 10010630-25ug-CAY; Cambridge Bioscience) or 0.5 μM cytochalasin B (Cat. # C2743-200UL; Sigma-Aldrich) for 1h. DMSO was used as vehicle control for nocodazole, cytochalasin B and Y-27632, whereas ethanol was used as vehicle control for latrunculin A. Cells were fixed with 4% PFA for 15 min at room temperature, washed in PBS and processed for immunocytochemistry as detailed above.

RNA extraction, sequencing and bioinformatic analysis

Four biological replicates of *v1q* and variant-GFP cells FACS-sorted from either separate or mixed cultures were used for RNA extraction and RNAseq analysis. The RNA was isolated using a Qiagen RNAeasy Plus Mini Kit (Cat. # 74134; Qiagen), and the RNA concentration and purity determined using a Qubit 3.0 Fluorometer (Life Technologies, Carlsbad, USA) and NanoPhotometer (Implen, Munich, Germany), respectively. The libraries were constructed and sequenced by Novogene (Beijing, China). Briefly, libraries were prepared using NEBNext Ultra RNA Library Prep Kit for Illumina (New England Biolabs, Ipswich, USA) and the library preparations were sequenced on an Illumina Hiseq platform (Illumina, San Diego, USA) to generate 150 bp paired-end reads. The sequencing reads were aligned to a reference human genome using TopHat v2.0.12. Raw read counts were calculated using the HTSeq v0.6.1 and were normalized into the fragments per kilobase of transcript per million mapped reads (FPKM), based on the length of the gene and reads count mapped to it. Differential gene expression analysis was performed using the DESeq R package (1.18.0) (Love et al., 2014). Genes with the Benjamini and Hochberg’s adjusted p value of < 0.05 were considered differentially expressed. To identify potential signaling pathways within differentially expressed genes, KEGG enrichment analysis of differentially expressed genes was performed using the PANTHER v14 software (Mi et al., 2019). The resulting list was refined using REViGO (Supek et al., 2011) to remove redundant GO terms.

Western blotting

Cells were lysed in 1x Laemmli Buffer pre-warmed to 95°C and the total protein concentration was normalised using the Pierce BCA Protein Assay (Cat. # 23250; ThermoFisher Scientific). Proteins (10μg/sample) were resolved by SDS-PAGE and were run alongside a Page Ruler prestained protein ladder (Cat. # 26616; ThermoFisher Scientific). Proteins were then transferred onto a PVDF membrane

(Cat. # IPVH00010; Millipore) using an Electrophoresis Transfer Cell (Bio-Rad). The membrane was blocked in 5% milk for one hour, washed three times with TBS-T (50 mM Tris-HCl (pH 7.5), 150 mM NaCl, 0.1% (v/v) Tween 20) and then incubated with primary antibodies for MCL-1 (Cat. # 5453; Cell Signalling Technology) at 1:1,000 dilution, BCL-XL (Cat. # 2764; Cell Signalling Technology) at 1:1,000 dilution, BCL2 (Cat. # 2870; Cell Signalling Technology) at 1:1,000 or β-ACTIN (Cat. #66009-1-Ig; Proteintech) at 1:5,000 dilution. Following three washes with TBS-T, the membrane was incubated with secondary antibody (either Anti-Rabbit IgG (H+L), HRP conjugate (Cat. # W4011; Promega) at 1:4,000 dilution or Anti-Mouse IgG (H+L), HRP conjugate Cat. # W4021; Promega) at 1: 4,000 dilution for 1h. After three washes, immunoreactivity was visualised using ECL Prime detection kit (Cat. # RPN2232, GE Healthcare) and signal captured on a CCD-based camera (Syngene).

QUANTIFICATION AND STATISTICAL ANALYSIS

Statistical analysis of the data presented was performed using GraphPad Prism version 9.0.2, GraphPad Software, La Jolla California USA, www.graphpad.com. Differences were tested by statistical tests including Student's *t* test, one-way ANOVA or two-way ANOVA as indicated in figure legends.

Developmental Cell, Volume *56*

Supplemental information

Genetically variant human pluripotent stem cells selectively eliminate wild-type counterparts through YAP-mediated cell competition

Christopher J. Price, Dylan Stavish, Paul J. Gokhale, Ben A. Stevenson, Samantha Sargeant, Joanne Lacey, Tristan A. Rodriguez, and Ivana Barbaric

Supplementary Figure 1

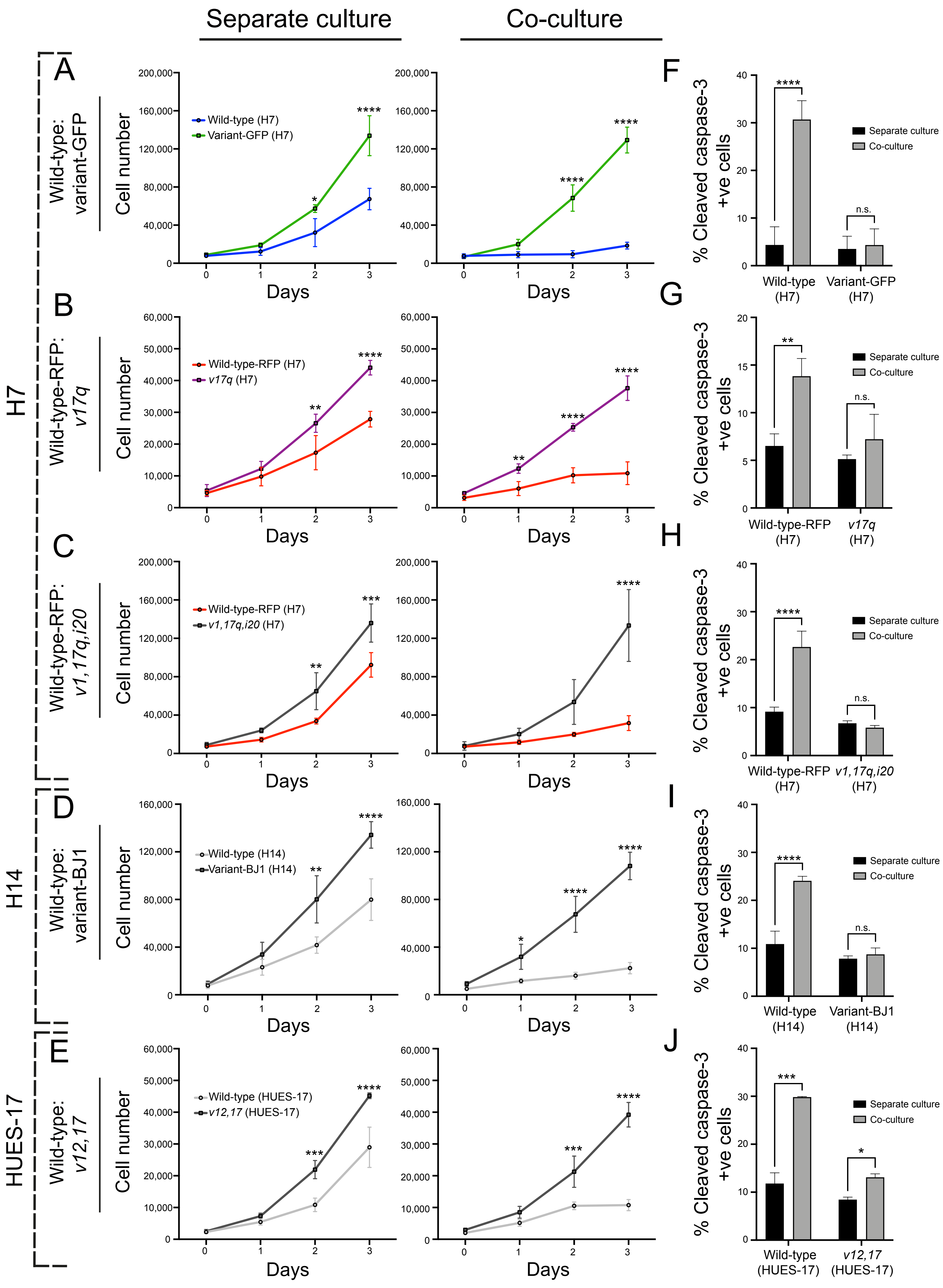

Figure S1. Wild-type cells are eliminated from co-cultures with variant hPSCs. Related to Figure 1.

A – E. Growth curves of wild-type and variant sublines of H7 (A-C), H14 (D), and HUES-17 (E) hPSC lines grown separately and in co-culture. Number of fields acquired in the well: entire well (A,C,D) or 16 random fields (B,E).

F – J. Percentage of cells positive for cleaved caspase-3 indicator of apoptosis in wild-type and variant sublines of H7 (F-H), H14 (I), and HUES-17 (J) hPSC lines grown separately or upon co-culture.

Data are the mean of three (A-I) or two (J) independent experiments ± SD. n.s. non-significant; *$p < 0.05$; **$p<0.01$; ***$p<0.001$; ****$p<0.0001$, two-way ANOVA, followed by Holm-Sidak’s multiple comparisons test.

Supplementary Figure 2

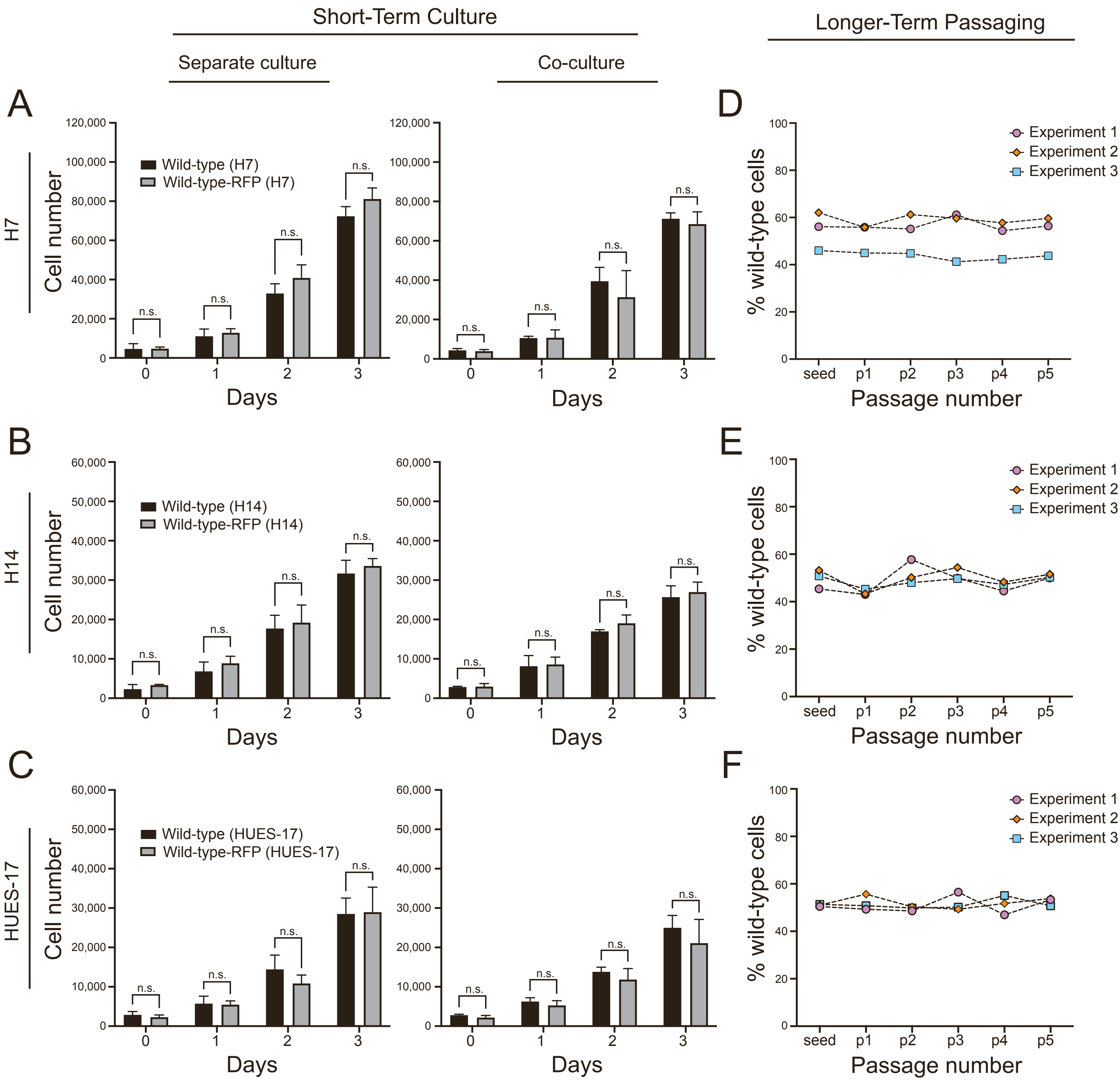

Figure S2. Wild-type hPSCs are not eliminated from co-cultures with wild-type-RFP counterparts. Related to Figure 1.

A - C. Growth curves of wild-type and wild-type-RFP sublines of H7 (A), H14 (B), and HUES-17 (C) hPSC lines grown separately and in co-culture over short-term three day culture period. Number of fields acquired in the well: entire well (A) or 16 random fields (B,C). Data represent the mean of three independent experiments ± SD. n.s. non-significant; two-way ANOVA followed by Holm-Sidak's multiple comparisons test.

D – F. Percentage of wild-type cells grown in co-cultures with wild-type-RFP cells of H7 (D), H14 (E), and HUES-17 (F) hPSC lines over five sequential passages. Data from three independent experiments are shown.

Supplementary Figure 3

A

Wild-type-RFP cells in co-culture with variant-GFP hPSCs

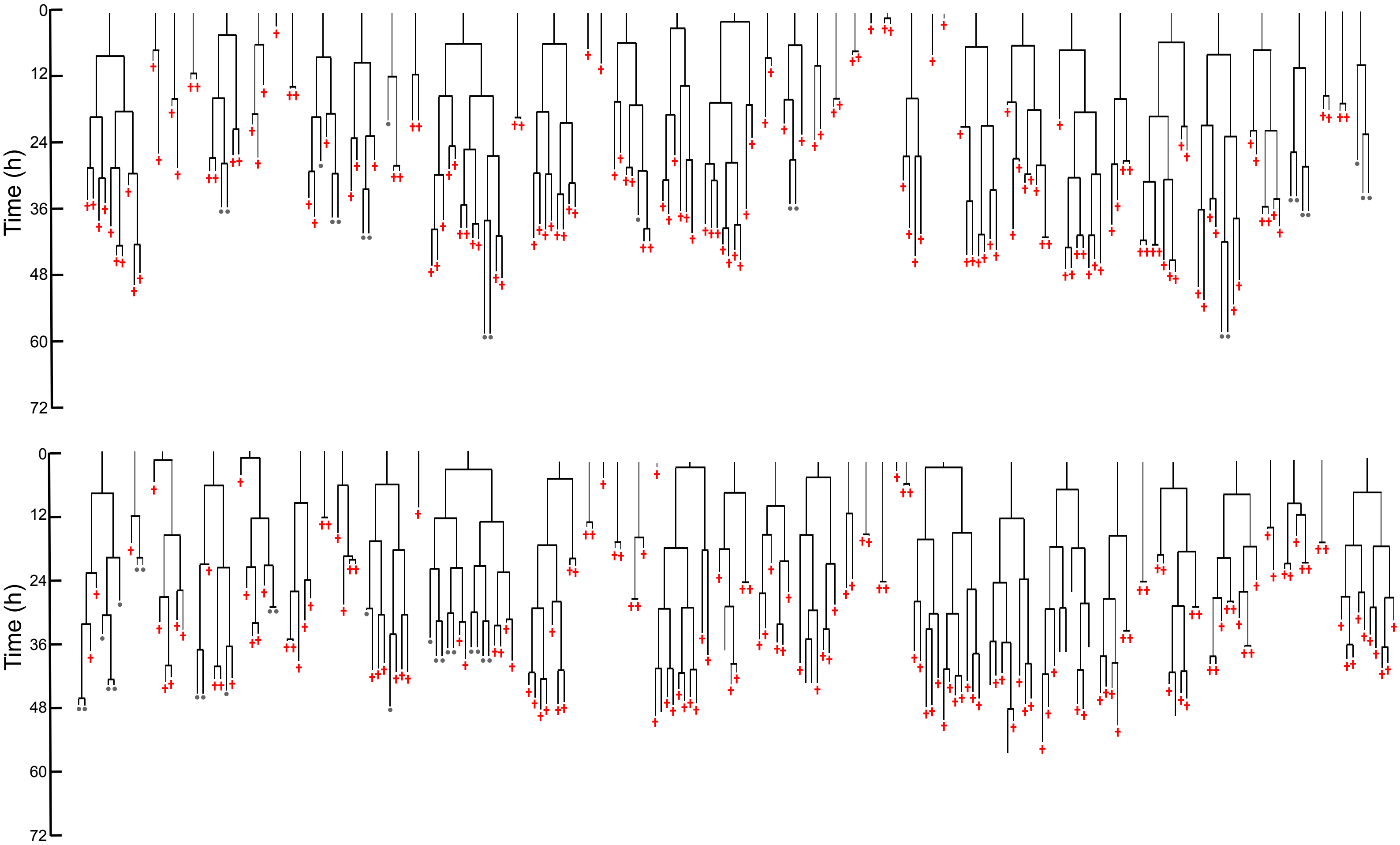

B

Wild-type-RFP cells in co-culture with wild-type hPSCs

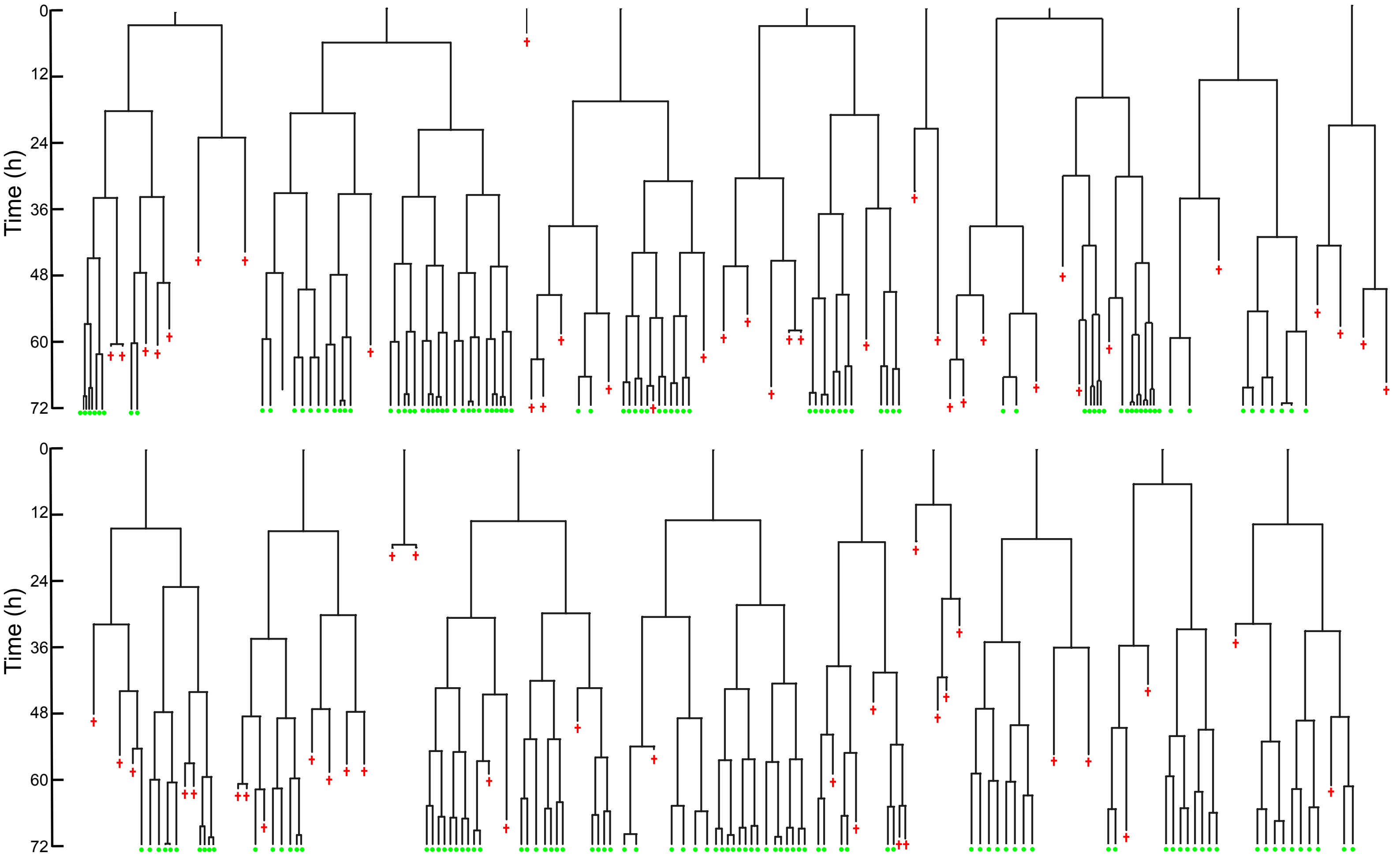

Figure S3. Lineage trees of wild-type-RFP cells co-cultured with either wild-type or variant-GFP cells. Related to Figure 1.

A-B. Lineages trees of individual wild-type-RFP cells tracked from day 0 to day 3 of co-culture with (A) variant-GFP cells or (B) wild-type-RFP. Scale indicates time in hours. Green circles indicate cells that survived and grey circles indicate cells that could no longer be accurately tracked. Red crosses denote cell death. A total of 78 (A) and 19 (B) lineage trees are shown.

Supplementary Figure 4

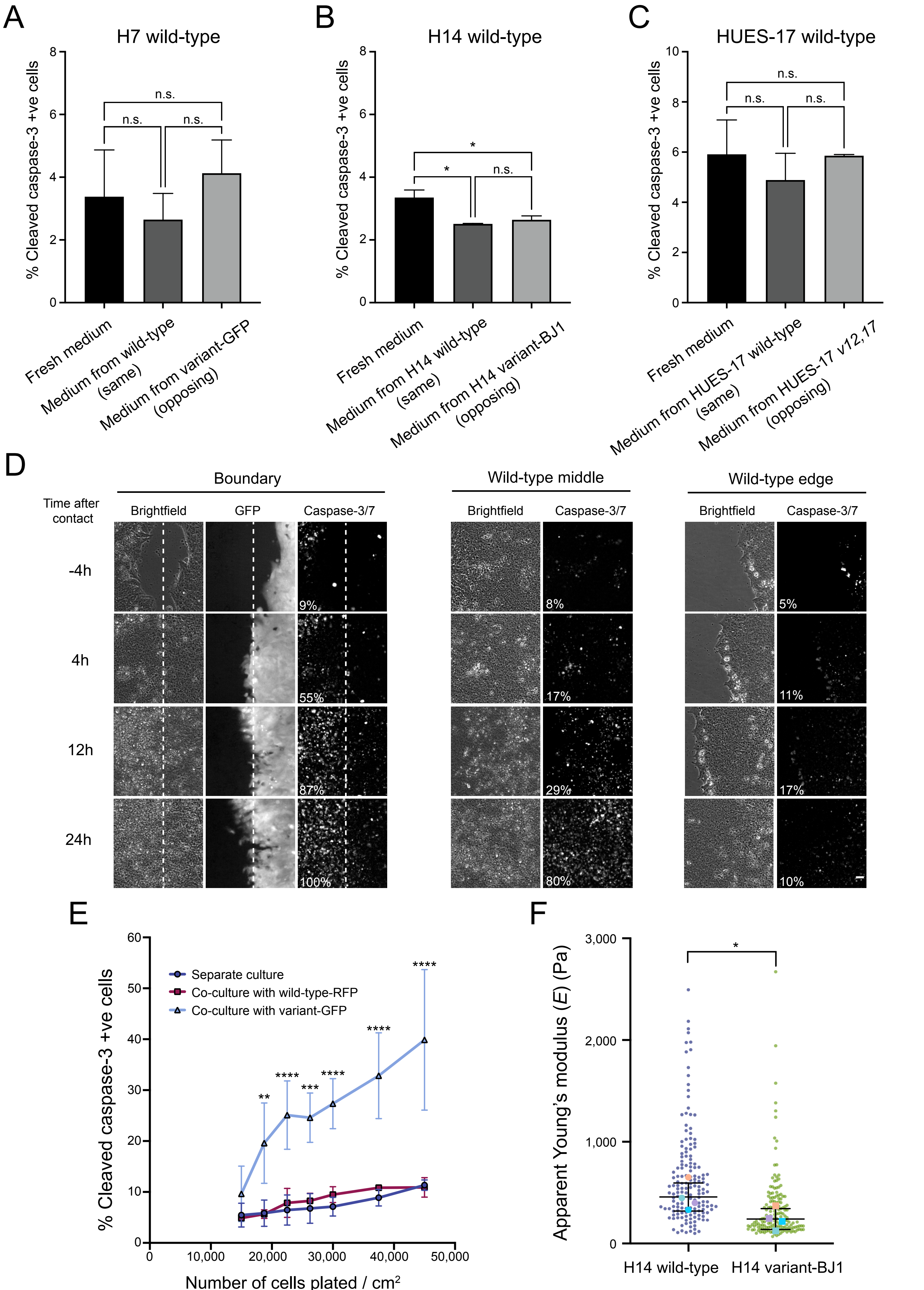

Figure S4. Winner phenotype is mediated through cell contact dependent mechanisms. Related to Figure 2 and Figure 3.

A-C. Percentage of wild-type cells positive for cleaved caspase-3 indicator of apoptosis in H7 (A), H14 (B) and HUES-17 (C) hPSC lines in conditioned media experiments. Cells were incubated either in new media, media conditioned on corresponding wild-type cells or in media conditioned by variant sublines that display a competitive phenotype. Data are the mean of three (A) or two (B,C) independent experiments ± SD. n.s. non-significant, *p<0.05; one-way ANOVA followed by Holm-Sidak's multiple comparisons test.

D. Frozen frames from the time-lapse imaging of cell confrontation assay of wild-type and variant-GFP cells with live caspase-3/7 dye. Left panel: region of the inserts where wild-type and variant-GFP cells come into contact. Dashed white line indicates the position on the insert where the two different populations meet. Middle panel: middle region of the wild-type cell population on the inserts. Right panel: edge of the wild-type cell population on the inserts. The percentage of wild-type cell area covered by live-caspase signal is indicated in the bottom-left of each image.

E. Percentage of wild-type cells positive for cleaved caspase-3 indicator of apoptosis grown either in separate culture or in co-culture with wild-type-RFP or variant-GFP cells at increasing plating densities. Data are the mean of three independent experiments ± SD. **p<0.01; ***p<0.001; ****p<0.0001; two-way ANOVA followed by Holm-Sidak's multiple comparisons test.

F. Apparent Young's Modulus (*E*) of H14 wild-type and H14 variant-BJ1 cells as measured by AFM. Small blue (wild-type) and green (variant-BJ1) points indicate individual cells, larger points indicate mean ± SD from 4 independent experiments. *p< 0.05; Student's *t* test.

Supplementary Figure 5

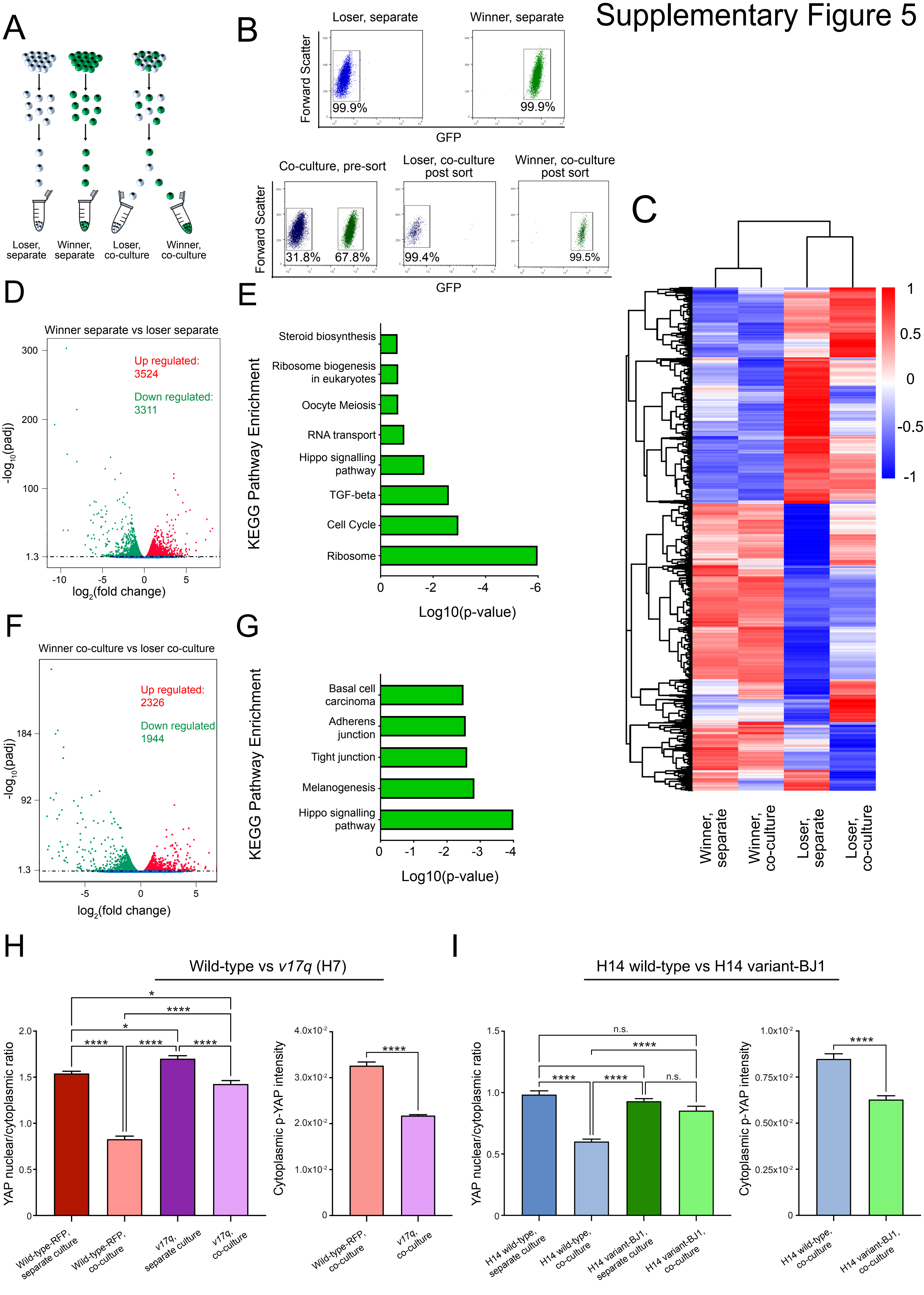

Figure S5. Gene expression analysis and immunohistochemistry of winner and loser cells indicates Hippo signalling and YAP as mediators of cell competition in hPSC cultures. Related to Figure 5.

A. Schematic depicting the sorting of loser (*v1q*) and winner (variant-GFP) cells from separate or co-culture conditions to obtain the following populations: 'loser separate', 'winner separate', 'loser co-culture' and 'winner co-culture'. Four biological replicates of each sample were obtained from independent experiments.

B. Representative flow cytometry plots of loser and winner cell populations isolated from separate or co-cultures for RNA sequencing.

C. Unsupervised hierarchical clustering of the winner and loser cells from separate and co-culture conditions based on the differentially expressed genes.

D. Volcano plot of the differentially expressed genes between winner and loser hPSCs in separate cultures. Downregulated genes (green) are positioned on the left of the plot and upregulated genes (red) are on the right of the plot.

E. KEGG pathway analysis of the downregulated genes in winner versus loser hPSCs in separate cultures.

F. Volcano plot of the differentially expressed genes between winner and loser hPSCs in co-culture. Downregulated genes (green) are positioned on the left of the plot and upregulated gene (red) are on the right of the plot.

G. KEGG pathway analysis of the downregulated genes in winner versus loser hPSCs in co-culture.

H-I. The nuclear to cytoplasmic ratio of YAP and cytoplasmic p-YAP intensity in H7 wild-type-RFP and *v17q* (H), and H14 wild-type and H14 variant-BJ1 (I) grown separately or upon co-culture. Data is represented as the mean ± SEM. *$p< 0.05$; ****$p<0.0001$; one-way ANOVA followed by Kruskal-Wallis multiple comparisons test.

Supplementary Figure 6

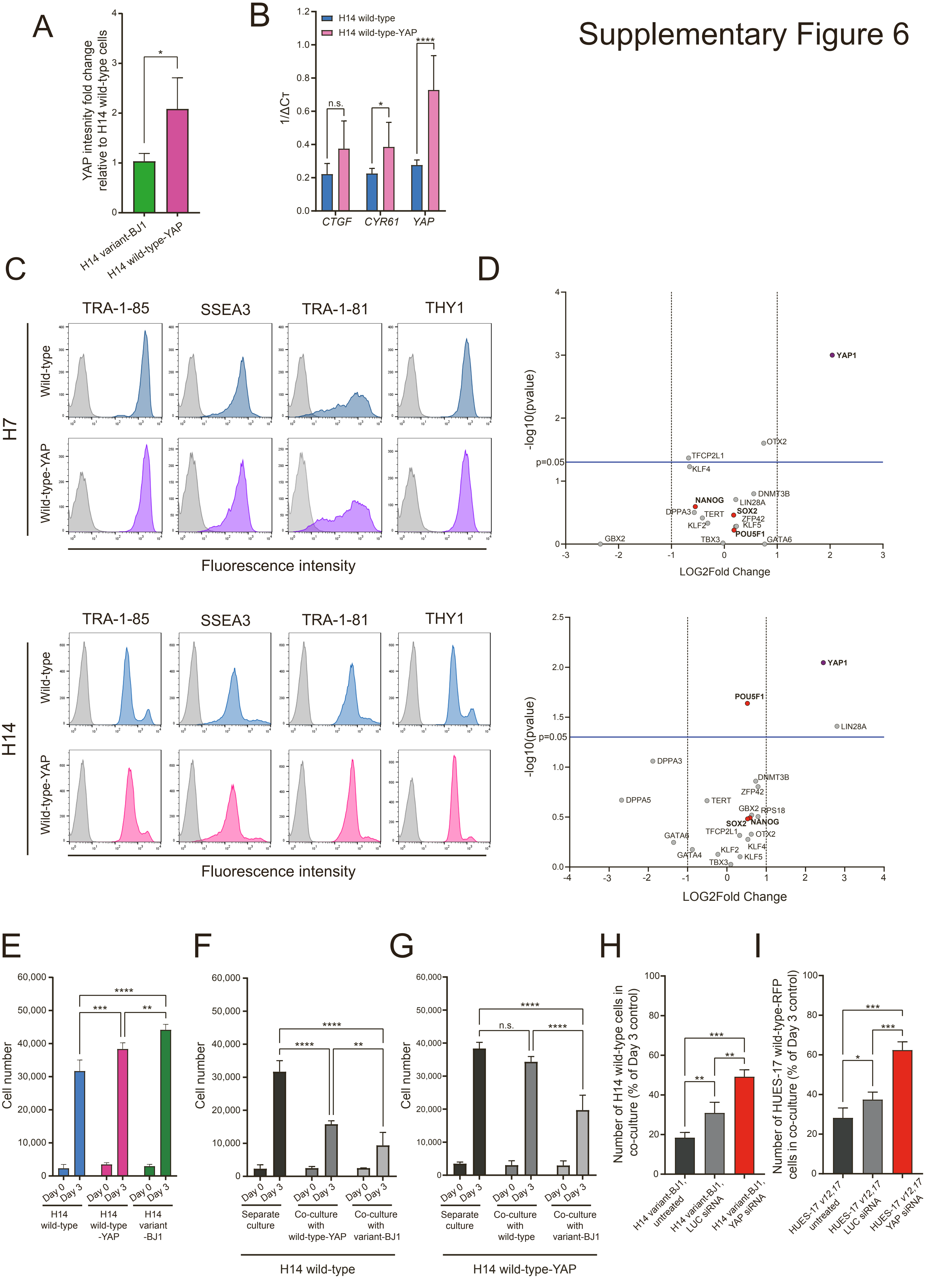

Figure S6. Manipulation of YAP affects the winner-loser phenotype, but not the pluripotency state of hPSCs. Related to Figure 5.

A. YAP overexpression in H14 wild-type hPSCs. YAP staining intensity in H14 variant-BJ1 and H14 wild-type-YAP overexpressing cells relative to the YAP staining intensity of H14 wild-type cells, as determined by flow cytometry. Data are the mean of three independent experiments ± SD. *p< 0.05; Student's *t* test.

B. Expression of YAP and its targets gene is upregulated in H14 wild-type-YAP overexpressing cells compared to H14 wild-type hPSCs. Data are the mean of three independent experiments ± SD. n.s. non-significant, *p<0.05; ****p<0.0001; one-way ANOVA followed by Fisher's LSD test.

C. YAP overexpressing cells display similar expression profiles of stem cell-associated antigens to wild-type counterparts. Flow cytometry histograms of wild-type and wild-type-YAP overexpressing cells of H7 and H14 hPSC lines for the stem cell associated antigens SSEA3, TRA-1-81 and THY1. TRA-1-85 was used as a positive control. The negative control P3X (grey) is shown in each histogram.

D. Volcano plots of the expression levels of pluripotency-associated and naïve-associated genes in wild-type-YAP overexpressing cells compared to wild-type cells of H7 and H14 hPSC lines. The core pluripotency transcription factors *SOX2*, *NANOG* and *POUF51* are denoted by red circles. Blue line indicates p=0.05 and black dashed lines indicate log2 fold change of -1 and 1.

E. YAP overexpression leads to improved growth of H14 wild-type cells. Data are the mean of three independent experiments ± SD. **p<0.01; ***p<0.001; ****p<0.0001; two-way ANOVA followed by Holm-Sidak's multiple comparisons test.

F-G. YAP overexpression confers the winner phenotype in co-cultures with H14 wild-type cells (F) and increased resistance to cell crowding in co-cultures with H14 variant-

BJ1 cells (G). Data was acquired by imaging 16 random fields within a well. Data are the mean of three independent experiments ± SD. n.s. non-significant; **p<0.01; ****p<0.0001; two-way ANOVA followed by Holm-Sidak's multiple comparisons test.

H-I. Knockdown of YAP in H14 variant-BJ1 cells (H) and HUES-17 *v12,17* cells (I) partially alleviates the winner cell phenotype of variant cells. Data are the mean of technical triplicates ± SD. n.s. non-significant; *p<0.05; **p<0.01; ***p<0.001; one-way ANOVA followed by Holm-Sidak's multiple comparisons test.

Supplementary Figure 7

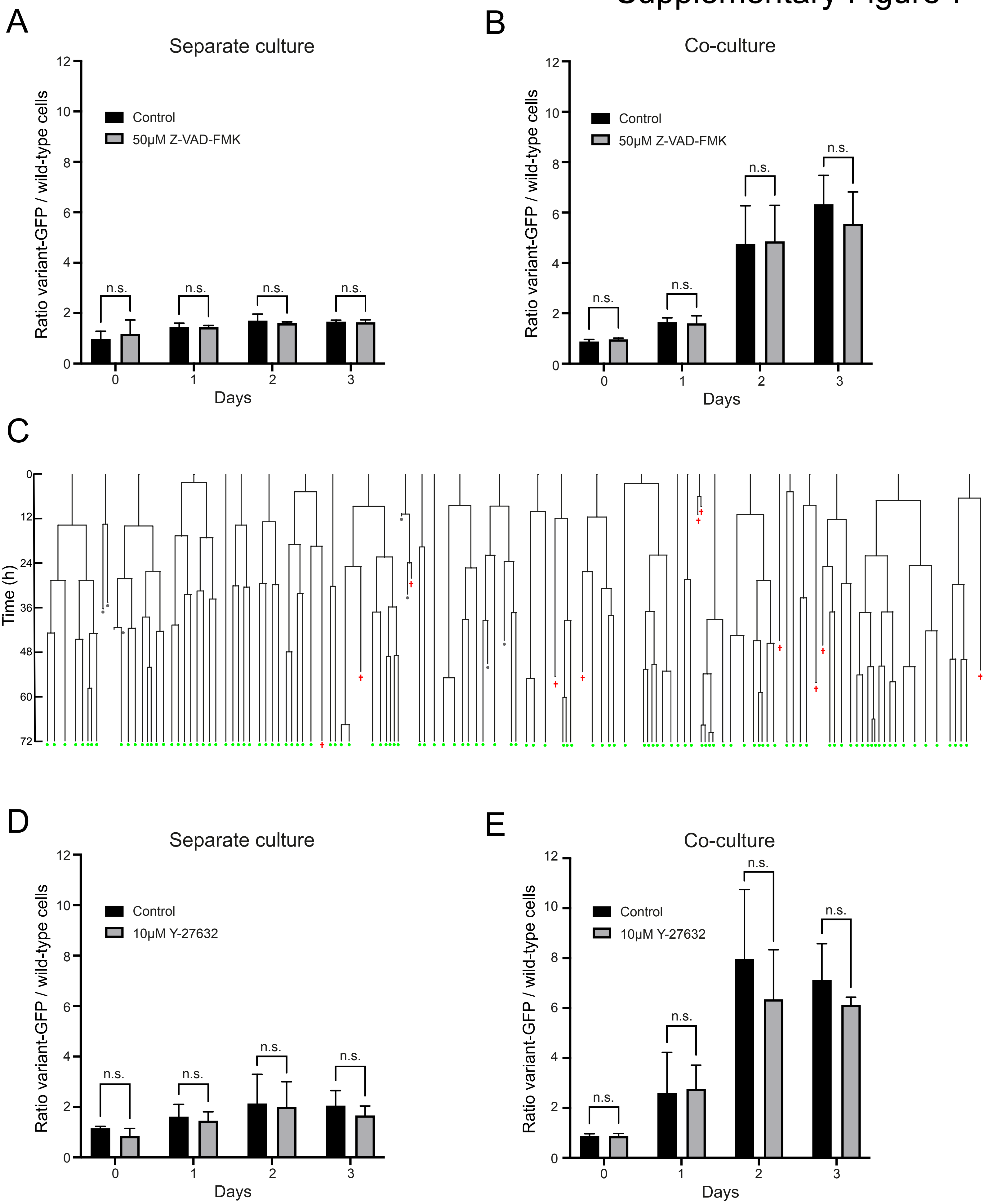

Figure S7. The common culture tools Z-VAD-FMK and Y-27632 do not restrict the competitive phenotype in hPSC cultures. Related to Figure 7.

A-B. The ratio of variant-GFP/wild-type cells grown in separate culture (A), and co-culture (B), in control or 50μM Z-VAD-FMK conditions.

C. Lineages trees of individual wild-type-RFP cells tracked from day 0 to day 3 of co-culture with variant-GFP treated with 50μM Z-VAD-FMK. Scale indicates time in hours. Green circles indicate cells that survived and grey circles indicate cells that could no longer be accurately tracked. Red crosses denote cell death. A total of 30 lineage trees is shown.

D-E. The ratio of variant-GFP/wild-type cells grown in separate culture (D), and co-culture (E), in control or 10μM Y-27632 conditions. Data are the mean of three independent experiments ± SD. n.s. non-significant; two-way ANOVA followed by Holm-Sidak's multiple comparisons test.
